# p-Type Functionalized Carbon Nanohorns and Nanotubes in Perovskite Solar Cells

**DOI:** 10.1021/acsami.3c07476

**Published:** 2023-09-06

**Authors:** Helena Uceta, Andrea Cabrera-Espinoza, Myriam Barrejón, José G. Sánchez, Edgar Gutierrez-Fernandez, Ivet Kosta, Jaime Martín, Silvia Collavini, Eugenia Martínez-Ferrero, Fernando Langa, Juan Luis Delgado

**Affiliations:** † Instituto de Nanociencia, Nanotecnología y Materiales Moleculares (INAMOL), Universidad de Castilla-La Mancha, Avenida Carlos III S/N, Toledo 45071, Spain; ‡ POLYMAT, University of the Basque Country UPV/EHU, Avenida Tolosa 72, Donostia/San Sebastián 20018, Spain; § Institute of Chemical Research of Catalonia-The Barcelona Institute of Science and Technology (ICIQ-BIST), Avinguda Països Catalans 16, Tarragona 43007, Spain; ∥ CIDETEC, Basque Research and Technology Alliance (BRTA), Paseo Miramón 196, Donostia/San Sebastián 20014, Spain; ⊥ Ikerbasque, Basque Foundation for Science, Bilbao 48013, Spain

**Keywords:** carbon nanomaterials (CNMs), carbon nanohorns (CNHs), single-walled carbon nanotubes (SWCNTs), double-walled carbon nanotubes (DWCNTs), spiro-OMeTAD, p-type doping, additives, perovskite solar cells (PSCs)

## Abstract

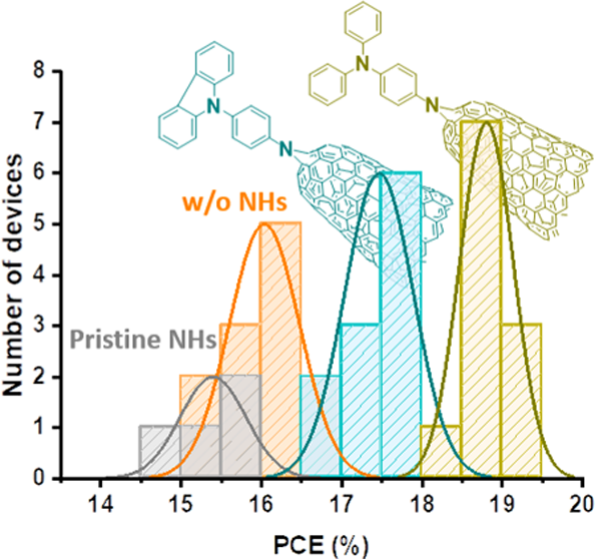

The incorporation of p-type functionalized carbon nanohorns (CNHs) in perovskite solar cells (PSCs) and their comparison with p-type functionalized single- and double-walled carbon nanotubes (SWCNTs and DWCNTs) are reported in this study for the first time. These p-type functionalized carbon nanomaterial (CNM) derivatives were successfully synthesized by [2 + 1] cycloaddition reaction with nitrenes formed from triphenylamine (TPA) and 9-phenyl carbazole (Cz)-based azides, yielding CNHs-TPA, CNHs-Cz, SWCNTs-Cz, SWCNTs-TPA, DWCNTs-TPA, and DWCNTs-Cz. These six novel CNMs were incorporated into the spiro-OMeTAD-based hole transport layer (HTL) to evaluate their impact on regular mesoporous PSCs. The photovoltaic results indicate that all p-type functionalized CNMs significantly improve the power conversion efficiency (PCE), mainly by enhancing the short-circuit current density (*J*_sc_) and fill factor (FF). TPA-functionalized derivatives increased the PCE by 12–17% compared to the control device without CNMs, while Cz-functionalized derivatives resulted in a PCE increase of 4–8%. Devices prepared with p-type functionalized CNHs exhibited a slightly better PCE compared with those based on SWCNTs and DWCNTs derivatives. The increase in hole mobility of spiro-OMeTAD, additional p-type doping, better energy alignment with the perovskite layer, and enhanced morphology and contact interface play important roles in enhancing the performance of the device. Furthermore, the incorporation of p-type functionalized CNMs into the spiro-OMeTAD layer increased device stability by improving the hydrophobicity of the layer and enhancing the hole transport across the MAPI/spiro-OMeTAD interface. After 28 days under ambient conditions and darkness, TPA-functionalized CNMs maintained the performance of the device by over 90%, while Cz-functionalized CNMs preserved it between 75 and 85%.

## 1 Introduction

**Figure 1:**
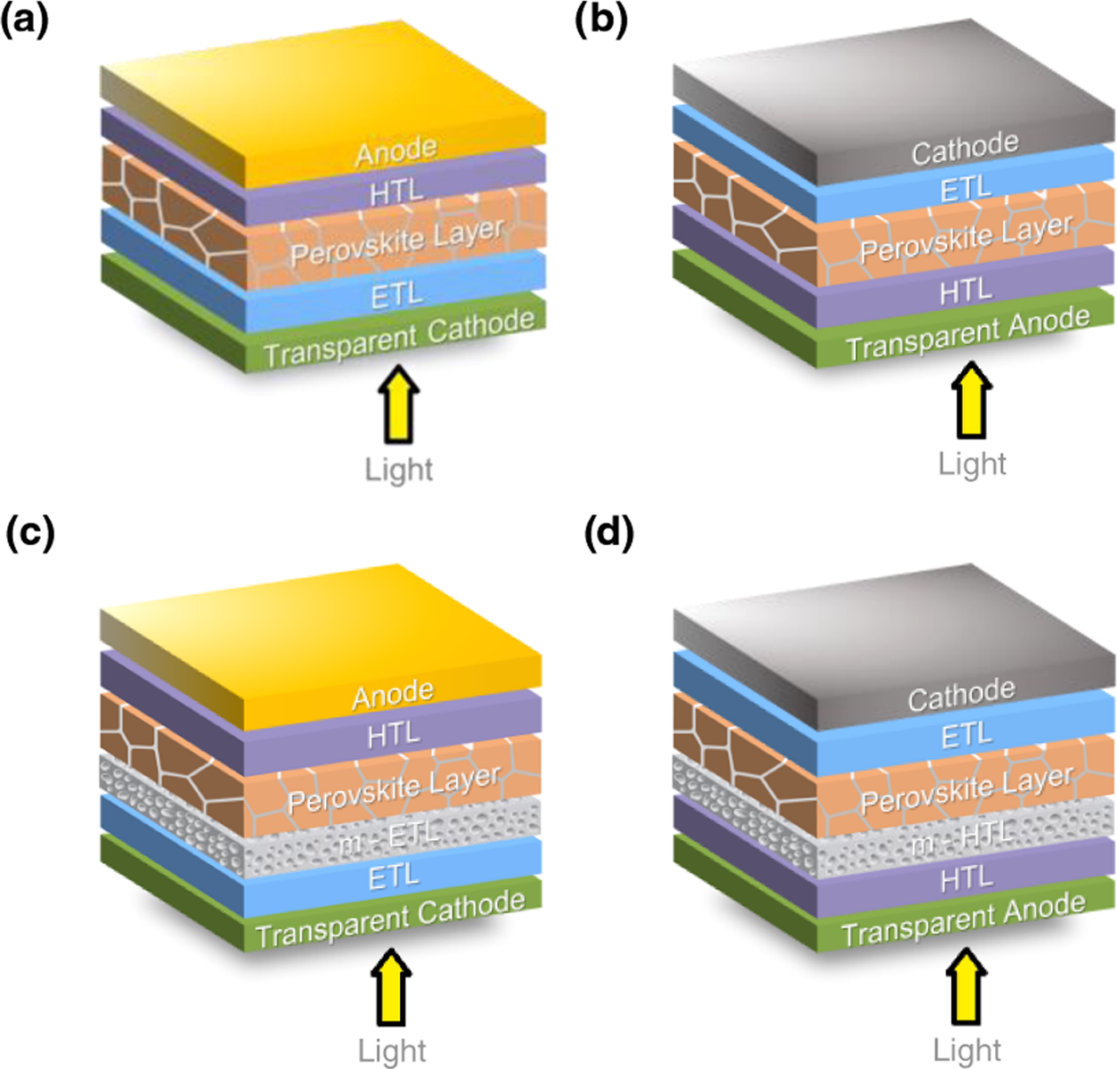
Device architectures of perovskite solar cells (PSCs): (a) regular, (b) inverted, (c) mesoporous regular, and (d) mesoporous inverted.

Perovskite solar cells (PSCs) are widely recognized as one of the most promising photovoltaic technologies to replace conventionally manufactured silicon-based solar cells. Typically, PSCs consist of an active perovskite layer sandwiched between a hole transport layer (HTL) and an electron transport layer (ETL). These charge transport layers are arranged in proximity to electrodes, with the cathode located close to the ETL and the anode near the HTL. The design of this structure allows for the efficient conversion of sunlight into electricity by capturing and moving the charges generated in the perovskite layer. The PSCs can be classified based on their architecture as regular or inverted, depending on the order in which the light passes through the device. In a regular PSC, light goes through the transparent cathode first, while in an inverted PSC, light first passes through the transparent anode, as illustrated in Figure 1.^1^ Mesoporous PSCs are a type of solar cells in which a mesoporous material is used as a selective charge transport material in contact with the perovskite layer. Usually, the mesoporous materials have pores on the nanometer scale, creating a larger area of contact with the perovskite, which leads to better device performance. Commonly, the regular architectures use mesoporous electron transport materials (m-ETM) such as m-TiO_2_ or m-SiO_2_, while inverted PSCs often employ NiO-based mesoporous hole transport materials (m-HTM).^2^

In recent years, the incorporation of carbon nanomaterials (CNMs) has been explored to enhance the performance and stability of PSCs.^3^ Several CNMs such as fullerenes, single-walled and multiwalled carbon nanotubes (SWCNTs and MWCNTs),^4^ carbon dots and their nitrogen-doped variants,^5,6^ and graphenes^7^ are, so far, the CNMs that have been studied for their application in PSCs. Fullerenes are often used as organic ETMs, but they have also been utilized as a core in the design of organic HTMs.^8^ The fullerenes and the other aforementioned CNMs have also been employed as interlayers,^9^ as well as additives in the perovskite layer,^10−17^ and in the charge transport layers.^9,18−22^ Due to their exceptional properties, such as outstanding electrical conductivity and high stability, the incorporation of CNMs into PSCs has positively impacted the device performance and stability, enhancing the generated photocurrent and stability against moisture damage.^23,24^

Single-walled carbon nanohorns (CNHs) may be considered promising CNMs for their application in PSCs. CNHs are horn-shaped aggregates of graphene sheets,^25^ with a structure analogous to SWCNTs assembled to form dahlia-like spherical aggregates with diameters between 50 and 100 nm. Since their preparation at room temperature does not require the use of catalysts, CNHs are essentially metal-free materials, and pure CNHs showing high thermal stability and semiconducting character are easily available. All of these unique properties have led to widespread interest in the use of CNHs in applications such as sensing, gas storage, batteries, or dye-sensitized solar cells.^26−28^ However, the use of CNHs in PSCs is still unknown.

On the other hand, exohedral functionalization of CNMs, including both covalent and noncovalent methods, has been tremendously helpful in obtaining derivatives with improved solubility and dispersion, as well as improved electronic properties that have been tuned to address the remaining challenges of perovskite-based devices. For instance, doped spiro-OMeTAD, the most common HTM in regular PSCs, requires dopants such as bis(trifluoromethane)sulfonimide lithium salt (Li-TFSI), tris(2-(1*H*-pyrazol-1-yl)-4-*tert*-butylpyridine)cobalt(II)di[bis(trifluoromethane)sulfonimide] (FK209), and 4-*tert*-butylpyridine (*t*-BP) to improve its hole mobility. However, these dopants generate current leakage and are highly hygroscopic, promoting the degradation of the device over time.^29^ To address these issues, carbon dots and graphene functionalized with carboxyl,^19^ primary amines,^9,20^ hydroxyl,^21^ and epoxy moieties^22^ have been used as additional additives to improve device stability and hole mobility and adjust its energy levels for more efficient charge transport. Pristine SWCNTs and MWCNTs have been integrated into the HTLs in different ways: (1) as spiro-OMeTAD HTL dopants; (2) as dopants in poly(3-hexylthiophene-2,5-diyl) (P3HT) through noncovalent wrapping, and (3) encapsulated into poly(methyl methacrylate) (PMMA).^4,30−34^ However, examples, where spiro-OMeTAD has been doped with pristine SWCNTs and MWCNTs have reported the best device photovoltaic parameters so far, while reports of covalent functionalization present worsened photovoltaic parameters.^35−37^

Table 1 collects an overview of the most efficient devices reported in the literature that utilize carbon nanotubes (CNTs) as HTLs dopants, including the types of CNTs used in the device architecture, the final structural configuration of the PSCs, and the corresponding photovoltaic parameters. It is important to remark here that those cases where covalent modification is accomplished are based on disruptive approaches, where the electronic properties of the CNTs are clearly negatively affected.

**Table 1:** Carbon Nanotube (CNT) Derivatives Used as Additive in HTLs

		photovoltaic parameters				
CNT derivatives	device architecture	*J*_sc_ (mA cm^–2^)	*V*_oc_ (V)	FF (%)	PCE (%)	year
SWCNTs	FTO/c-TiO_2_/Al_2_O_3_/MAPbI_3–*x*_Cl_*x*_/P3HT/SWCNTs:spiro-OMeTAD/Ag	21.4	1.02	71	15.4	2014^30^
SWCNTs	FTO/Al_2_O_3_/MAPbI_3–*x*_Cl_*x*_/P3HT/SWCNTs:PMMA/Ag	22.7	1.02	66	15.3	2014^4^
MWCNTs	FTO/c-TiO_2_/MAPbI_3_/spiro-OMeTAD/MWCNTs:spiro-OMeTAD/Au	21.6	1.13	69	15.1	2015^31^
BCNTs	FTO/c-TiO_2_/m-TiO_2_/MAPbI_3_/BCNTs:P3HT/Au	18.7	0.86	52	8.3	2015^32^
MWCNTs	FTO/c-TiO_2_/m-TiO_2_/CsPbI_2_Br/MWCNTs:P3HT/carbon	13.3	1.21	62	10.1	2019^33^
SWCNTs	FTO/SnO_2_/FA_0.83_Cs_0.17_Pb(I_0.9_Br_0.1_)_3_/SWCNTs:EVA:spiro-OMeTAD/Ag	22.4	1.10	69	16.8	2019^34^
MWCNTs	FTO/SnO_2_/FA_0.83_Cs_0.17_Pb(I_0.9_Br_0.1_)_3_/MWCNTs:EVA:spiro-OMeTAD/Ag	22.0	1.07	72	17.1	
MWCNTs-COOH	FTO/TiO_2_/MAPbI_3_/MWCNTs-COOH:spiro-OMeTAD/Au	18.0	0.84	58	8.7	2016^35^
(7,6)-SWCNTs-OPV	FTO/c-TiO_2_/MAPbI_3_/spiro-OMeTAD/(7,6)-SWCNTs-OPV:spiro-OMeTAD/Au	16.6	1.00	58	9.6	2016^36^
SWCNTs-PhOMe	FTO/c-TiO_2_/m-TiO_2_/MAPbI_3_/SWCNT-PhOMe:P3HT/Au	22.0	0.85	62	11.6	2016^37^

**Scheme 1:**
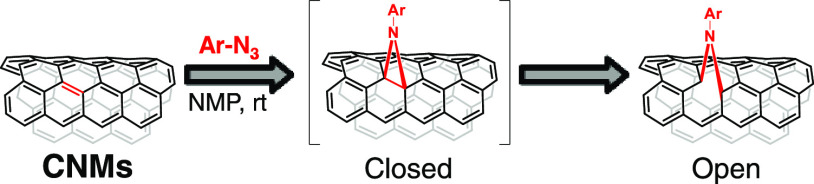
Schematic Representation of the Reaction between Azides and Carbon Nanomaterials (CNMs), and Subsequent Rearrangement to the Open Structure

As shown in Table 1, the majority of research studies using CNMs as additives for HTMs in PSCs predominantly use pristine SWCNTs and MWCNTs. Limited investigations have been conducted to explore the potential advantages of functionalized CNMs, and to the best of our knowledge, there are no existing reports on the use of both pristine and functionalized carbon nanohorns (CNHs) and double-walled carbon nanotubes (DWCNTs). This study presents, for the first time, the application of both functionalized CNHs and functionalized DWCNTs as dopants for spiro-OMeTAD. Furthermore, the corresponding results obtained using functionalized CNHs and functionalized DWCNTs are presented and compared with those of the functionalized counterparts of SWCNTs. Motivated by the promising prospects of functionalized CNMs, this study also examines the impact of using two different p-type addends, namely, triphenylamine (TPA) and 9-phenyl carbazole (Cz) units. These electron-donor moieties based on ternary nitrogen have not been covalently linked to these types of CNMs previously. The six new functionalized CNMs were synthesized by [2 + 1] cycloaddition reaction of CNMs with nitrenes, generated in situ from the corresponding TPA and Cz azides (TPA-N_3_ and Cz-N_3_).^38^ The [2 + 1] cycloaddition was specifically chosen because this method preserves the π-conjugated system of the sp^2^ network,^40^ in contrast to typical [3 + 2] or [2 + 2] cycloadditions, which result in a change of carbon hybridization from sp^2^ to sp^3^ in the chemical structure of carbon CNMs.^39^ It is a known fact that the exceptional electronic properties of π-systems depend heavily on the preservation of their structure. Interestingly, through the mentioned approach, the modified CNM preserves the electronic properties of the pristine structure as the initially formed three-membered ring bridge (hereafter referred to as the closed structure) evolves, opening the bridge and recovering the π-conjugated system (hereafter referred to as the open structure), as shown in Scheme 1. In this regard, theoretical calculations have supported the higher stability of the open structure CNM-derivatives compared to the closed ones.^41^

**Scheme 2:**
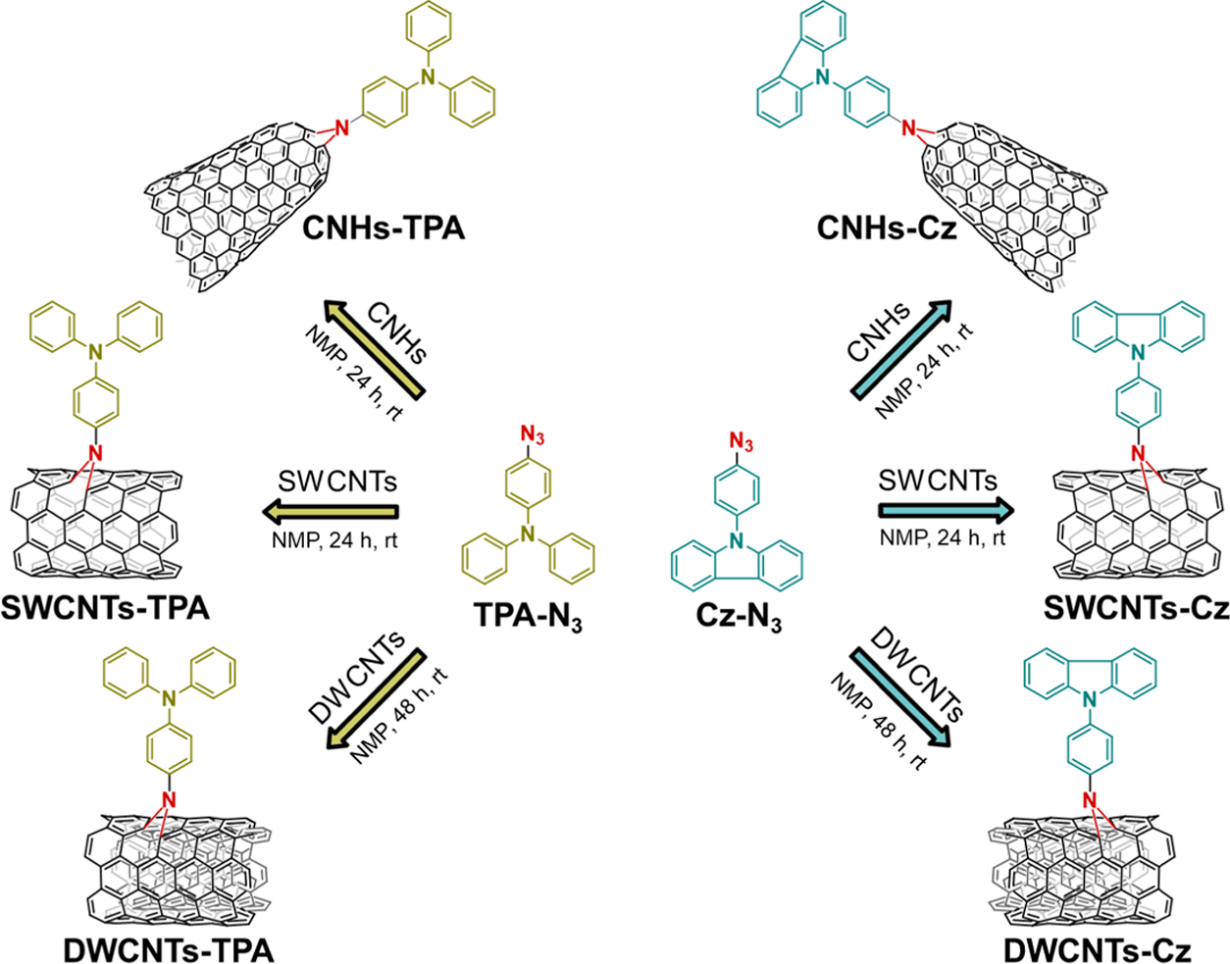
Synthesis of Functionalized Carbon Nanomaterials (CNMs) Incorporated in the Spiro-OMeTAD Layer

Thus, single-walled carbon nanohorns-triphenylamine (CNHs-TPA), single-walled carbon nanohorns-carbazole (CNHs-Cz), single-walled carbon nanotubes-triphenylamine (SWCNTs-TPA), single-walled carbon nanotubes-carbazole (SWCNTs-Cz), double-walled carbon nanotubes-triphenylamine (DWCNTs-TPA), and double-walled carbon nanotubes-carbazole (DWCNTs-Cz) have been prepared following the procedure outlined in Scheme 2. The successful functionalization was confirmed by various techniques, including thermogravimetric analysis (TGA), UV–vis-NIR absorption and Raman spectroscopies, photoluminescence excitation intensity mapping (PLE), and X-ray photoelectron spectroscopy (XPS). These materials were then incorporated into the doped spiro-OMeTAD layer in regular mesoporous PSCs, and their effect on photovoltaic performance was evaluated by means of the current–voltage (*J*–*V*) curves, external quantum efficiency (EQE), space-charge limited current (SCLC) method, photoluminescence measurements, UV–vis absorption spectroscopy, cyclic voltammetry, field emission scanning electron microscopy (FE-SEM), grazing-incidence wide-angle X-ray scattering (GIWAX) analysis, and stability tests. The results demonstrate that incorporating all functionalized CNMs resulted in improvements in power conversion efficiency (PCE), mainly by enhancing the short-circuit current density (*J*_sc_) and fill factor (FF). The enhancement of these parameters was due to the increase in the hole mobility of spiro-OMeTAD, a higher oxidized spiro-OMeTAD concentration, improved energy alignment with the perovskite layer, and improved morphology and contact interface. The addition of CNH derivatives resulted in a slightly higher increase in performance in comparison to SWCNTs and DWCNTs derivatives. Upon comparing the two functional groups linked to CNMs, those containing TPA provided outcomes superior to those containing Cz moieties. Similarly, an analysis of the nonfunctionalized CNMs (CNHs, SWCNTs, DWCNTs) was conducted, but no significant improvement was observed, thus highlighting the critical role of functionalization in enhancing device performance. In addition, it has been demonstrated that incorporating functionalized CNMs enhances device stability by improving the hydrophobic capacity of the HTLs and enhancing the hole transport across the MAPI/spiro-OMeTAD interface. TPA-functionalized-based devices maintained over 90% of their performance, while those with Cz-functionalized CNMs retained between 75 to 85% of their initial value. Among the Cz-functionalized-based devices, those with CNH-Cz exhibited the highest stability.

## 2 Results and Discussion

### 2.1 Synthesis and Characterization of the p-Type Functionalized Carbon Nanomaterials (CNMs)

The synthesis of the target functionalized CNMs was performed according to the procedure outlined in Scheme 2. 4-Azido-*N*,*N*-diphenylaniline (TPA-N_3_) and 9-(4-azidophenyl)-9*H*-carbazole (Cz-N_3_) were synthesized following a slightly modified procedure described in the literature (Scheme S1 and Figures S1–S10 in the Supporting Information for further details).^42−44^ The covalent modification of the different CNMs via [2 + 1] nitrenes cycloaddition was accomplished. In brief, to a suspension of the pristine carbon nanostructure (SWCNTs, DWCNTs, or CNHs) in *N*-methyl-2-pyrrolidone (NMP), TPA-N_3_ or Cz-N_3_ was added. The reactions were run at room temperature under an argon atmosphere for 24 h for SWCNTs and CNHs, and 48 h for DWCNTs (see further details in Section 4). The functionalized CNMs were then recovered by filtration through a poly(tetrafluoroethylene) (PTFE) membrane with a pore size of 0.45 μm, washed with NMP and CH_2_Cl_2_, and dried overnight, yielding the desired functionalized CNMs.

**Figure 2:**
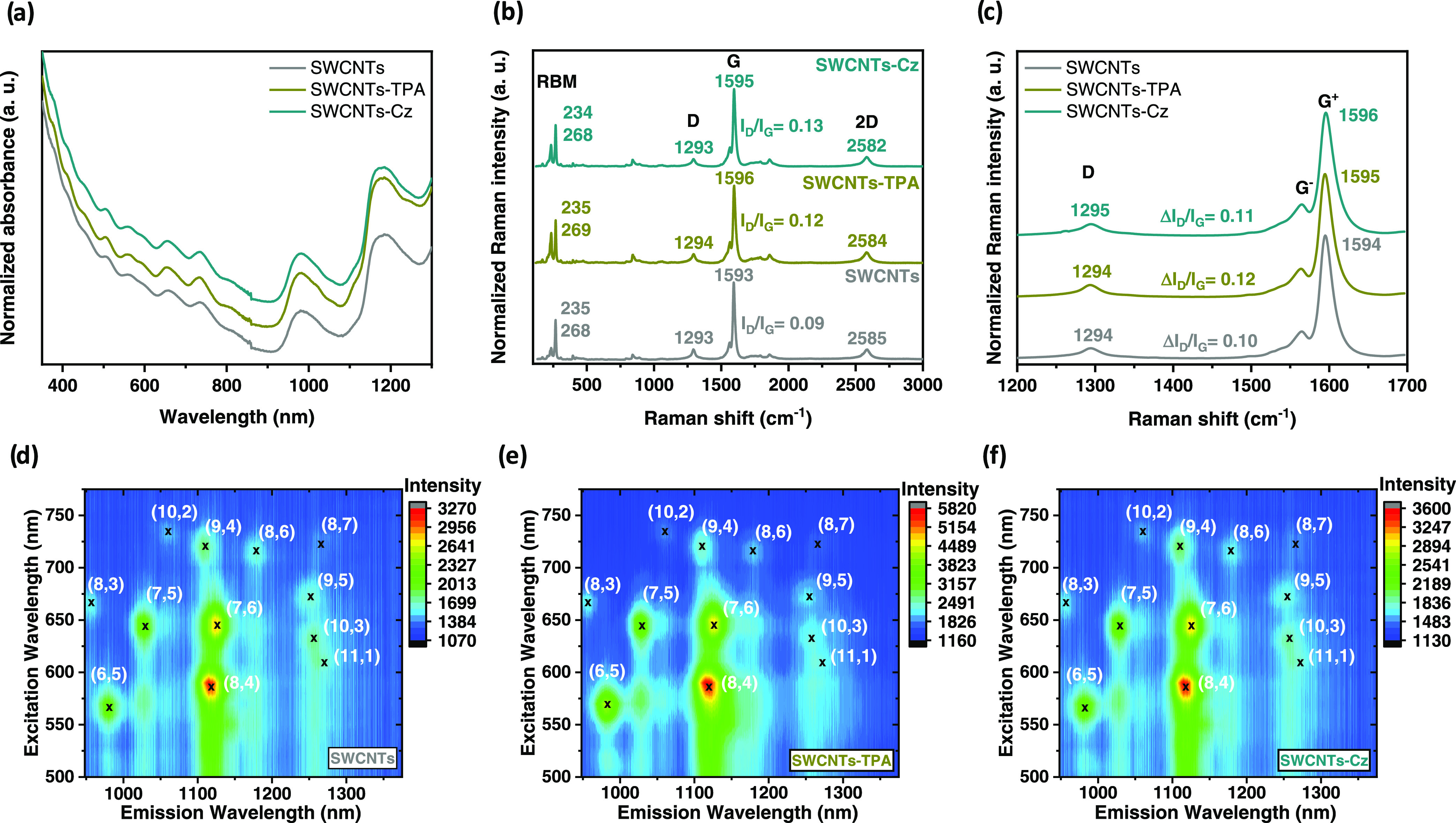
(a) UV–vis-NIR absorption spectra of pristine SWCNTs compared to SWCNTs-TPA and SWCNTs-Cz. (b, c) Raman extended and Raman map average zone spectra of pristine SWCNTs compared to SWCNTs-TPA and SWCNTs-Cz (laser excitation λ = 785 nm). (d–f) PLE maps of pristine SWCNTs compared to SWCNTs-TPA and SWCNTs-Cz. The (*n*,*m*) indices specify the SWCNT species associated with each emission spot. UV–vis-NIR absorption spectra and PLE maps were recovered in Milli-Q-grade water containing 10% weight of SDBS as a surfactant at room temperature.

The first evidence of successful covalent modification on the CNMs was evaluated through thermogravimetric analysis-derivative thermogravimetry (TGA-DTG) under a nitrogen atmosphere (Figure S11 and Table S1, Supporting Information). The TGA-DTG studies confirm the presence of functional groups anchored to the surface of the different CNMs, as deduced from the pattern of the weight loss compared with the pristine materials at 650 °C, where it is considered that the organic addends have been completely removed from the CNMs sidewalls. TGA curves can be used to estimate the functional group coverage (FGC) for each hybrid structure, according to the equation described in the Supporting Information. The FGC for each functionalized CNMs was calculated, and the resulting data are presented in Table S1. These results show the presence of higher loading of addends in the case of SWCNT-based hybrid materials when compared to DWCNTs. This fact can be attributed to the smaller diameters of SWCNTs that result in a higher degree of curvature, being more reactive than wider CNTs.^45^ Full characterization of the functionalized CNMs was further carried out by means of UV–vis-NIR and Raman spectroscopies, photoluminescence excitation intensity mapping (PLE), and X-ray photoelectron spectroscopy (XPS). Clear evidence of the preservation of the π-conjugated structure was initially found in the UV–vis-NIR absorption spectra of pristine SWCNTs. The absorption spectra of SWCNT-TPA and SWCNT-Cz registered in an aqueous solution of sodium dodecylbenzene sulfonate (H_2_O-SBDS) show the characteristic van Hove singularities of carbon nanotubes, with the absorption maxima corresponding to the TPA (308 nm) or Cz (246 nm) being hidden in the high-energy region of the spectra (Figure 2a). Therefore, similarly to the features displayed by pristine SWCNTs, the functionalized CNMs show a series of bands between 400 and 700 nm, ascribed to transitions between first (M_11_) singularities of metallic SWNT, and between 700 and 950 and 950–1300 nm, attributed to semiconducting SWCNTs. Interestingly, the bands present comparable intensity to the pristine materials, which suggests that π-conjugation of the carbon network is preserved after the functionalization process. It is important to recall here that functionalization through the formation of sp^3^ C-atoms destroys the extended π-conjugated structure of CNMs, thereby disrupting the translational symmetry and changing the electronic structure of the SWNTs, bleaching the van Hove singularities.^46^

In an attempt to explore further changes in the optical absorption properties of the functionalized CNMs, UV–vis studies were accomplished in different solvents (Figure S12). It can be observed that the UV spectra of the precursors, bromotriphenylamine and 4-(9-carbazolyl)benzeneboronic acid pinacol ester in dichloromethane (DCM), exhibit absorption bands within the wavelength range of 250 to 300 nm (Figure S12a). The absorption spectra of the hybrid materials based on SWCNTs and CNHs in DCM reveal new absorption features in this region (250–300 nm) but with low intensities, mainly due to the broad absorption characteristic of CNTs that hinders the observation of the TPA and Cz features (Figure S12b,c). For DWCNTs, due to their lower dispersion quality, DCM was not suitable for performing UV–vis studies. Instead, the studies were conducted in NMP (Figure S12d), showing an increase in the absorption intensity at 270 nm. These results provide evidence for the presence of TPA and Cz moieties on the sidewalls of CNHs, SWCNTs, and DWCNTs.

Photoluminescence excitation (PLE) maps of pristine and functionalized SWCNTs were collected. PLE mapping is a powerful method for identifying specific tube chiralities (*n*,*m*) in SWCNT-based samples.^46,47^ Thus, as displayed in Figure 2d, pristine SWCNTs emit light corresponding to semiconducting (8,4) SWCNT, which has a diameter of 0.84 nm, and to a lesser extent emit as well light corresponding with (7,6) SWCNTs. Interestingly, after covalent functionalization to yield SWCNT-TPA (Figure 2e) and SWCNT-Cz (Figure 2f), the PLE emission intensities of the mentioned chiralities are comparable to that of pristine SWCNTs, indicating that, after functionalization, the π-conjugated system remains unperturbed, as it is known that the presence of sp^3^-atoms in functionalized SWCNTs quench the NIR emission.^48^

In general, the UV–vis–NIR spectra of CNHs and DWCNTs are featureless in H_2_O-SBDS dispersions, which is attributed to their nonuniform size and shape and the presence of different diameters and lengths.^49−51^ Additionally, no PLE features of these materials are normally detected, except that luminescent impurities are attributed to the presence of SWCNTs in the samples. This behavior is in line with the expected PLE quenching induced by the presence of different walls.^52,53^ Then, Raman spectra of all of the functionalized CNMs (Figures 2b,c and S13–S16) were recorded to determine the changes in the spectral features after the functionalization process and evaluate their electronic properties. In general, covalent bond formation in CNMs is accompanied by sp^3^ distortion of the sp^2^ hybridized carbon network. Consequently, the D band in the Raman spectra, which represents the change of C-atoms from the sp^2^ to the sp^3^ hybridization state, shows an increase in intensity. Figure 2b,c and Table S1 show that there was no further increase in the *I*_D_/*I*_G_ ratio (relationship between the intensity of the D- and G-band) when moving from pristine SWCNT to covalently functionalized samples, indicating that there was no conversion of carbon atoms from sp^2^ to sp^3^. Similar results were obtained for functionalized DWCNTs and CNHs when compared to their respective pristine derivatives (Figure S13 and Table S1).^54^ The *I*_D_/*I*_G_ ratio of each sample was calculated from the average of 400 independent measurements taken over the sample. Complete statistical analysis of intensity ratios and peak position is provided in the Supporting Information (Figures S13–S16). These results demonstrate that the characteristic features of the functionalized CNMs and pristine ones are nearly identical, without any significant shifts (<3 cm^–1^), confirming once again the preservation of the π-conjugated structural integrity as suggested by UV–vis–NIR and PLE spectroscopy experiments. It is important to remark here also the small shift to higher frequencies observed in the G+ mode after the covalent modification of the SWCNTs via [2 + 1] nitrene cycloaddition, which is indicative of the p-type doping effect of TPA and Cz addends, suggesting an increase in the hole charge carriers and also their hole-transporting semiconductor properties likely to act as hole selective p-type charge collection layers. The same trend is observed for DWCNTs and CNHs (Figures S13, S15, and S16).^55,56^

X-ray photoelectron spectroscopy (XPS) is a powerful technique that can be used to identify elements present on the surface of a sample and, therefore, is one of the best techniques for studying the chemical modification of CNMs. Figure S17 in the Supporting Information shows the survey spectra and high-resolution XPS data for pristine and functionalized CNMs. Furthermore, the percentages of each chemical element are summarized in Table S2 (Supporting Information). These results were used to analyze the elemental composition of each material, where three important features deserve particular attention. (i) The survey spectra of the pristine materials are characterized by the presence of carbon (C 1s) and oxygen (O 1s), while those corresponding to the functionalized samples show the presence of a new peak at ∼400 eV, typically assigned to nitrogen (N 1s). This can be univocally attributed to the anchorage of TPA and Cz moieties to the surface of the CNMs. It is important to note here that molybdenum impurities have been detected in the pristine DWCNTs, a metal that is commonly used as a catalyst during the synthetic procedure.^57,58^ As observed in the XPS survey spectra, the N 1s region in DWCNT-based samples is overlapped by the peak attributed to Mo 3p, rendering it difficult to acquire accurate quantitative analysis of nitrogen in this case. (ii) The high-resolution N 1s spectra can be fitted into two peaks (∼399.5 and ∼400.4 eV), which can be attributed to the presence of two nitrogen species, one assigned to the nitrogen atoms found in the TPA and Cz cores, and the second, to the nitrogen-containing open structures of functionalized CNMs.

Finally, for the sake of comparison, the progress of the reaction at higher temperatures was studied. For this purpose, SWCNTs were functionalized via nitrene cycloaddition with TPA and Cz at 70 °C under identical reaction times, obtaining a slightly higher degree of functionalization but identical spectroscopic characteristics (Figures S18–S19, Supporting Information). These results confirm that mild conditions are sufficient to achieve high functional group coverages, and besides, harsher reaction conditions do not imply any worsening of the intrinsic electronic properties of CNMs.

### 2.2 Preparation and Characterization of the Perovskite Solar Cells (PSCs)

**Figure 3:**
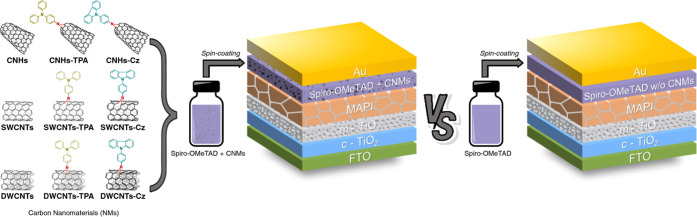
Perovskite solar cell architecture with carbon nanomaterials (CNMs) as additives for the spiro-OMeTAD- based HTLs.

To incorporate the synthesized p-type nanomaterials (CNMs) into PSCs, regular mesoporous devices were fabricated following an FTO/c-TiO_2_/m-TiO_2_/MAPI/doped spiro-OMeTAD-based HTL/Au architecture and using the solution-processing methodology. The different doped spiro-OMeTAD-based precursor solutions were obtained by preparing dispersion solutions of each CNM (functionalized CNMs: CNHs-TPA, CNHs-Cz, SWCNTs-TPA, SWCNTs-Cz, DWCNTs-TPA, and DWCNTs-Cz, as well as the pristine CNMs: CNHs, SWCNTs, and DWCNTs) in chlorobenzene and dissolving the spiro-OMeTAD and dopants in those dispersions. A control device was fabricated through a precursor solution of CNMs-free spiro-OMeTAD (labeled as “w/o CNMs”), as shown in Figure 3. Henceforth, the term spiro-OMeTAD layer will be referred to as the spiro-OMeTAD layer doped with Li-TFSI, FK209, and *t*-BP using the typical doping procedure reported in the literature.^59^ The experimental details regarding the fabrication of the devices and the preparation of the saturated solutions are found in Section 4. A picture of all of the target devices is illustrated in Figure S20.

**Figure 4:**
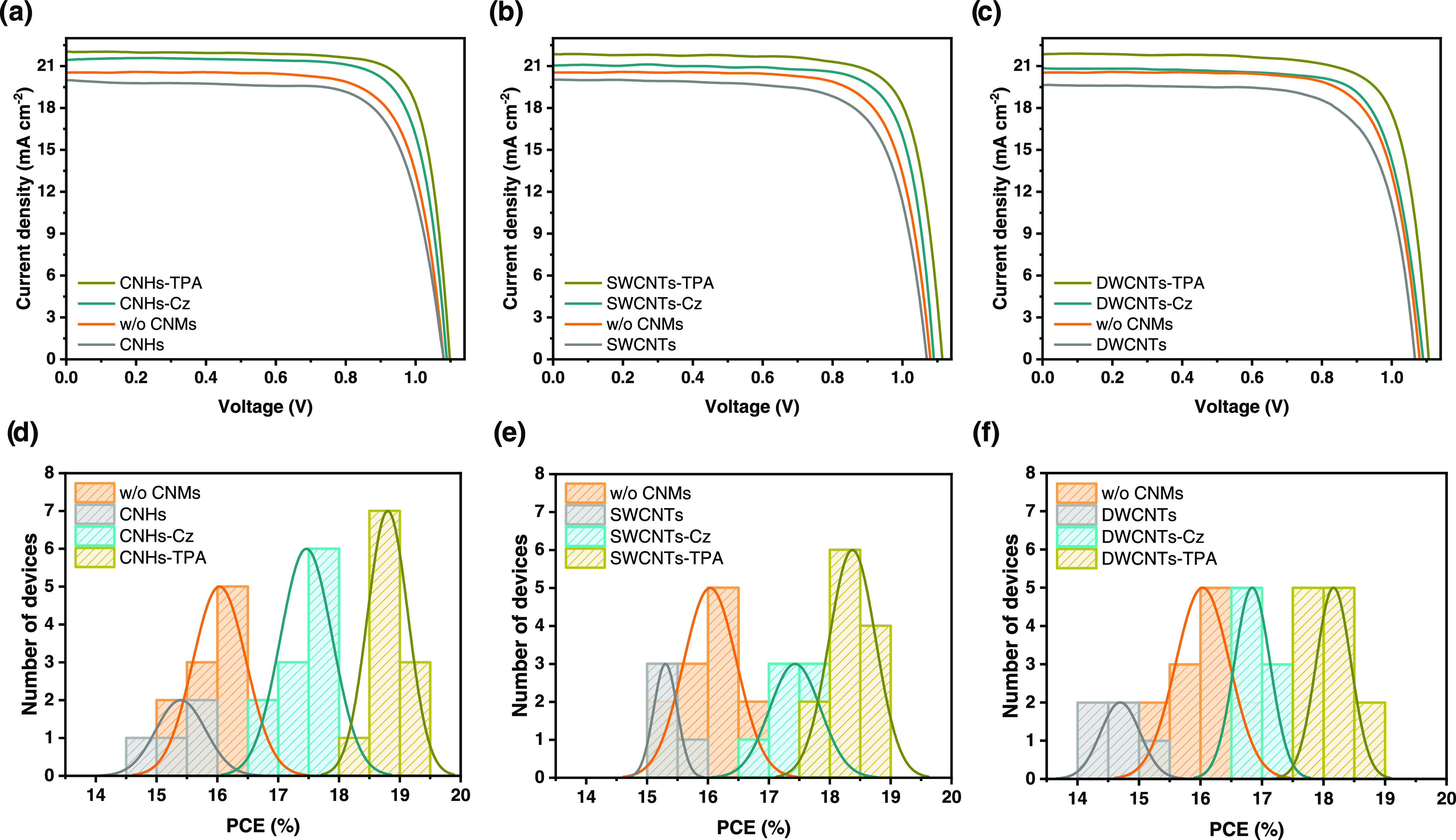
(a–c) Current density–voltage (*J*–*V*) curves for the best perovskite solar cells (PSCs) and (d–f) power conversion efficiency (PCE) distribution histograms with CNHs (a, d), SWCNTs (b, e), and DWCNTs derivatives (c, f) in the hole transport layer (HTL). Orange data represents the device without CNMs (w/o CNMs); devices with pristines are shown in gray, with carbazole (Cz) derivatives in cyan, and with triphenylamine (TPA) derivatives are in olive color (active area 0.16 cm^2^, AM 1.5G illumination, scan rate 100 mV cm^–2^).

Figure 4a–c presents the current density vs voltage (*J*–*V*) characteristics of the best-performing devices with and without CNMs in the hole transport layer (HTL). The photovoltaic parameters of all devices are listed in Table 2. Figure 4d–f displays the PCE distribution histograms, and the complete statistical analysis is provided through the box charts in Figure S21. The average values and standard deviations of the photovoltaic parameters are summarized in Table S3 in the Supporting Information.

**Table 2:** Photovoltaic Parameters for the Best Perovskite Solar Cells (PSCs) with and without Carbon Nanomaterials (CNMs) in the Hole Transport Layers (HTLs)^a^

HTL	*J*_sc_ (mA cm^–2^)	*V*_oc_ (V)	FF (%)	PCE (%)	*R*_s_ (Ω cm^–2^)^b^	*R*_sh_ (kΩ cm^–2^)^b^
w/o CNMs	20.5	1.08	75	16.6	33	18
CNHs	20.0	1.08	73	15.8	40	11
CNHs-Cz	21.5	1.09	78	17.9	28	28
CNHs-TPA	22.0	1.10	80	19.4	22	30
SWCNTs	20.0	1.07	73	15.5	44	8
SWCNTs-Cz	21.1	1.09	77	17.8	30	20
SWCNTs-TPA	21.9	1.11	78	19.0	24	24
DWCNTs	19.7	1.07	72	15.2	48	8
DWCNTs-Cz	20.8	1.09	76	17.3	31	13
DWCNTs-TPA	21.9	1.11	77	18.6	24	22

a Photovoltaic parameters, including short-circuit current density (*J*_sc_), open-circuit voltage (*V*_oc_), fill factor (FF), power conversion efficiency (PCE), series resistance (*R*_s_), and shunt resistance (*R*_sh_).

b Estimated from a fit of the respective *J*–*V* curve.

The photovoltaic results reveal that including p-type CNMs in the spiro-OMeTAD layer has a significant effect on the performance of PSCs. The control device without CNMs showed a maximum efficiency of 16.6%, with an open-circuit voltage (*V*_oc_) of 1.08 V, a short-circuit current density (*J*_sc_) of 20.5 mA cm^–2^, and a fill factor (FF) of 75%. In comparison, all of the functionalized CNMs improved the *J*_sc_ and FF, resulting in a higher power conversion efficiency (PCE).

The device with the TPA-functionalized CNHs (CNHs-TPA) exhibited the best PCE at 19.4%, with *J*_sc_ of 22.0 mA cm^–2^, *V*_oc_ of 1.10 V, and FF of 80%. Meanwhile, the device with Cz-functionalized analogue (CNHs-Cz) showed a maximum efficiency of 17.9% with *J*_sc_ of 21.5 mA cm^–2^, *V*_oc_ of 1.09 V, and FF of 78%. The PSCs based on SWCNTs-TPA presented a higher device efficiency with a PCE of 19.0% (*J*_sc_: 21.9 mA cm^–2^, *V*_oc_: 1.11 V, FF: 78%), compared to the one based on SWCNTs-Cz with a lower PCE of 17.8% (*J*_sc_: 21.1 mA cm^–2^, *V*_oc_: 1.09 V, FF: 77%). The device using TPA-functionalized DWCNTs displayed an efficiency of 18.6% (*J*_sc_: 21.9 mA cm^–2^, *V*_oc_: 1.11 V, FF: 77%), while the DWCNTs-Cz device was less efficient, with a PCE of 17.3% (*J*_sc_: 20.8 mA cm^–2^, *V*_oc_: 1.09 V, FF: 76%). By examination of these results, it is evident that performance increases in sequence from DWCNTs to SWCNTs to CNHs. The lower increase in the performance of DWCNTs may be attributable to their comparatively lower degree of functionalization, as previously mentioned. Upon examination of the two functionalization types (TPA and Cz), it was found that TPA-functionalized devices demonstrated a PCE increase of 13–17%, while the Cz-functionalized devices showed a PCE increase of 4–8% over the control device. The incorporation of TPA-CNMs exhibits the slightly highest *J*_sc_ and FF, with the lowest series resistance (*R*_s_) and highest shunt resistance (*R*_sh_). Conversely, Cz-CNMs provide slightly lower *J*_sc_ and FF, with the highest *R*_s_ and lowest *R*_sh_.

On the other hand, as evident from the best *J*–*V* curves in Figure 4a–c and Table 2, and the statistical analysis in Figures 4d–f and S21 and Table S3, adding nonfunctionalized CNMs (CNHs, SWCNTs, and DWCNTs) in the spiro-OMeTAD layer does not improve device performance. In fact, the pristines increase *R*_s_ and decrease *R*_sh_, resulting in a decrease in FF, *J*_sc_, *V*_oc_, and overall efficiency. These observations highlight the crucial necessity of functionalization in enhancing the performance of PSCs. Since devices incorporating functionalized CNMs yield the highest PCEs, whereas pristine CNMs have limited impact, the subsequent sections focus solely on devices containing p-type functionalized CNMs and exclude those containing pristine ones.

The increase in *J*_sc_ and FF values, as well as the variations in parasitic resistance (*R*_s_ and *R*_sh_) present in the functionalized CNHs are explained below and in the Section 2, respectively. To further analyze the variations in *J*_sc_ in PSCs with functionalized CNMs in the HTL, external quantum efficiency (EQE) measurements were performed. Table 3 and Figure S22 present the calculated *J*_sc_ data obtained by integrating the EQE spectra, which are in agreement with those from the *J*–*V* curves. This confirms that the addition of p-type functionalized CNMs in the spiro-OMeTAD layer improves the *J*_sc_, with both CNHs (CNHs-TPA and CNHs-Cz) exhibiting the highest improvement and TPA-functionalized CNMs having a greater impact on enhancing *J*_sc_ than Cz derivatives.

**Table 3:** Comparison of Short-Circuit Current Density (*J*_sc_) from *J*–*V* Curves and EQE Measurements and Hole Mobility (μ_h_) of the Hole Transport Layers (HTLs) with and without Carbon Nanomaterials (CNMs)

HTL	*J*_sc_ (mA cm^–2^)^a^	*J*_sc_ (mA cm^–2^)^b^	*μ*_h_ (10^–3^ cm^2^ V^–1^ s^–1^)^c^
w/o CNMs	20.5	20.4	4.10
CNHs-Cz	21.5	21.2	6.91
CNHs-TPA	22.0	22.1	7.32
SWCNTs-Cz	21.1	20.9	7.07
SWCNTs-TPA	21.9	21.8	7.69
DWCNTs-Cz	20.8	20.7	6.39
DWCNTs-TPA	21.9	21.7	7.22

a From the *J*–*V* curves.

b From EQE measurements.

c From SCLC method.

The hole mobility (μ_h_) of the HTLs was also analyzed to understand the improvement in current with the addition of functionalized CNMs. The hole mobility was calculated by the space-charge limited current (SCLC) method, fabricating hole-only devices with the structure ITO/PEDOT/PSS/HTL/Au and by fitting with the Mott–Gurney law to the *J*–*V* curves in the dark (Figure S23). The experimental conditions for device preparation are provided in Section 4, and the calculation details are described in the Supporting Information, and the hole mobility values are collected in Table 3. The hole mobility value of spiro-OMeTAD without CNMs was found to be 4.10 × 10^–3^ cm^2^ V^–1^ s^–1^, which agrees with the literature.^60^ The hole mobility was increased to 7.32 × 10^–3^ cm^2^ V^–1^ s^–1^ with the addition of CNHs-TPA, to 7.69 × 10^–3^ cm^2^ V^–1^ s^–1^ with SWCNTs-TPA, and to 7.22 × 10^–3^ cm^2^ V^–1^ s^–1^ with DWCNTs-TPA. Adding CNHs-Cz produced a hole mobility of 6.91 × 10^–3^ cm^2^ V^–1^ s^–1^, while doping with SWCNTs-Cz yielded 7.07 × 10^–3^ cm^2^ V^–1^ s^–1^, and doping with DWCNTs-Cz resulted in a hole mobility of 6.39 × 10^–3^ cm^2^ V^–1^ s^–1^. These results reveal that the addition of all functionalized CNMs to the spiro-OMeTAD increases hole mobility, and those functionalized with TPA show higher charge transport values compared with analogues with Cz. The increase of the charge mobility of TPA-functionalized-doped HTLs could drive the enhancement in hole transport from the perovskite material to the anode, and a decrease in charge recombination at MAPI/HTL interface, which contributes to both increase in *J*_sc_ and FF.^61^ Additionally, DWCNTs derivatives showed the lowest improvement in hole mobility, which is also consistent with the photovoltaic results. The hole mobility values of the SWCNTs derivatives exhibit the highest values, followed by those obtained with the CNHs derivatives. While the hole mobility properties of the HTL directly impact the device efficiency, it is important to note that they are not the only influencing factors for the photovoltaic parameters.^62−64^ It is expected that CNHs-based devices, due to their superior efficiency, also demonstrate superiority in other properties. Consequently, the forthcoming analysis will enclose the evaluation of the charge-transfer process at the perovskite/HTL interface, the effect on the energy levels of the spiro-OMeTAD, and the morphological characterization of the target layers.

**Figure 5:**
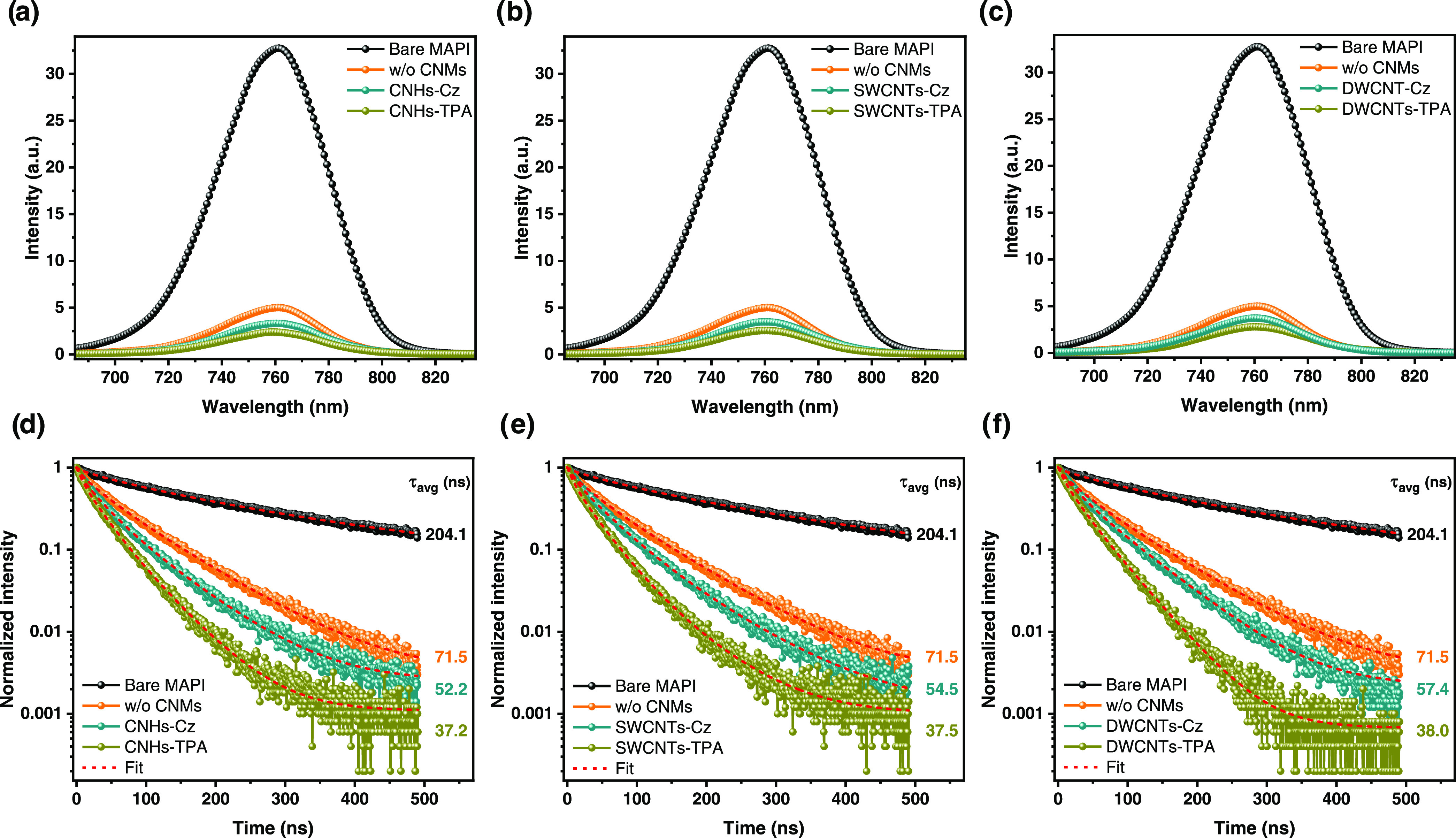
(a–c) Steady-state photoluminescence (PL) spectra and (d–f) time-resolved photoluminescence (TRPL) lifetime curves of MAPI/HTLs substrates with CNHs (a, d), SWCNTs (b, e), and DWCNTs derivatives (c, f) in the hole transport layer (HTL). Orange data represents the HTL without CNMs (w/o CNMs), with carbazole (Cz) derivatives in cyan, and with triphenylamine (TPA) derivatives in olive color. The average lifetimes of the photoluminescence decay are included on the right side of each graph (λ_exc_ = 635 nm).

The charge extraction process at the MAPI/HTL interface was investigated by using both steady-state photoluminescence (PL) and time-resolved photoluminescence (TRPL) measurements. The fabrication process for the substrates and calculations details are described in Section 4^4^. As shown by Figure 5a–c, the PL intensity of the MAPI layer was quenched by all of the spiro-OMeTAD-based films. Notably, the PL intensity reduction was greater in the presence of functionalized CNMs. The HTLs with TPA-functionalized CNMs exhibited a more significant PL reduction compared to those with Cz homologues, indicating that the addition of TPA-functionalized CNMs leads to a more significant improvement in the hole extraction from the perovskite layer. These results were further supported by TRPL measurements shown in Figure 5d–f. The carrier lifetimes were determined by fitting the TRPL curves to a double-exponential decay model, as presented in Table S4. The average lifetime of the photoluminescence decay for the HTL with the control spiro-OMeTAD was 71.5 ns, and for spiro-OMeTAD with CNHs-TPA, SWCNTs-TPA, DWCNTs-TPA, CNHs-Cz, SWCNTs-Cz, and DWCNTs-Cz were 37.2, 37.5, 38.0, 52.2, 54.5, and 57.4 ns, respectively. The faster decay observed on MAPI/spiro-OMeTAD with functionalized CNMs devices indicates a more efficient hole transfer from the perovskite valence band to the HOMO level of the HTL than that on the system with CNMs-free spiro-OMeTAD film. Besides, the PL lifetime of the MAPI/spiro-OMeTAD with TPA-CNMs samples is shorter than that with Cz-CNMs, which indicates faster charge extraction by using TPA-CNMs. Additionally, superior charge extraction properties using CNHs derivatives are evidenced by the fastest decay time values.

UV–vis absorption spectroscopy and cyclic voltammetry were used to investigate the effect of functionalized CNMs on the energy levels of spiro-OMeTAD. The sample fabrication process is described in Section 4^4^. Table S5 summarizes the absorption onset wavelengths (λ_onset_), optical band gaps (Δ*E*), onset potentials of the first oxidation step (*E*_ox_^1^), and HOMO and LUMO energy levels (*E*_HOMO_ and *E*_LUMO_) data of spiro-OMeTAD with and without CNMs.

**Figure 6:**
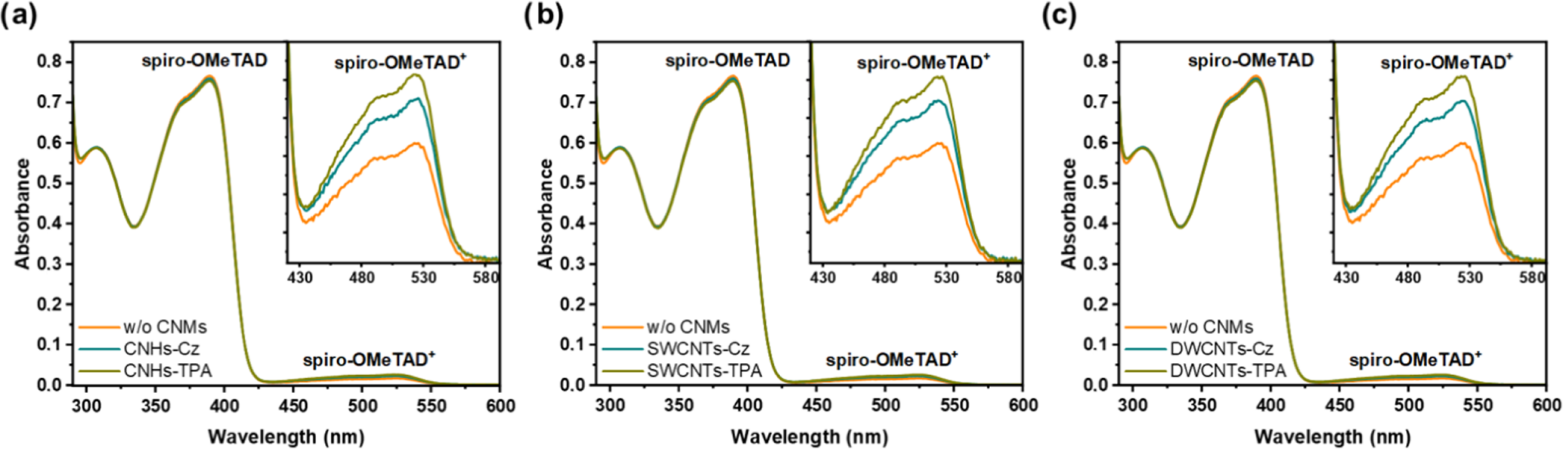
UV–vis absorption spectra of spiro-OMeTAD with (a) CNH derivatives, (b) SWCNT derivatives, and (c) DWCNT derivatives. The spectrum of spiro-OMeTAD without CNMs (w/o CNMs) is shown in orange, with carbazole (Cz) derivatives in cyan, and with triphenylamine (TPA) derivatives in olive color.

Figure 6 displays the absorption spectra of the spiro-OMeTAD-based solutions. Upon the addition of functionalized CNMs, the maximum absorption at 390 nm slightly decreases, while the absorption intensity of the bands between 430 and 580 nm increases. The absorption at 390 nm corresponds to the π–π* transition from the ground state of spiro-OMeTAD, and the bands between 430 and 580 nm have been identified as the absorption of oxidized spiro-OMeTAD (spiro-OMeTAD^+^).^65^ The obtained results indicate that the addition of functionalized CNMs promotes the *p*-type doping of the remaining undoped spiro-OMeTAD, likely due to the CNMs’ ability to act as strong oxidizing agents for triarylamine derivatives such as spiro-OMeTAD.^66^ As evidenced by the Raman measurement, the functionalization enhanced the oxidation capacity of the carbon nanomaterials (CNMs). In addition, the UV–vis spectra show that the TPA-functionalized CNMs exhibited a more pronounced p-type doping effect compared to their Cz counterparts, as evidenced by the higher proportion of spiro-OMeTAD^+^. These results are also consistent with the previously discussed observations of the enhanced hole mobility of spiro-OMeTAD in the presence of TPA-functionalized CNMs.

On the other hand, the energy band gap of spiro-OMeTAD increases from 2.98 eV to 3.00–3.04 eV with the addition of functionalized CNMs (see Table S5), suggesting that the doping of functionalized CNMs affects the energy levels of spiro-OMeTAD.

The HOMO energy level analysis of spiro-OMeTAD with and without CNMs was performed using the cyclic voltammograms illustrated in Figure S24 of the Supporting Information. The CNMs-free spiro-OMeTAD showed a first oxidation potential (*E*_ox_^1^) of −0.045 V. When CNHs-TPA, SWCNTs-TPA, DWCNTs-TPA, CNHs-Cz, SWCNTs-Cz, and DWCNTs-Cz were added, the *E*_ox_^1^ values were +0.024, + 0.011, + 0.001, −0.010, −0.012, and −0.019 V, respectively. These results indicate that the oxidation potential of spiro-OMeTAD is shifted to more positive values in the presence of functionalized CNMs, with TPA derivatives causing a higher shift in comparison to their Cz homologues. The observed anodic shift indicates that more energy is required to initiate the oxidation reaction, likely because the concentration of undoped spiro-OMeTAD decreases upon addition of CNMs. Consequently, a higher potential is needed by the electrochemical system to reach the outer electrons of the unoxidized analyte, which are still present but in a reduced proportion. The UV–vis absorption spectra support these results; the functionalized CNMs promote the formation of oxidized spiro-OMeTAD while reducing the concentration of the remaining undoped spiro-OMeTAD.

**Figure 7:**
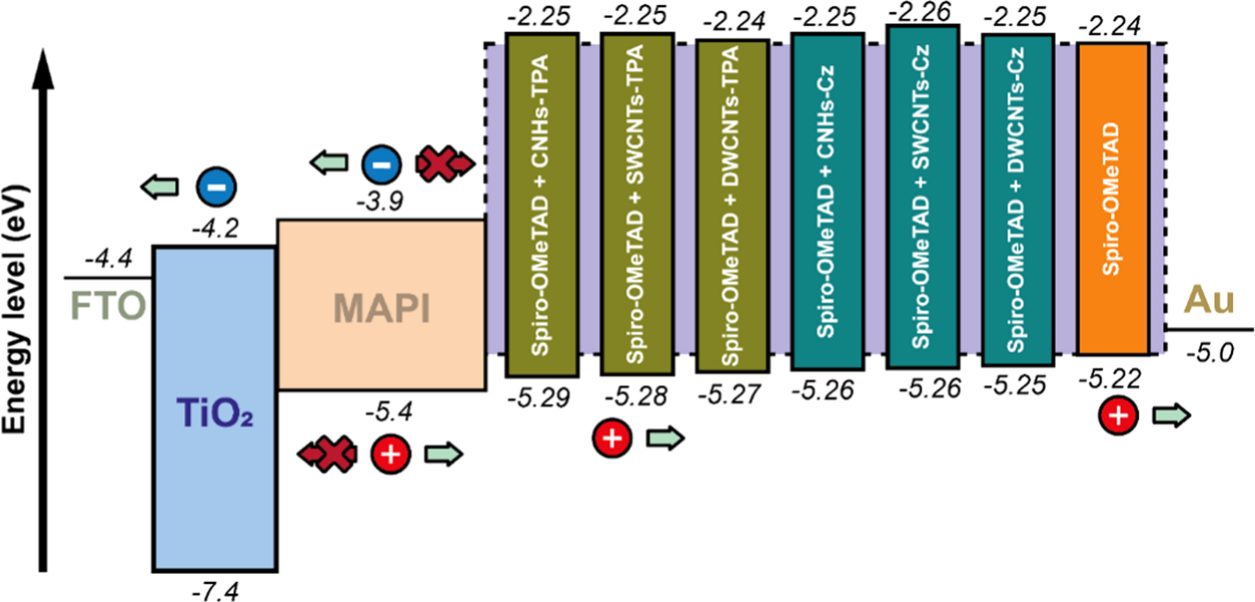
Energy levels for perovskite solar cells (PSCs) using spiro-OMeTAD with and without functionalized CNMs.

The HOMO energy levels of spiro-OMeTAD with and without CNMs were estimated using the *E*_ox_^1^ values and the equation *E*_HOMO_ = −*e*(*E*_ox_^1^ + 5.27). The results are shown in Figure 7, along with the energy levels of the other components in the PSCs. The HOMO energy level of CNMs-free spiro-OMeTAD was found to be −5.22 eV, consistent with previous literature reports.^67^ With CNHs-TPA, SWCNTs-TPA, DWCNTs-TPA, CNHs-Cz, SWCNTs-Cz, and DWCNTs-Cz, the HOMO energy levels were −5.29, −5.28, −5.27, −5.26, −5.26, and −5.25 eV, respectively. The anodic shift in the voltammograms caused by the presence of CNMs results in deeper HOMO energy levels, improving the alignment with the valence band of MAPI (−5.4 eV). Compared to Cz-functionalized CNMs, the HOMO level of spiro-OMeTAD with TPA-functionalized CNMs exhibits a more suitable energetic alignment with the valence band of MAPI, which explains the enhanced hole transfer process observed in the photoluminescence studies and the resulting increase in *J*_sc_ and FF in the photovoltaic parameters for PSCs.

Based on the results obtained from the voltammograms, it is evident that among the types of functionalized CNMs used (functionalized CNHs, SWCNTs, and DWCNTs), CNH derivatives show a superior effect in reducing the HOMO energy level, indicating that CNHs are more efficient for doping spiro-OMeTAD. Regarding the type of functionalization (TPA and Cz), it is understood that the TPA and Cz moieties are not oxidizing agents as strong as the CNMs,^66^ and thus both are unable to induce the oxidation of spiro-OMeTAD on their own. Therefore, the increase in the concentration of spiro-OMeTAD^+^ and the downshift of the HOMO energy levels are a consequence of the chemically functionalized set of CNMs with TPA and Cz, as demonstrated by the Raman measurements previously discussed. The UV spectra and voltammograms show that CNMs-TPA turns out to be the most effective in promoting spiro-OMeTAD oxidation. The superiority of the TPA derivatives is probably due to their greater ease of oxidation compared to the Cz moiety. Previous literature reports indicate that the oxidation potential of Cz is about +0.3 V more positive than that of the corresponding TPA.^68^

The morphology of the spiro-OMeTAD-based HTLs was analyzed by field emission scanning electron microscopy (FE-SEM) using substrates based on FTO/c-TiO_2_/m-TiO_2_/MAPI/HTL with an HTL based on spiro-OMeTAD with and without functionalized CNM. The top-view images presented in Figure S25 demonstrate that the morphology of the spiro-OMeTAD layer was not significantly altered by the addition of TPA-functionalized CNMs. However, when Cz-functionalized CNMs were added, the layers exhibited variations in black–white tonalities. Figure S25 highlights the darker-toned regions. This observation suggests that the decreased FF of the PSCs with Cz-CNMs is likely due to the lower homogeneity, which results in a poorer contact interface. The HTL layer has an appropriate thickness ranging from 200 to 212 nm, as can be seen in the cross-sectional image in Figure S26.

**Figure 8:**
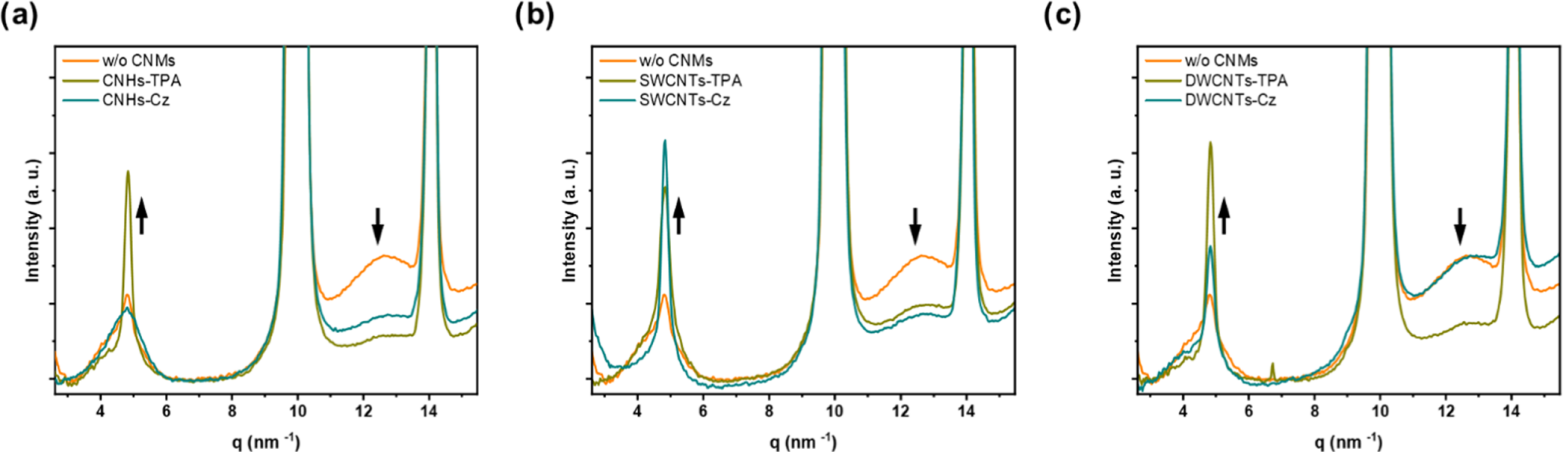
Integrated profiles of GIWAXS patterns from FTO/c-TiO_2_/m-TiO_2_/MAPI/spiro-OMeTAD-based HTM film with (a) CNHs derivative, (b) SWCNTs derivatives, and (c) DWCNTs derivatives. The spectrum of spiro-OMeTAD without CNMs (w/o CNMs) is shown in orange, with carbazole (Cz) derivatives in cyan, and with triphenylamine (TPA) derivatives in olive color. The arrows indicate changes in the relative intensity between scattering features from spiro-OMeTAD. The peaks observed at 10 and 14 nm^–1^ correspond to the MAPI layer.

To analyze the impact of CNMs on the structural rearrangement of spiro-OMeTAD, X-ray diffraction (XRD) measurements were carried out on top of substrates FTO/c-TiO_2_/m-TiO_2_/MAPI/spiro-OMeTAD-based HTLs. The XRD patterns in Figure S27 reveal that the diffractograms are very similar to each other, showing peaks at 2θ values of 14.20, 20.08, 23.51, 24.55, 28.58, 35.1, 40.8, and 43.0°, which are attributed to the (110), (112), (211), (202), (220), (310), (312), (224, 040), and (314, 330) planes of the tetragonal perovskite structure.^69^ FTO diffraction peaks were observed at angles of 26, 34, and 38°. The analysis of the data shows that there are no significant variations in regard to the presence of CNMs in the spiro-OMeTAD layer, most likely due to the low sensitivity of the technique toward spiro-OMeTAD samples with less crystallinity compared to perovskite and FTO. To be able to appreciate the changes in the spiro-OMeTAD layers, additional investigations using GIWAXS were performed on similar substrates. Two reflections associated with spiro-OMeTAD are detected on the GIWAXS patterns at 4.8 and 12.5 nm^–1^ (Figure S28), which are consistent with previous works.^70,71^ The integrated profiles of the GIWAXS patterns are illustrated in Figure 8. The reflection at *q_z_* = 4.8 nm^–1^ is oriented vertically, while the reflection at *q_z_* = 12.5 nm^–1^ is isotropic and likely associated with the intermolecular π–π stacking between conjugated planes.^72^ After the incorporation of functionalized CNMs, the low-*q* peak exhibited a systematic increase in relative intensity, indicating an improvement in the vertical order of the spiro-OMeTAD layer. The systematic ordering of spiro-OMeTAD induced by the CNMs is consistent with improved layer morphology, better contact interface, more efficient charge extraction, and, consequently, the observed enhancement in device performance.

Regarding parasitic resistances (*R*_s_ and *R*_sh_), these represent the photocurrent losses through an applied voltage.^73^ The decrease in the *R*_s_ value achieved by incorporating functionalized CNMs (Figure 4 and Table 2) is directly associated with improvements in the hole transfer properties in the spiro-OMeTAD layer and the quality of the interfacial contact between the perovskite and the HTL (confirmed through EQE, PL, hole mobility measurements, FE-SEM images, and GIWAX analysis). Previous studies have already established the correlation between *R*_s_ and the charge-transfer properties at the perovskite interface and the contact interface state.^74^ On the other hand, the influence of functionalized CNMs on the increase in the HOMO energy level of spiro-OMeTAD directly impacts *R*_sh_. As shown in Figure 4 and Table 2, the *R*_sh_ values are higher when functionalized CNMs are present. Concerning the meaning of *R*_sh_, it has been proven that this parasitic resistance represents the leakage current, which is associated with the value of *V*_oc_.^75,76^ In other words, it is linked to the potential difference between the LUMO energy level of the ETL and the HOMO energy level of the HTL. As the HOMO of spiro-OMeTAD increases with the addition of functionalized CNMs, the LUMO–HOMO band gap widens, resulting in an increase in *V*_oc_ and a decrease in leakage current, both of which are represented by the higher value of *R*_sh_. Therefore, the inclusion of functionalized CNMs results in a reduction in *R*_s_ and an increase in *R*_sh_. Consequently, FF is increased, leading to higher values of PCE. Furthermore, improved hole transport properties contribute to an increase in *J*_sc_, further enhancing the overall PCE.

It is crucial to emphasize that *R*_s_ and *R*_sh_ encompass various processes related to photocurrent losses.^77^ This study thoroughly examines multiple factors contributing to the reduction of these losses (better charge transport in the interface, improved hole mobility in the HTL, and enhanced morphology). Upon meticulous examination of these parameters, it becomes evident that among the various types of functionalized CNMs utilized, derivatives of CNHs exhibit highly favorable characteristics. Specifically, devices based on CNHs demonstrate the lowest *R*_s_ values and the highest *R*_sh_ values, indicating that CNHs effectively minimize these photocurrent losses.

### 2.3 Stability Experiments of the Perovskite Solar Cells

**Figure 9:**
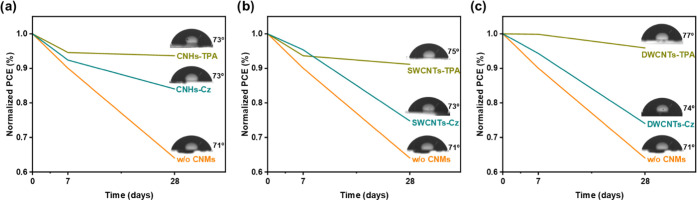
Long-term stability of the device containing spiro-OMeTAD layer with (a) CNHs, (b) SWCNTs, and (c) DWCNTs. The device based on spiro-OMeTAD without CNMs (w/o CNMs) is shown in orange, with carbazole (Cz) derivatives in cyan, and with triphenylamine (TPA) derivatives in olive color (active area for PSCs 0.16 cm^2^, AM 1.5G illumination, scan rate 100 mV cm^–2^). The water contact angles for each HTL are illustrated on the right side of each graph.

The long-term stability tests were conducted by storing the samples in the dark for 28 days at a temperature of 20 ± 3 °C and a relative humidity of 33 ± 2%. The PCE values were measured at the beginning of the experiment (0 days), after 1 week (7 days), and 4 weeks later (28 days), as presented in Figure 9.

The results indicate that incorporating CNMs in PSCs significantly improves device stability, as compared to the reference device without CNMs, which experienced a decline in initial PCE to 65% at the end of the test. All devices with TPA-functionalized CNMs retained more than 90% of their initial performance after 28 days, while Cz-functionalized nanoforms maintained their performance at around 75–85%. It is worth noting that in the case of Cz-CNMs, the CNHs had a more pronounced effect on enhancing device stability.

The water contact angles of FTO/c-TiO_2_/m-TiO_2_/MAPI/HTL-based samples were measured to understand the origin of the enhanced stability of the solar cells due to the addition of CNMs. The images inset to the stability plots in Figure 9 show a higher contact angle on samples with CMNs, suggesting that the improved device stability is related to the enhancement of the hydrophobicity of the HTL. Comparing both types of functionalization (TPA and Cz), adding TPA-CNMs increases the hydrophobicity of the spiro-OMeTAD layer, likely due to TPA having a less planar structure compared to Cz, which makes it less susceptible to interacting with water.^78^ Furthermore, the enhancement in holes transport achieved through the doping of HTLs with TPA-functionalized CNMs, as discussed earlier, circumvents charge leakage across the MAPI/spiro-OMeTAD interface, which is also a factor contributing to the instability of PSCs.^79^

## 3 Conclusions

In summary, six new functionalized p-type carbon CNMs, namely, CNHs-TPA, CNHs-Cz, SWCNTs-Cz, SWCNTs-TPA, DWCNTs-TPA, and DWCNTs-Cz, were successfully synthesized using [2 + 1] cycloaddition reactions with azides based on triphenylamine (TPA) and 9-phenyl carbazole (Cz). The functionalization degree was determined through TGA, revealing that the DWCNTs, having lower reactivity, exhibited the lowest degree of functionalization. The preservation of the π-conjugation integrity was confirmed by different spectroscopy techniques, including UV–vis-NIR and Raman spectroscopies, PLE, and XPS. The impact of synthesized CNMs on the performance of regular mesoporous PSCs was evaluated by their incorporation in the doped spiro-OMeTAD layer. The importance of the functionalization of carbon CNMs for their application in PSCs was evidenced after observing that, under the experimental conditions used, none of the pristine CNMs reveals improvement in the performance of the devices. The *J*–*V* curve results show that functionalized CNMs improve the PCE mainly by increasing *J*_sc_ and FF. The improvement of *J*_sc_ also was confirmed by EQE measurements. Compared to the control devices with a PCE of 16.6%, the CNHs-TPA, CNHs-Cz, SWCNTs-TPA, SWCNTs-Cz, DWCNTs-TPA, and DWCNTs-Cz devices exhibited PCE increases of 17% (from 16.6 to 19.4%), 8% (from 16.6 to 17.9%), 15% (from 16.6 to 19.0%), 7% (from 16.6 to 17.8%), 12% (from 16.6 to 18.6%), and 4% (from 16.6 to 17.3%), respectively. These results reflect that PSCs with CNH derivatives obtained slightly higher increases in PCE compared to those with SWCNTs and DWCNTs, and the TPA-functionalized derivatives yielded better results than their Cz-functionalized counterparts. The enhancements observed in the photovoltaic parameters were attributed to several factors, which were proved by various techniques. The SCLC method demonstrated an improvement in spiro-OMeTAD hole mobility. The UV–vis absorption spectra showed an increase in additional p-type doping, and better alignment of energy levels between the spiro-OMeTAD and MAPI layers was demonstrated through cyclic voltammetry. Further evidence of the improvement in contact interfaces was provided by PL measurements, while FE-SEM images and GIWAXS analyses confirmed an enhancement of the morphology of the HTLs. In addition, functionalized CNMs improved device stability by enhancing the hole transport and increasing the hydrophobicity of HTLs. The devices based on TPA-functionalized CNMs maintained over 90% of their performance, while those with Cz-functionalized CNMs retained between 75 and 85% of their initial value. Notably, CNHs-Cz exhibited the highest stability among the Cz-functionalized CNMs.

Ultimately, this study shows that incorporating functionalized carbon CNMs into PSCs is an effective approach for increasing their efficiency and improving their stability. It also reveals that CNMs require functionalization to enhance performance, as pristine materials do not show significant changes in photovoltaic parameters. Carbon nanohorns (CNHs) show better improvements than nanotubes (SWCNTs and DWCNTs), and the type of functionalization is crucial. Incorporating all TPA derivatives results in more effective improvements than those of their Cz counterparts. This research expands the application of CNHs, first reported here for the PSCs field, and is expected to serve as a guide for inspiring future research on other organic functionalization.

## 4 Experimental Section

### 4.1 Synthetic Procedure

#### 4.1.1 Materials

All chemicals were reagent-grade, purchased from commercial suppliers, and used as received. Column chromatography was performed on Merck silica gel 60 (ASTM 230–400 mesh). Pristine single-walled carbon nanohorns (CNHs) used were produced by CO_2_ laser ablation of graphite in the absence of any metal catalyst under an argon atmosphere (760 Torr) at room temperature; the purity of the CNHs was as high as >90% for less amorphous carbons. Purified HiPco single-walled carbon nanotubes (SWCNTs) were purchased from NanoIntegris (purified grade, length = 100–1000 nm, diameter = 0.8–1.2 nm, < 15% remaining iron particles). In the text, HiPco SWCNTs are referred to simply as SWCNTs. Pristine double-walled carbon nanotubes (DWCNTs) were acquired from XinNano Materials, Inc. (Cat No.: XNM-UP-11050, purity >98%, the diameters of the outer tubes and the inner tubes were found to be 1.00–1.81 and 0.88 nm, respectively). Pristine carbon nanomaterials were used without further purification.

#### 4.1.2 General Procedure for the Synthesis of Functionalized Carbon Nanomaterials (CNMs)

(See Scheme 2): Pristine CNMs (1 mg) were added to *N*-methyl-2-pyrrolidone (NMP, 3.3 mL) and sonicated at 25 °C for 1 h in the case of CNHs and SCWNTs, and for 7 h in the case of DWCNTs. After that, the suspensions were stirred and purged with argon for 15 min at room temperature. The corresponding azide (5 mg of azide per 1 mg of CNMs) dissolved in the minimum volume of NMP was added dropwise to the mixture. Thereafter, the suspensions were stirred at room temperature for 24 h for functionalized CNHs and SWCNTs, and for 48 h for functionalized DWCNTs. The product was filtered through a 0.45 μm pore size OMNIPORE PTFE membrane, collected, and washed with NMP, dichloromethane, and methanol several times until the filtrate was colorless. Finally, the black materials were dried overnight to afford the desired functionalized CNMs.

#### 4.1.3 Characterization

The sonication of CNM suspension was carried out using an Elmasonic P 300 H sonicator bath operating at 37 kHz. Nuclear magnetic resonance (NMR) spectra were recorded on a Bruker Avance 400 MHz UltrashieldTM spectrometer (^1^H: 400 MHz; ^13^C: 100 MHz) at 298 K, unless otherwise stated, using partially deuterated solvents as internal standards. Coupling constants (*J*) are indicated in Hz, and chemical shifts (δ) are reported in ppm. Attenuated total reflection Fourier transform infrared (ATR-FTIR) spectra were recorded using an Avatar 370 spectrophotometer within a spectral range of 400 to 4000 cm^–1^. Thermogravimetric analysis-derivative thermogravimetry (TGA-DTG) analysis was performed using a Mettler–Toledo Linea Excellent instrument collected under a nitrogen flow (90 mL min^–1^). Approximately 0.6 mg of the sample was introduced into a platinum crucible that was equilibrated at 40 °C, followed by a ramp of 10 °C min^–1^ from 40 to 1000 °C. Details for the calculations are provided in the Supporting Information. UV–vis–NIR absorption spectra were recorded at room temperature by using a PerkinElmer Lambda 950 spectrometer. The spectra were recovered in Milli-Q-grade water containing 10 wt % of sodium dodecylbenzene sulfonate (SDBS) as a surfactant. The concentrations of the prepared solutions were adjusted to be the same. Photoluminescence excitation intensity mapping (PLE) was recorded at room temperature on a HORIBA Jobin Yvon Nanolog 4 Spectrofluorometer equipped with a multichannel InGaAs detector. The pristine and the functionalized CNMs spectra were recovered in Milli-Q grade water containing 10 wt % of SDBS as surfactant. Raman spectroscopy measurements were acquired with a Renishaw inVia Reflex Confocal Raman Microscope equipped with a 785 nm laser. Raman spectra were collected on numerous spots on the sample and recorded with a Peltier-cooled CCD camera. The Raman extended spectra are the average of 10 acquisitions recorded from 130 to 3000 cm^–1^, 785 nm laser wavelength, 5% laser power, and exposure time 10 s. The Raman average spectra are the average of 400 individual acquisitions taken over the sample, recorded from 1200 to 1700 cm^–1^, 785 nm laser wavelength, 5% laser power, and exposure time 1 s. Each sample was deposited as a powder on a glass slide and was measured in multiple regions. All Raman spectra were normalized at the G-band peak intensity. The intensity ratio ID/IG was obtained by taking the peak intensities following any baseline corrections. The data were collected and analyzed with Renishaw Wire and Origin software. The X-ray photoelectron spectroscopy (XPS) analysis was conducted using a SPECS system (Berlin, Germany) equipped with a Phoibos 150 1D-DLD analyzer and monochromatic Al Kαα radiation source (1486.7 eV). Initially, a wide scan was performed with a step energy of 1 eV, dwell time of 0.1 s, and pass energy of 80 eV. Subsequently, a detailed analysis of the detected elements was carried out using a scan step energy of 0.08 eV, dwell time of 0.1 s, pass energy of 30 eV, and electron exit angle of 90°. Prior to analysis, the spectrometer was calibrated with Ag (Ag 3d_5/2_, 368.26 eV). The obtained spectra were analyzed using CasaXPS 2.3.16 software, which employs Gauss–Lorentzian contributions for modeling after background subtraction (Shirley). The concentrations of elements were calculated by adjusting the values with relative atomic sensitivity factors (Scofield).

### 4.2 Perovskite Solar Cells

#### 4.2.1 Materials

The materials used in the photovoltaic study were obtained from commercial suppliers in high purity and used without further purification. Methylammonium iodide (MAI, > 99.9%, Greatcell Solar Materials), lead(II) iodide (PbI_2_, 99.9%, TCI chemicals), titanium chloride (TiCl_4_, > 99.0% Sigma-Aldrich), TiO_2_ paste (30 NR-D titania paste, Greatcell Solar Materials), 2,2′,7,7′-tetrakis[*N*,*N*-di(4-methoxyphenyl)amino]-9,9′-spirobifluorene (spiro-OMeTAD, >99.0%, Shenzhen Furui Technology Co.,Ltd.), bis(trifluoromethane)sulfonimide lithium salt (Li-TFSI, 99.9%, Sigma-Aldrich), tris(2-(1*H*-pyrazol-1-yl)-4-*tert*-butylpyridine)cobalt(II)di[bis(trifluoromethane)sulfonimide] (FK209, 98%, Sigma-Aldrich), 4-*tert*-butylpyridine (*t*-BP, 98%, Sigma-Aldrich), poly(3,4-ethylenedioxythiophene) poly(3,4-ethylenedioxythiophene) and polystyrenesulfonate dispersion (PEDOT/PSS dispersion, Heraeus Clevios P VP AI 4083), ethanol for cleaning (EtOH, technical grade, Scharlab), isopropanol for cleaning (IPA, technical grade, Scharlab), dimethylformamide (DMF extra dry, Acros Organics), dimethyl sulfoxide (DMSO, extra dry, Acros Organics), ethanol for precursor solutions (EtOH, dry SeccoSolv, Merk), chlorobenzene (extra dry, Acros Organics), and acetonitrile (extra dry, Acros Organics). Gold wire (Au, 99.99%, diameter 0.5 mm, Kurt J. Lesker). FTO glass substrates (Nippon sheet glass, 1.4 cm × 2.5 cm, 13 Ω sq^–1^) were purchased from Xop Física S. L. Prepatterned ITO glass substrates (1.5 cm × 1.5 cm, 15 Ω sq^–1^) were acquired from Xinyan Technology Ltd.

#### 4.2.2 Device Fabrication

The compact TiO_2_ (c-TiO_2_) precursor solution was composed of 2 M TiCl_4_ in deionized water. The precursor solution for mesoporous TiO_2_ (m-TiO_2_) was prepared by dissolving 150 mg of TiO_2_ paste in 1 mL of ethanol. The Li-TFSI precursor solution for doping the TiO_2_ layer was made by dissolving 10 mg of the precursor in 1 mL of acetonitrile. Spiro-OMeTAD was doped with Li-TFSI and FK209 solutions, which had a concentration of 1.8 and 0.25 M in acetonitrile, respectively. A 1.4 M MAPI solution was prepared by mixing 646.70 mg of PbI_2_, 223.00 mg of MAI, and 1 mL of a mixture of 7:3 DMF and DMSO. The HTL precursor solutions were prepared starting from stock solutions of 1 mg/mL for SWCNTs derivatives (pristine SWNTs, SWNTs-TPA, SWNTs-Cz) and CNHs derivatives (pristine NHs, NHs-TPA, NHs-Cz), and 0.5 mg/mL for DWCNTs derivatives (pristine DWNTs, DWNTs-TPA, and DWNTs-Cz). These stock solutions were prepared by ultrasonic bath-assisted dispersion using an Elmasonic S 40 H ultrasonic bath (Elma Schmidbauer GmbH). The dispersion process was carried out in chlorobenzene as the solvent, maintaining a temperature of 25 °C, and under ultrasonic treatment for a duration of 2 h (for SWCNTs and CNHs derivatives) and 8 h (for DWCNTs derivatives). The stock solution results were then filtered by using a hydrophobic PTFE membrane with a pore size of 0.45 μm. Spiro-OMeTAD was dissolved in the resulting dispersions at a concentration of 70 mM. A reference solution was also prepared by using chlorobenzene without nanomaterials. All spiro-OMeTAD-based solutions (the reference one and the nine solutions with the CNMs derivatives) were doped with *t*-BP (3.3 equiv), Li-TFSI (0.5 equiv), and FK209 (0.05 equiv) and kept under vigorous magnetic stirring until use. The precursor solutions for the HTLs were prepared by dispersing the nanomaterials (pristine NHs, NHs-TPA, NHs-Cz, pristine SWNTs, SWNTs-TPA, SWNTs-Cz, pristine DWNTs, DWNTs-TPA, and DWNTs-Cz) in chlorobenzene through controlled ultrasonication for 2 h at 25 °C (Elmasonic S 40 H ultrasonic cleaning bath, Elma Schmidbauer GmbH). The dispersions were then filtered by using a hydrophobic PTFE membrane with a pore size of 0.45 μm. This methodology allows for the preparation of dispersions with the highest achievable concentration of CNMs in chlorobenzene. Spiro-OMeTAD was dissolved in the resulting dispersions at a concentration of 70 mM. A reference solution was also prepared using chlorobenzene without nanomaterials. All spiro-OMeTAD-based solutions (the reference one and the nine solutions with the CNMs derivatives) were doped with *t*-BP (3.3 equiv), Li-TFSI (0.5 equiv), and FK209 (0.05 equiv) and kept under vigorous magnetic stirring until use.

After preparation of the precursor solution, the devices were fabricated following the architecture based on FTO/c-TiO_2_/m-TiO_2_/MAPI/HTL/Au. Selective etching of one edge of the FTO glass substrates was performed using a mixture of Zn powder and 3 M HCl in deionized water. Subsequently, the substrates were cleaned using a diluted solution of Hellmanex cleaner in deionized water (Hellmanex III, Sigma-Aldrich), followed by ultrasonication (Elmasonic S 40 H ultrasonic cleaning bath, Elma Schmidbauer GmbH) with 2% v/v solution of Hellmanex in deionized water for 15 min. The substrates were then thoroughly rinsed with deionized water and underwent additional ultrasonication with EtOH and IPA for 30 min each. Finally, the substrates were dried under a nitrogen flow and exposed to UV–ozone treatment for 15 min (L2002A3-UK UV–ozone cleaner, Ossila). All layers were prepared through spin coating (WS-650HZ-23NPP/UD2 spin coater, Laurell Technologies). Initially, 100 μL of 2 M TiCl_4_ solution was deposited onto cleaned FTO at a spinning speed of 5000 rpm for 30 s. The substrates were then annealed at 100 °C for 10 min. Upon cooling to room temperature, 100 μL of the TiO_2_ paste solution was deposited at a spinning speed of 4000 rpm for 10 s. The substrates were then subjected to a sintering process by ramping up the temperature to 450 °C (PR5-3T ramp controller, Harry Gestigkeit GmbH with PZ 28-3TD hot plate, Harry Gestigkeit GmbH). Subsequently, 100 μL of 10 mg mL^–1^ Li-TFSI solution was deposited onto the m-TiO_2_ layer at a spinning speed of 3000 rpm for 10 s, followed by a second sintering process using the same heating ramp. After cooling down to 150 °C, the substrates were immediately transferred to a nitrogen-filled glovebox (UNIlab SP, MBraun). Next, 70 μL of MAPI solution was deposited onto the doped-TiO_2_ using a two-step spin-coating process, with speeds of 1000 and 4000 rpm for 10 and 30 s, respectively. During the spinning, 200 μL of chlorobenzene was dropped onto the center of the film 22 s before the end of the procedure. The samples were then annealed at 60 °C for 1 min, followed by annealing at 100 °C for 30 min (2860 SR controller, Präzitherm with PZ 14 ET hot plate, Harry Gestigkeit GmbH). The thickness of m-TiO2/perovskite combination layer was around 550 nm. After cooling, 50 μL of the spiro-OMeTAD-based solution was dynamically deposited at 4000 rpm for 20 s. The substrates were left to rest in a black box with a flow of dry air overnight. To complete the device, an 80 nm thick gold electrode was deposited using thermal evaporation with a shadow mask having an active area of 0.25 cm^2^ (Q150T turbomolecular pumped coater, Quorum Technologies).

#### 4.2.3 Characterization

Current–voltage (*J*–*V*) measurements were conducted under standard conditions of 1 sun intensity (1000 W m^–2^, AM 1.5G, 25 °C) using a Keithley 2460 source meter and a Solixon A-20 solar simulator, a scan rate of 100 mV s^–1^, and preconditioning at short circuit immediately followed by a forward scan. The solar simulator was calibrated with a reference cell based on monocrystalline silicon with a standard quartz window (RR-1002/RQN7622 without filter, Rera Solutions). Tracer IV-Curve measurement software (Rera Solutions) was used to process the photovoltaic parameters. To reduce the influence of scattered light, a black mask with a 0.16 cm^2^ active area was used to cover the devices. The external quantum efficiency (EQE) measurements were recorded using quantum efficiency measurement systems from Lasing, S.A. (IPCE-DC, LS1109-232) and a Newport 2936-R power-meter unit. The data were taken during a wavelength sweep ranging from 300 to 1000 nm. The hole mobility (μ_h_) of the hole transport layers (HTLs) was determined by using the space-charge limited current (SCLC) method in hole-only devices with ITO/PEDOT/PSS/HTL/Au structures. ITO glass substrates were sequentially cleaned by ultrasonication (3000617 ultrasonic cleaning bath, J.P. Selecta) with EtOH and IPA for 15 min, dried with nitrogen flow, and treated with UV–ozone for 20 min (T10 × 10 UV–ozone cleaner, UVOCS Inc.). PEDOT/PSS dispersion was spin-coated onto the ITO substrate at 4000 rpm for 40 s and annealed to 150 °C for 10 min to create a 40 nm thick film (XP-1 profilometer, Ambios Technology). The HTLs were prepared using the same process as for PSCs, resulting in a 170 nm thick film. Finally, 80 nm of gold was thermally evaporated onto the HTL by using a shadow mask with an active area of 0.09 cm^2^. Details for the calculations are provided in the Supporting Information. Measurements of steady-state photoluminescence (PL) and time-resolved photoluminescence (TRPL) were conducted using a fluorescence lifetime spectrometer (LifeSpec II, Edinburgh Instruments) that integrated a photomultiplier tube (PMT) detector and a double subtractive monochromator. The system employed a picosecond pulsed diode laser (EPL-635, Edinburgh Instruments) with a wavelength of 635 ± 10 nm. All measurements were performed under ambient conditions and with fresh samples. To assess the MAPI response, the glass/MAPI structure was employed, whereas the glass/MAPI/HTL structures were utilized to evaluate the interaction between MAPI and the HTLs. The preparation processes for both the MAPI layer and HTLs were identical to those employed for PSCs. Details for the calculations are provided in the Supporting Information. UV–vis absorption spectra were obtained by using a PerkinElmer Lambda 950 spectrometer. The spectra were recorded at room temperature from solutions containing 0.01 mM spiro-OMeTAD with and without nanomaterials in chlorobenzene. These solutions were prepared from the 70 mM precursor solutions used for the PSCs fabrication. Cyclic voltammetry was carried out on a Princeton Applied Research Parstat 2273 with a three-electrode electrochemical cell consisting of a glassy carbon disk working electrode (Ø = 3 mm, CH Instruments, CHI104), a platinum wire counter electrode (Ø = 0.5 mm, CH Instruments, CHI115), and a silver wire pseudoreference electrode (Ø = 0.5 mm, CH Instruments, CHI112). To prepare the solutions for the experiments, 1 mM spiro-OMeTAD with and without nanomaterials was obtained from the 70 mM spiro-OMeTAD precursor solutions used for PSCs fabrication. A mixture of 0.1 M tetrabutylammonium hexafluorophosphate (Bu_4_NPF_6_, 98%, Sigma-Aldrich) in a 4:1 mixture of chlorobenzene and acetonitrile was used to achieve the required concentration. An internal reference of 0.5 mM decamethylferrocene (DMFc/DMFc^+^ = −0.56 V) was used in all experiments, which were run at a scan rate of 100 mV s^–1^. The morphologies of the substrates were analyzed with an ULTRA plus ZEISS field emission scanning electron microscope. The samples analyzed were based on FTO/c-TiO_2_/m-TiO_2_/MAPI/HTLs structures. All layers were prepared by using the same procedures as those described for PSCs fabrication. X-ray diffraction (XRD) analysis was performed using a Bruker AXS-D8 Advance X-ray diffractometer with CuKa radiation. The samples consisted of FTO/c-TiO_2_/m-TiO_2_/MAPI/HTLs structures. All layers were prepared using the same procedures as those described for PSCs fabrication. The samples analyzed for grazing-incidence wide-angle X-ray scattering (GIWAX) were based on FTO/c-TiO_2_/m-TiO_2_/MAPI/HTLs structures and were prepared using the same procedures as those used for PSC fabrication. The GIWAXS patterns were collected at ALBA Synchrotron (Barcelona, Spain) at BL11 (NCD-SWEET). The measurements were taken under ambient conditions, using a beam of 100 μm along the horizontal direction. The energy of the beam was set at 12.4 keV, and the incident angles of 0.2°, with an exposure time of no more than 1 s, to avoid degradation. The patterns were recorded using a WAXS LX255-HS detector (Rayonix) positioned 20 cm away from the sample holder. The contact angles were measured using the drop technique and an optical tensiometer (Theta Pulsating Drop, Attension Theta Lite) under ambient conditions. Water droplets of 10 μL were applied, and the contact angle values were averaged over four measurements taken at four different positions for each surface.

## Supporting Information Available

Experimental section, including the synthetic procedure for the synthesis of precursors and more details for the synthesis of functionalized carbon nanomaterials (CNMs); details regarding calculations; and supporting figures and tables for both synthetic and device characterization (PDF)

## Dedication

Dedicated to Prof. Maurizio Prato on the occasion of his retirement.

## References

[ref1] Jena A. K.; Kulkarni A.; Miyasaka T. Halide Perovskite Photovoltaics: Background, Status, and Future Prospects. Chem. Rev. 2019, 119 (5), 3036–3103. 10.1021/acs.chemrev.8b00539. 30821144

[ref2] Yin X.; Zhai J.; Song L.; Du P.; Li N.; Yang Y.; Xiong J.; Ko F. Novel NiO Nanoforest Architecture for Efficient Inverted Mesoporous Perovskite Solar Cells. ACS Appl. Mater. Interfaces 2019, 11 (47), 44308–44314. 10.1021/acsami.9b15820. 31687805

[ref3] Collavini S.; Delgado J. L. Carbon Nanoforms in Perovskite-Based Solar Cells. Adv. Energy Mater. 2017, 7 (10), 1601000 10.1002/aenm.201601000.

[ref4] Habisreutinger S. N.; Leijtens T.; Eperon G. E.; Stranks S. D.; Nicholas R. J.; Snaith H. J. Carbon Nanotube/Polymer Composites as a Highly Stable Hole Collection Layer in Perovskite Solar Cells. Nano Lett. 2014, 14 (10), 5561–5568. 10.1021/nl501982b. 25226226

[ref5] Collavini S.; Amato F.; Cabrera-Espinoza A.; Arcudi F.; Đorđević L.; Kosta I.; Prato M.; Delgado J. L. Efficient and Stable Perovskite Solar Cells Based on Nitrogen-Doped Carbon Nanodots. Energy Technol. 2022, 10 (6), 2101059 10.1002/ente.202101059. PMC9286678 35866062

[ref6] Yadeta T. F.; Huang K.-W.; Imae T.; Tung Y.-L. Enhancement of Perovskite Solar Cells by TiO_2_-Carbon Dot Electron Transport Film Layers. Nanomaterials 2023, 13 (1), 186 10.3390/nano13010186. PMC9823919 36616096

[ref7] Hadadian M.; Smått J.-H.; Correa-Baena J.-P. The Role of Carbon-Based Materials in Enhancing the Stability of Perovskite Solar Cells. Energy Environ. Sci. 2020, 13 (5), 1377–1407. 10.1039/C9EE04030G.

[ref8] Völker S. F.; Vallés-Pelarda M.; Pascual J.; Collavini S.; Ruipérez F.; Zuccatti E.; Hueso L. E.; Tena-Zaera R.; Mora-Seró I.; Delgado J. L. Fullerene-Based Materials as Hole-Transporting/Electron-Blocking Layers: Applications in Perovskite Solar Cells. Chem. - Eur. J. 2018, 24 (34), 8524–8529. 10.1002/chem.201801069. 29570869

[ref9] Yang Y.; Chen H.; Hu C.; Yang S. Polyethyleneimine-Functionalized Carbon Nanotubes as an Interlayer to Bridge Perovskite/Carbon for All Inorganic Carbon-Based Perovskite Solar Cells. J. Mater. Chem. A 2019, 7 (38), 22005–22011. 10.1039/C9TA08177A.

[ref10] Yavari M.; Mazloum-Ardakani M.; Gholipour S.; Marinova N.; Delgado J. L.; Turren-Cruz S.; Domanski K.; Taghavinia N.; Saliba M.; Grätzel M.; Hagfeldt A.; Tress W. Carbon Nanoparticles in High-Performance Perovskite Solar Cells. Adv. Energy Mater. 2018, 8 (12), 1702719 10.1002/aenm.201702719.

[ref11] Collavini S.; Kosta I.; Völker S. F.; Cabanero G.; Grande H. J.; Tena-Zaera R.; Delgado J. L. Efficient Regular Perovskite Solar Cells Based on Pristine [70]Fullerene as Electron-Selective Contact. ChemSusChem 2016, 9 (11), 1263–1270. 10.1002/cssc.201600051. 26991031

[ref12] Pascual J.; Kosta I.; Ngo T. T.; Chuvilin A.; Cabanero G.; Grande H. J.; Barea E. M.; Mora-Seró I.; Delgado J. L.; Tena-Zaera R. Electron Transport Layer-Free Solar Cells Based on Perovskite-Fullerene Blend Films with Enhanced Performance and Stability. ChemSusChem 2016, 9 (18), 2679–2685. 10.1002/cssc.201600940. 27553898

[ref13] Pascual J.; Delgado J. L.; Tena-Zaera R. Physicochemical Phenomena and Application in Solar Cells of Perovskite:Fullerene Films. J. Phys. Chem. Lett. 2018, 9 (11), 2893–2902. 10.1021/acs.jpclett.8b00968. 29763320

[ref14] Pascual J.; Kosta I.; Palacios-Lidon E.; Chuvilin A.; Grancini G.; Nazeeruddin M. K.; Grande H. J.; Delgado J. L.; Tena-Zaera R. Co-Solvent Effect in the Processing of the Perovskite: Fullerene Blend Films for Electron Transport Layer-Free Solar Cells. J. Phys. Chem. C 2018, 122 (5), 2512–2520. 10.1021/acs.jpcc.7b11141.

[ref15] Pascual J.; Collavini S.; Völker S. F.; Phung N.; Palacios-Lidon E.; Irusta L.; Grande H.-J.; Abate A.; Tena-Zaera R.; Delgado J. L. Unravelling Fullerene-Perovskite Interactions Introduces Advanced Blend Films for Performance-Improved Solar Cells. Sustainable Energy Fuels 2019, 3 (10), 2779–2787. 10.1039/C9SE00438F.

[ref16] Collavini S.; Saliba M.; Tress W. R.; Holzhey P. J.; Völker S. F.; Domanski K.; Turren-Cruz S. H.; Ummadisingu A.; Zakeeruddin S. M.; Hagfeldt A.; Grätzel M.; Delgado J. L. Poly(Ethylene Glycol)-[60]Fullerene-Based Materials for Perovskite Solar Cells with Improved Moisture Resistance and Reduced Hysteresis. ChemSusChem 2018, 11 (6), 1032–1039. 10.1002/cssc.201702265. 29285886

[ref17] Yuan J.; Hazarika A.; Zhao Q.; Ling X.; Moot T.; Ma W.; Luther J. M. Metal Halide Perovskites in Quantum Dot Solar Cells: Progress and Prospects. Joule 2020, 4 (6), 1160–1185. 10.1016/j.joule.2020.04.006.

[ref18] Cabrera-Espinoza A.; Collavini S.; Delgado J. L. Doping Strategies of Organic *n*-Type Materials in Perovskite Solar Cells: A Chemical Perspective. Sustainable Energy Fuels 2020, 4 (7), 3264–3281. 10.1039/D0SE00276C.

[ref19] Liu J.; Dong Q.; Wang M.; Ma H.; Pei M.; Bian J.; Shi Y. Efficient Planar Perovskite Solar Cells with Carbon Quantum Dot-Modified Spiro-MeOTAD as a Composite Hole Transport Layer. ACS Appl. Mater. Interfaces 2021, 13 (47), 56265–56272. 10.1021/acsami.1c18344. 34792324

[ref20] Wen X.; Wu J.; Gao D.; Lin C. Interfacial Engineering with Amino-Functionalized Graphene for Efficient Perovskite Solar Cells. J. Mater. Chem. A 2016, 4 (35), 13482–13487. 10.1039/C6TA04616A.

[ref21] Guo X.; Li J.; Wang B.; Zeng P.; Li F.; Yang Q.; Chen Y.; Liu M. Improving and Stabilizing Perovskite Solar Cells with Incorporation of Graphene in the Spiro-OMeTAD Layer: Suppressed Li Ions Migration and Improved Charge Extraction. ACS Appl. Energy Mater. 2020, 3 (1), 970–976. 10.1021/acsaem.9b02037.

[ref22] Li M.; Zuo W.; Wang Q.; Wang K.; Zhuo M.; Köbler H.; Halbig C. E.; Eigler S.; Yang Y.; Gao X.; Wang Z.; Li Y.; Abate A. Ultrathin Nanosheets of Oxo-Functionalized Graphene Inhibit the Ion Migration in Perovskite Solar Cells. Adv. Energy Mater. 2020, 10 (4), 1902653 10.1002/aenm.201902653.

[ref23] Choi J.; Han J.; Yoon J.; Kim S.; Jeon I.; Maruyama S. Overview and Outlook on Graphene and Carbon Nanotubes in Perovskite Photovoltaics from Single-Junction to Tandem Applications. Adv. Funct. Mater. 2022, 32 (42), 2204594 10.1002/adfm.202204594.

[ref24] Dong Z.; Li W.; Wang H.; Jiang X.; Liu H.; Zhu L.; Chen H. Carbon Nanotubes in Perovskite-Based Optoelectronic Devices. Matter 2022, 5 (2), 448–481. 10.1016/j.matt.2021.12.011.

[ref25] Iijima S.; Yudasaka M.; Yamada R.; Bandow S.; Suenaga K.; Kokai F.; Takahashi K. Nano-Aggregates of Single-Walled Graphitic Carbon Nanohorns. Chem. Phys. Lett. 1999, 309 (3–4), 165–170. 10.1016/S0009-2614(99)00642-9.

[ref26] Karousis N.; Suarez-Martinez I.; Ewels C. P.; Tagmatarchis N. Structure, Properties, Functionalization, and Applications of Carbon Nanohorns. Chem. Rev. 2016, 116 (8), 4850–4883. 10.1021/acs.chemrev.5b00611. 27074223

[ref27] Ojeda I.; Garcinuño B.; Moreno-Guzmán M.; González-Cortés A.; Yudasaka M.; Iijima S.; Langa F.; Yáñez-Sedeño P.; Pingarrón J. M. Carbon Nanohorns as a Scaffold for the Construction of Disposable Electrochemical Immunosensing Platforms. Application to the Determination of Fibrinogen in Human Plasma and Urine. Anal. Chem. 2014, 86 (15), 7749–7756. 10.1021/ac501681n. 25001594

[ref28] Vizuete M.; Gómez-Escalonilla M. J.; Fierro J. L. G.; Sandanayaka A. S. D.; Hasobe T.; Yudasaka M.; Iijima S.; Ito O.; Langa F. A Carbon Nanohorn-Porphyrin Supramolecular Assembly for Photoinduced Electron-Transfer Processes. Chem. - Eur. J. 2010, 16 (35), 10752–10763. 10.1002/chem.201000299. 20687144

[ref29] Völker S. F.; Collavini S.; Delgado J. L. Organic Charge Carriers for Perovskite Solar Cells. ChemSusChem 2015, 8 (18), 3012–3028. 10.1002/cssc.201500742. 26311591

[ref30] Habisreutinger S. N.; Leijtens T.; Eperon G. E.; Stranks S. D.; Nicholas R. J.; Snaith H. J. Enhanced Hole Extraction in Perovskite Solar Cells Through Carbon Nanotubes. J. Phys. Chem. Lett. 2014, 5 (23), 4207–4212. 10.1021/jz5021795. 26278955

[ref31] Lee J.; Menamparambath M. M.; Hwang J.-Y.; Baik S. Hierarchically Structured Hole Transport Layers of Spiro-OMeTAD and Multiwalled Carbon Nanotubes for Perovskite Solar Cells. ChemSusChem 2015, 8 (14), 2358–2362. 10.1002/cssc.201403462. 26013428

[ref32] Cai M.; Tiong V. T.; Hreid T.; Bell J.; Wang H. An Efficient Hole Transport Material Composite Based on Poly(3-Hexylthiophene) and Bamboo-Structured Carbon Nanotubes for High Performance Perovskite Solar Cells. J. Mater. Chem. A 2015, 3 (6), 2784–2793. 10.1039/C4TA04997G.

[ref33] Wang G.; Liu J.; Chen K.; Pathak R.; Gurung A.; Qiao Q. High-Performance Carbon Electrode-Based CsPbI_2_Br Inorganic Perovskite Solar Cell Based on Poly(3-Hexylthiophene)-Carbon Nanotubes Composite Hole-Transporting Layer. J. Colloid Interface Sci. 2019, 555, 180–186. 10.1016/j.jcis.2019.07.084. 31377644

[ref34] Mazzotta G.; Dollmann M.; Habisreutinger S. N.; Christoforo M. G.; Wang Z.; Snaith H. J.; Riede M. K.; Nicholas R. J. Solubilization of Carbon Nanotubes with Ethylene-Vinyl Acetate for Solution-Processed Conductive Films and Charge Extraction Layers in Perovskite Solar Cells. ACS Appl. Mater. Interfaces 2019, 11 (1), 1185–1191. 10.1021/acsami.8b15396. 30556995

[ref35] Wang J.; Li J.; Xu X.; Xu G.; Shen H. Enhanced Photovoltaic Performance with Carbon Nanotubes Incorporating into Hole Transport Materials for Perovskite Solar Cells. J. Electron. Mater. 2016, 45 (10), 5127–5132. 10.1007/s11664-016-4724-x.

[ref36] Miletić T.; Pavoni E.; Trifiletti V.; Rizzo A.; Listorti A.; Colella S.; Armaroli N.; Bonifazi D. Covalently Functionalized SWCNTs as Tailored P-Type Dopants for Perovskite Solar Cells. ACS Appl. Mater. Interfaces 2016, 8 (41), 27966–27973. 10.1021/acsami.6b08398. 27632080

[ref37] Gatti T.; Casaluci S.; Prato M.; Salerno M.; Di Stasio F.; Ansaldo A.; Menna E.; Di Carlo A.; Bonaccorso F. Boosting Perovskite Solar Cells Performance and Stability Through Doping a Poly-3(Hexylthiophene) Hole Transporting Material with Organic Functionalized Carbon Nanostructures. Adv. Funct. Mater. 2016, 26 (41), 7443–7453. 10.1002/adfm.201602803.

[ref38] Smith P. A. S. Azides and Nitrenes: Reactivity and Utility; Elsivier, 1984; pp 95–204.

[ref39] Wu H.-C.; Chang X.; Liu L.; Zhao F.; Zhao Y. Chemistry of Carbon Nanotubes in Biomedical Applications. J. Mater. Chem. 2010, 20 (6), 1036–1052. 10.1039/B911099M.

[ref40] Setaro A.; Adeli M.; Glaeske M.; Przyrembel D.; Bisswanger T.; Gordeev G.; Maschietto F.; Faghani A.; Paulus B.; Weinelt M.; Arenal R.; Haag R.; Reich S. Preserving π-Conjugation in Covalently Functionalized Carbon Nanotubes for Optoelectronic Applications. Nat. Commun. 2017, 8 (1), 14281 10.1038/ncomms14281. 28134240 PMC5290266

[ref41] Chen Z.; Nagase S.; Hirsch A.; Haddon R. C.; Thiel W.; von Ragué Schleyer P. Side-Wall Opening of Single-Walled Carbon Nanotubes (SWCNTs) by Chemical Modification: A Critical Theoretical Study. Angew. Chem., Int. Ed. 2004, 43 (12), 1552–1554. 10.1002/anie.200353087. 15022231

[ref42] Nakaie N.; Nakazawa T. Aniline Derivative, Charge-Transporting Varnish and Organic Electroluminescent Device, Europe Patent. EP3012245B12014.

[ref43] Zhang Q.; Ning Z.; Tian H. Click” Synthesis of Starburst Triphenylamine as Potential Emitting Material. Dyes Pigm. 2009, 81 (1), 80–84. 10.1016/j.dyepig.2008.09.005.

[ref44] Lv H.-j.; Ma R.; Zhang X.; Li M.; Wang Y.; Wang S.; Xing G. Surfactant-Modulated Discriminative Sensing of HNO and H_2_S with a Cu^2+^-Complex-Based Fluorescent Probe. Tetrahedron 2016, 72 (35), 5495–5501. 10.1016/j.tet.2016.07.039.

[ref45] Park S.; Srivastava D.; Cho K. Generalized Chemical Reactivity of Curved Surfaces: Carbon Nanotubes. Nano Lett. 2003, 3 (9), 1273–1277. 10.1021/nl0342747.

[ref46] Lefebvre J.; Maruyama S.; Finnie P. Photoluminescence: Science and Applications. In Carbon Nanotubes. Advanced Topics in the Synthesis, Structure, Properties and Applications; Springer-Verlag GmbH, 2008; Vol. 111, pp 286–318.

[ref47] Singh P.; Campidelli S.; Giordani S.; Bonifazi D.; Bianco A.; Prato M. Organic Functionalisation and Characterisation of Single-Walled Carbon Nanotubes. Chem. Soc. Rev. 2009, 38 (8), 2214–2230. 10.1039/b518111a. 19623345

[ref48] Cognet L.; Tsyboulski D. A.; Rocha J.-D. R.; Doyle C. D.; Tour J. M.; Weisman R. B. Stepwise Quenching of Exciton Fluorescence in Carbon Nanotubes by Single-Molecule Reactions. Science 2007, 316 (5830), 1465–1468. 10.1126/science.1141316. 17556581

[ref49] Stergiou A.; Liu Z.; Xu B.; Kaneko T.; Ewels C. P.; Suenaga K.; Zhang M.; Yudasaka M.; Tagmatarchis N. Individualized *p*-Doped Carbon Nanohorns. Angew. Chem., Int. Ed. 2016, 55 (35), 10468–10472. 10.1002/anie.201605644. 27444516

[ref50] Cioffi C.; Campidelli S.; Brunetti F. G.; Meneghetti M.; Prato M. Functionalisation of Carbon Nanohorns. Chem. Commun. 2006, (20), 2129–2131. 10.1039/b601176d. 16703130

[ref51] Rance G. A.; Marsh D. H.; Nicholas R. J.; Khlobystov A. N. UV–Vis Absorption Spectroscopy of Carbon Nanotubes: Relationship between the π-Electron Plasmon and Nanotube Diameter. Chem. Phys. Lett. 2010, 493 (1–3), 19–23. 10.1016/j.cplett.2010.05.012.

[ref52] Koyama T.; Asaka K.; Hikosaka N.; Kishida H.; Saito Y.; Nakamura A. Ultrafast Exciton Energy Transfer in Bundles of Single-Walled Carbon Nanotubes. J. Phys. Chem. Lett. 2011, 2 (3), 127–132. 10.1021/jz101635n.

[ref53] Erkens M.; Levshov D.; Wenseleers W.; Li H.; Flavel B. S.; Fagan J. A.; Popov V. N.; Avramenko M.; Forel S.; Flahaut E.; Cambré S. Efficient Inner-to-Outer Wall Energy Transfer in Highly Pure Double-Wall Carbon Nanotubes Revealed by Detailed Spectroscopy. ACS Nano 2022, 16 (10), 16038–16053. 10.1021/acsnano.2c03883. 36167339 PMC9620404

[ref54] Bandow S.; Rao A. M.; Sumanasekera G. U.; Eklund P. C.; Kokai F.; Takahashi K.; Yudasaka M.; Iijima S. Evidence for Anomalously Small Charge Transfer in Doped Single-Wall Carbon Nanohorn Aggregates with Li, K and Br. Appl. Phys. A: Mater. Sci. Process. 2000, 71 (5), 561–564. 10.1007/s003390000681.

[ref55] Kim K. K.; Bae J. J.; Park H. K.; Kim S. M.; Geng H.-Z.; Park K. A.; Shin H.-J.; Yoon S.-M.; Benayad A.; Choi J.-Y.; Lee Y. H. Fermi Level Engineering of Single-Walled Carbon Nanotubes by AuCl_3_ Doping. J. Am. Chem. Soc. 2008, 130 (38), 12757–12761. 10.1021/ja8038689. 18729358

[ref56] Das A.; Sood A. K.; Govindaraj A.; Saitta A. M.; Lazzeri M.; Mauri F.; Rao C. N. R. Doping in Carbon Nanotubes Probed by Raman and Transport Measurements. Phys. Rev. Lett. 2007, 99 (13), 136803 10.1103/PhysRevLett.99.136803. 17930620

[ref57] Seidel R.; Duesberg G. S.; Unger E.; Graham A. P.; Liebau M.; Kreupl F. Chemical Vapor Deposition Growth of Single-Walled Carbon Nanotubes at 600 °C and a Simple Growth Model. J. Phys. Chem. B 2004, 108 (6), 1888–1893. 10.1021/jp037063z.

[ref58] Dunens O. M.; MacKenzie K. J.; Harris A. T. Large-Scale Synthesis of Double-Walled Carbon Nanotubes in Fluidized Beds. Ind. Eng. Chem. Res. 2010, 49 (9), 4031–4035. 10.1021/ie100059q.

[ref59] Saliba M.; Correa-Baena J.-P.; Wolff C. M.; Stolterfoht M.; Phung N.; Albrecht S.; Neher D.; Abate A. How to Make Over 20% Efficient Perovskite Solar Cells in Regular (*n-i-p*) and Inverted (*p-i-n*) Architectures. Chem. Mater. 2018, 30 (13), 4193–4201. 10.1021/acs.chemmater.8b00136.

[ref60] Nakka L.; Cheng Y.; Aberle A. G.; Lin F. Analytical Review of Spiro-OMeTAD Hole Transport Materials: Paths Toward Stable and Efficient Perovskite Solar Cells. Adv. Energy Sustainability Res. 2022, 3 (8), 2200045 10.1002/aesr.202200045.

[ref61] Alvar M. S.; Blom P. W. M.; Wetzelaer G.-J. A. H. Space-Charge-Limited Electron and Hole Currents in Hybrid Organic-Inorganic Perovskites. Nat. Commun. 2020, 11 (1), 4023 10.1038/s41467-020-17868-0. 32782256 PMC7419305

[ref62] Ameri M.; Ghaffarkani M.; Ghahrizjani R. T.; Safari N.; Mohajerani E. Phenomenological Morphology Design of Hybrid Organic-Inorganic Perovskite Solar Cell for High Efficiency and Less Hysteresis. Sol. Energy Mater. Sol. Cells 2020, 205, 110251 10.1016/j.solmat.2019.110251.

[ref63] Rombach F. M.; Haque S. A.; Macdonald T. J. Lessons Learned from Spiro-OMeTAD and PTAA in Perovskite Solar Cells. Energy Environ. Sci. 2021, 14 (10), 5161–5190. 10.1039/D1EE02095A.

[ref64] Salado M.; Calió L.; Contreras-Bernal L.; Idígoras J.; Anta J.; Ahmad S.; Kazim S. Understanding the Influence of Interface Morphology on the Performance of Perovskite Solar Cells. Materials 2018, 11 (7), 1073 10.3390/ma11071073. 29941800 PMC6073852

[ref65] Cappel U. B.; Daeneke T.; Bach U. Oxygen-Induced Doping of Spiro-MeOTAD in Solid-State Dye-Sensitized Solar Cells and Its Impact on Device Performance. Nano Lett. 2012, 12 (9), 4925–4931. 10.1021/nl302509q. 22913390

[ref66] Freddi S.; Emelianov A. V.; Bobrinetskiy I. I.; Drera G.; Pagliara S.; Kopylova D. S.; Chiesa M.; Santini G.; Mores N.; Moscato U.; Nasibulin A. G.; Montuschi P.; Sangaletti L. Development of a Sensing Array for Human Breath Analysis Based on SWCNT Layers Functionalized with Semiconductor Organic Molecules. Adv. Healthcare Mater. 2020, 9 (12), 2000377 10.1002/adhm.202000377. 32378358

[ref67] Elawad M.; John K. I.; Idris A. M.; Yang L.; Gao Y. An Organic Hole-Transporting Material Spiro-OMeTAD Doped with a Mn Complex for Efficient Perovskite Solar Cells with High Conversion Efficiency. RSC Adv. 2021, 11 (52), 32730–32739. 10.1039/D1RA05906H. 35493571 PMC9042161

[ref68] Chiu S.-K.; Chung Y.-C.; Liou G.-S.; Su Y. O. Electrochemical and Spectral Characterizations of 9-Phenylcarbazoles. J. Chin. Chem. Soc. 2012, 59 (3), 331–337. 10.1002/jccs.201100601.

[ref69] Li X.; Chen Y.; Li L.; Huang J. Perovskite Thin Film Consisting with One-Dimensional Nanowires. Materials 2018, 11 (9), 1759 10.3390/ma11091759. 30231495 PMC6165021

[ref70] Peterson K. A.; Patterson A.; Vega-Flick A.; Liao B.; Chabinyc M. L. Doping Molecular Organic Semiconductors by Diffusion from the Vapor Phase. Mater. Chem. Front. 2020, 4 (12), 3632–3639. 10.1039/D0QM00442A.

[ref71] Shibayama N.; Maekawa H.; Nakamura Y.; Haruyama Y.; Niibe M.; Ito S. Control of Molecular Orientation of Spiro-OMeTAD on Substrates. ACS Appl. Mater. Interfaces 2020, 12 (44), 50187–50191. 10.1021/acsami.0c15509. 33084297

[ref72] Shi D.; Qin X.; Li Y.; He Y.; Zhong C.; Pan J.; Dong H.; Xu W.; Li T.; Hu W.; Brédas J.-L.; Bakr O. M. Spiro-OMeTAD Single Crystals: Remarkably Enhanced Charge-Carrier Transport (*via*) Mesoscale Ordering. Sci. Adv. 2016, 2 (4), e1501491 10.1126/sciadv.1501491. 27152342 PMC4846453

[ref73] Fukuhara T.; Tamai Y.; Ohkita H. Nongeminate Charge Recombination in Organic Photovoltaics. Sustainable Energy Fuels 2020, 4 (9), 4321–4351. 10.1039/D0SE00310G.

[ref74] Singh R.; Sandhu S.; Lee J.-J. Elucidating the Effect of Shunt Losses on the Performance of Mesoporous Perovskite Solar Cells. Sol. Energy 2019, 193, 956–961. 10.1016/j.solener.2019.10.018.

[ref75] Liao P.; Zhao X.; Li G.; Shen Y.; Wang M. A New Method for Fitting Current–Voltage Curves of Planar Heterojunction Perovskite Solar Cells. Nano-Micro Lett. 2018, 10 (1), 5 10.1007/s40820-017-0159-z. PMC6199049 30393654

[ref76] Proctor C. M.; Nguyen T.-Q. Effect of Leakage Current and Shunt Resistance on the Light Intensity Dependence of Organic Solar Cells. Appl. Phys. Lett. 2015, 106 (8), 083301 10.1063/1.4913589.

[ref77] Głowienka D.; Galagan Y. Light Intensity Analysis of Photovoltaic Parameters for Perovskite Solar Cells. Adv. Mater. 2022, 34 (2), 2105920 10.1002/adma.202105920. PMC11469270 34676926

[ref78] Gao P.; Tsao H. N.; Teuscher J.; Grätzel M. Organic Dyes Containing Fused Acenes as Building Blocks: Optical, Electrochemical and Photovoltaic Properties. Chin. Chem. Lett. 2018, 29 (2), 289–292. 10.1016/j.cclet.2017.09.056.

[ref79] Hasan M. S.; Alom J.; Asaduzzaman M.; Ahmed M. B.; Hossain M. D.; Saem A.; Masud J.; Thakare J.; Hossain M. A. Recent Criterion on Stability Enhancement of Perovskite Solar Cells. Processes 2022, 10 (7), 1408 10.3390/pr10071408.

