# Supplementary material for: p-Type Functionalized Carbon Nanohorns and Nanotubes in Perovskite Solar Cells
Source: ACS Appl Mater Interfaces. 2023 Sep 6;15(38):45212–28. doi: 10.1021/acsami.3c07476 (PMC10540139; doi:10.1021/acsami.3c07476)
Supplement: Supplementary file 1 — am3c07476_si_001.pdf [file am3c07476_si_001.pdf]

Supporting Information

p-Type Functionalized Carbon Nanohorns and Nanotubes in Perovskite Solar Cells

Helena Uceta,[‡a] Andrea Cabrera-Espinoza,[‡b] Myriam Barrejón,[a] José G. Sánchez,[c] Edgar Gutierrez-Fernandez,[b] Ivet Kosta,[d] Jaime Martín,[b] Silvia Collavini,[b] Eugenia Martínez-Ferrero,[c] Fernando Langa,[*a] Juan Luis Delgado[*b,e]

[a] *Instituto de Nanociencia, Nanotecnología y Materiales Moleculares (INAMOL), Universidad de Castilla-La Mancha, Avenida Carlos III S/N, Toledo 45071, Spain*

[b] *POLYMAT, University of the Basque Country UPV/EHU, Avenida Tolosa 72, Donostia/San Sebastián 20018, Spain*

[c] *Institute of Chemical Research of Catalonia-The Barcelona Institute of Science and Technology (ICIQ-BIST), Avinguda Països Catalans 16, Tarragona 43007, Spain*

[d] *CIDETEC, Basque Research and Technology Alliance (BRTA), Paseo Miramón 196, Donostia/San Sebastián 20014, Spain*

[e] *Ikerbasque, Basque Foundation for Science, Bilbao 48013, Spain*

[‡] These authors contributed equally to the manuscript

Corresponding Authors: Juan Luis Delgado (e-mail: juanluis.delgado@polymat.eu), Fernando Langa (e-mail: fernando.Langa@uclm.es)

Table of contents

1. Supplemental experimental section 2
  1.1. Synthetic procedure 2
    1.1.1. Synthesis of precursors 2
    1.1.2. Synthesis of functionalized carbon nanomaterials (CNMs) 3
  1.2. Synthetic characterization calculations 4
  1.3. Device characterization calculations 4
2. Supporting figures and tables 5
  2.1. Synthetic characterization 5
  2.2. Device characterization 19
References 24

1. Supplemental experimental section

1.1. Synthetic procedure

1.1.1. Synthesis of precursors

The synthesis of 4-amino-N,N-diphenylbenzenamine (TPA-NH_2), 4-azido-N,N-diphenylbenzenamine (TPA-N_3) and 9-(4-azidophenyl)-9H-carbazole (Cz-N_3) were performed following previously reported procedures with minor modifications.[1–3]

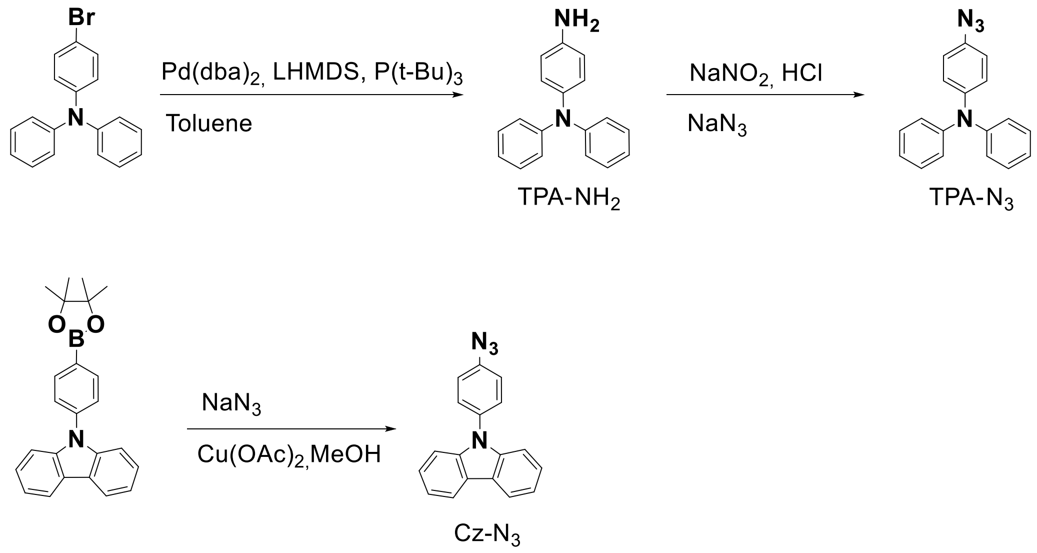

Scheme S1. Synthetic pathway for the preparation of TPA-NH_2, TPA-N_3 and Cz-N_3.

Synthesis of 4-amino-N,N-diphenylbenzenamine (TPA-NH_2):[1]

A solution of p-bromotriphenylamine (5 g, 15.4 mmol) and $Pd(dba)_2$ (0.445 g, 0.77 mmol, 5 mol %) in toluene (32 mL) was added dropwise to $LiN(Si(Me_3))_2$ (17 mL, 1 mol/L toluene solution). Afterwards, 6.5 mL of a toluene solution of $P(t\text{-}Bu)_3$ (24.02 g/L, 5 mol %) was added to the reaction mixture, followed by stirring at room temperature for 24 hours. After the stirring was completed, 3 mL hydrochloric acid was added to acidify the mixture and the reaction was stopped. Toluene was added thereto and the mixture was extracted with a liquid. The toluene layer was washed with 2 mol/L sodium hydroxide aqueous solution and water, and the solvent was distilled off from the toluene layer to obtain a crude product. The crude product was purified by column chromatography (Hexane/EtOAc, 9:1) to obtain the desired product as a yellow solid (3 g, 75 %).

^{1}H NMR (400 MHz, $CDCl_3$, δ): 7.21 (t, *J* = 7.7 Hz, 4H), 7.05 (d, *J* = 8.0 Hz, 4H), 6.95 (m, 4H), 6.69 (d, *J* = 8.2 Hz, 2H), 3.93 (bs, NH_2); ^{1}H NMR (400 MHz, DMSO-d_6, δ): 7.20 (t, *J* = 7.9 Hz, 4H), 6.89 (d, *J* = 8.4 Hz, 6H), 6.80 (d, *J* = 8.3 Hz, 2H), 6.56 (d. *J* = 8.6 Hz, 2H), 5.09 (s, NH_2); ^{13}C NMR (100 MHz, DMSO-d_6, δ):

147.96 (C-5), 146.33 (C-NH_2), 135.18 (C-4), 129.13 (C-7), 128.20 (C-3), 121.42 (C-6), 121.10 (C-8), 115.01 (C-2).

Synthesis of 4-azido-N,N-diphenylbenzenamine (TPA-N_3):[2]

A suspension of TPA-NH_2 (500 mg, 1.92 mmol) in hydrochloric acid (37 %, 0.5 mL) and Milli-Q water (5.5 mL) was cooled at 0-5 °C and diazotized with a solution of sodium nitrite (158.7 mg, 2.3 mmol) in Milli-Q water (2.15 mL). After being allowed to stand for a quarter, the resulting solution was treated dropwisely with sodium azide (187.2 mg, 2.88 mmol) in Milli-Q water (2.1 mL). The reaction was left for 15 minutes at 0 °C and then 90 minutes at room temperature. The mixture was extracted with Et_2O (3 x 20 mL). The organic layer was washed with saturated aqueous $NaHCO_3$ (3 x 20 mL), brine (20 mL), treated with anhydrous Na_2SO_4, filtered and the solvents were concentrated under reduced pressure. The crude product was purified by short column chromatography (Hexane) to obtain the desired product as yellow oil (106 mg, 21 %).

^{1}H NMR (400 MHz, $CDCl_3$, δ): 7.25 (m, 4H), 7.07 (t, J = 8.1 Hz, 6H), 7.02 (t, J = 7.3 Hz, 2H), 6.92 (d, J = 8.8 Hz, 2H); ^{1}H NMR (400 MHz, DMSO-d_6, δ): 7.28 (t, J = 7.7 Hz, 4H), 7.03 (m, 6H), 6.98 (d, J = 7.9 Hz, 4H); ^{13}C NMR (100 MHz, DMSO-d_6, δ): 147.12 (C-5), 144.50 (C-4), 133.64 (C-N3), 129.60 (C-7), 125.36 (C-3), 123.59 (C-6), 123.02 (C-8), 120.33 (C-2); ATR-FTIR: v = 2091 cm^{-1} (-N=N=N).

Synthesis of 9-(4-Azidophenyl)-9H-carbazole (Cz-N_3, 3):[3]

To a solution of 9-(4-(4,4,5,5-Tetramethyl-1,3,2-dioxaborolan-2-yl) phenyl)-9H-carbazole (250 mg, 0.677 mmol) in methanol (14 mL), NaN_3 (66 mg, 1.01 mmol) and $Cu(OAc)_2$ (13.5 mg, 0.0677 mmol) were added. The mixture was stirred in a 55 °C of oil bath under Ar for about 2 h in dark conditions. The crude yellow oil was then diluted with EtOAc, washed with saturated NaCl solution, and dried with Na_2SO_4. After removal of the solvent, the mixture was purified by short column chromatography (Hexane) to give the desired product as yellow crystals (180 mg, 94 %).

^{1}H NMR (400 MHz, $CDCl_3$, δ): 8.16 (d, J = 7.7 Hz, 2H), 7.57 (d, J = 7.57 Hz, 2H), 7.38-7.26 (m, 8H); ^{1}H NMR (400 MHz, DMSO-d_6, δ): 8.25 (d, J = 7.7 Hz, 2H), 7.67 (d, J = 8.6 Hz, 2H), 7.42 (t, J = 7.4 Hz, 4H), 7.35 (d, J = 8.1 Hz, 2H), 7.29 (t, J = 7.3 Hz, 2H); ^{13}C NMR (100 MHz, DMSO-d_6, δ): 140.19 (C-5), 138.68 (C-N3), 133.65 (C-4), 128.48 (C-3), 126.37 (C-7), 122.73 (C-10), 120.89 (C-9), 120.63 (C-2), 120.18 (C-8), 109.57 (C-6); ATR-FTIR: v = 2084 cm^{-1} (-N=N=N).

1.1.2. Synthesis of functionalized carbon nanomaterials (CNMs):

The synthesis of functionalized carbon nanomaterials were carried out following the general procedure described in the "Experimental section" of the main text.

Single-walled carbon nanohorns-triphenylamine (CNHs-TPA): to a dispersion of pristine CNHs (50 mg) in NMP (160 mL) was added TPA-N3 (250 mg, 5 eq. on weight). The reaction mixture was stirred at r.t. for 24 h affording the desired functionalized CNHs as a black solid (49.5 mg).

Single-walled carbon nanotubes-triphenylamine (SWCNTs-TPA): to a dispersion of pristine SWCNTs (40 mg) in NMP (125 mL) was added TPA-N3 (200 mg, 5 eq. on weight). The reaction mixture was stirred at r.t. for 24 h affording the desired functionalized SWCNTs as a black solid (45.5 mg).

Double-walled carbon nanotubes-triphenylamine (DWCNTs-TPA): to a dispersion of pristine DWCNTs (50 mg) in NMP (166 mL) was added TPA-N3 (2) (250 mg, 5 eq. on weight). The reaction mixture was stirred at r.t. for 48 h affording the desired functionalized DWCNTs as a black solid (51.2 mg).

Single-walled carbon nanohorns-carbazole (CNHs-Cz): to a dispersion of pristine CNHs (50 mg) in NMP (166 mL) was added Cz-N3 (250 mg, 5 eq. on weight). The reaction mixture was stirred at r.t. for 24 h affording the desired functionalized CNHs as a black solid (55.5 mg).

Single-walled carbon nanotubes-carbazole (SWCNTs-Cz): to a dispersion of pristine SWCNTs (50 mg) in NMP (166 mL) was added Cz-N3 (250 mg, 5 eq. on weight). The reaction mixture was stirred at r.t. for 24 h affording the desired functionalized SWCNTs as a black solid (53.8 mg).

Double walled carbon nanotubes-carbazole (DWCNTs-Cz): to a dispersion of pristine DWCNTs (50 mg) in NMP (166 mL) was added Cz-N3 (250 mg, 5 eq. on weight). The reaction mixture was stirred at r.t. for 48 h affording the desired functionalized DWCNTs as a black solid (56.6 mg)."

1.2. Synthetic characterization calculations

Thermogravimetric analysis-derivative thermogravimetry (TGA-DTG): The weight changes were recorded as a function of temperature. The functional group coverages (FGC) were calculated considering the weight loss percentages of the organic addends (wt $\%_{\text{Addend}}$) and pristine CNMs (wt $\%_{\text{CNM}}$), the molecular weights of the expected anchored addends ($\text{MW}_{\text{addend}}$) and atomic mass of carbon, according to the following Equation S1:[4,5]

$$\text{FCG} = \frac{\text{wt}\%_{\text{CNM}}/12.01}{\text{wt}\%_{\text{Addend}}/\text{Mw}_{\text{Addend}}} \qquad \text{Equation S1}$$

The functionalization degree (FD in µmol g^{-1}) for each functionalized CNM was calculated according to Equation S2, considering the weight loss percentage of the organic addends after having subtracted the analogous weight loss from the pristine CNMs (L) and the molecular weights of the expected anchored addends ($\text{MW}_{\text{addend}}$). The conversion factor 10^4 provides the data in the desired unities (µmol g^{-1}).[6]

$$\text{FD}\ (\mu\text{mol/g}) = \frac{L\,\%\cdot 10^4}{\text{Mw}_{\text{Addend}}\ (\text{g}/mol)} \qquad \text{Equation S2}$$

1.3. Device characterization calculations

Space-charge limited current (SCLC) method: The hole mobility values were calculated by analyzing the dark current-voltage (*J*-*V*) curves using the Mott-Gurney model, with measurements taken in a forward

scan from 0 V to 1.2 V in dark conditions, at room temperature, and under a nitrogen atmosphere. The equation used was $J = 9\varepsilon_0\varepsilon_r\mu_h V^2/8L^3$, where J is the current density, L is the thickness of the HTL, μ_h is the hole mobility, ε_r is the relative dielectric constant of the transport medium, ε_0 is the permittivity of vacuum and V is the applied voltage. The fitting was applied in the region where $J \propto V^2$.

Photoluminescence measurements: The time-resolved photoluminescence decays were fitted using the following bi-exponential equation:

$$\tau(t) = A_1 e^{-\left(\frac{t}{\tau 1}\right)} + A_2 e^{-\left(\frac{t}{\tau 2}\right)} \qquad \text{Equation S3}$$

Where A_1 and A_2 are the amplitude of the radiative decay lifetime and τ represents the lifetime values. The average lifetime (τ_{avg}) was calculated as follows the equation: $\tau_{avg} = \sum \alpha_i \tau_i \, , where \; \alpha_i = A_i \tau_i / \sum A_i \tau_i$.

2. Supporting figures and tables

2.1. Synthetic characterization

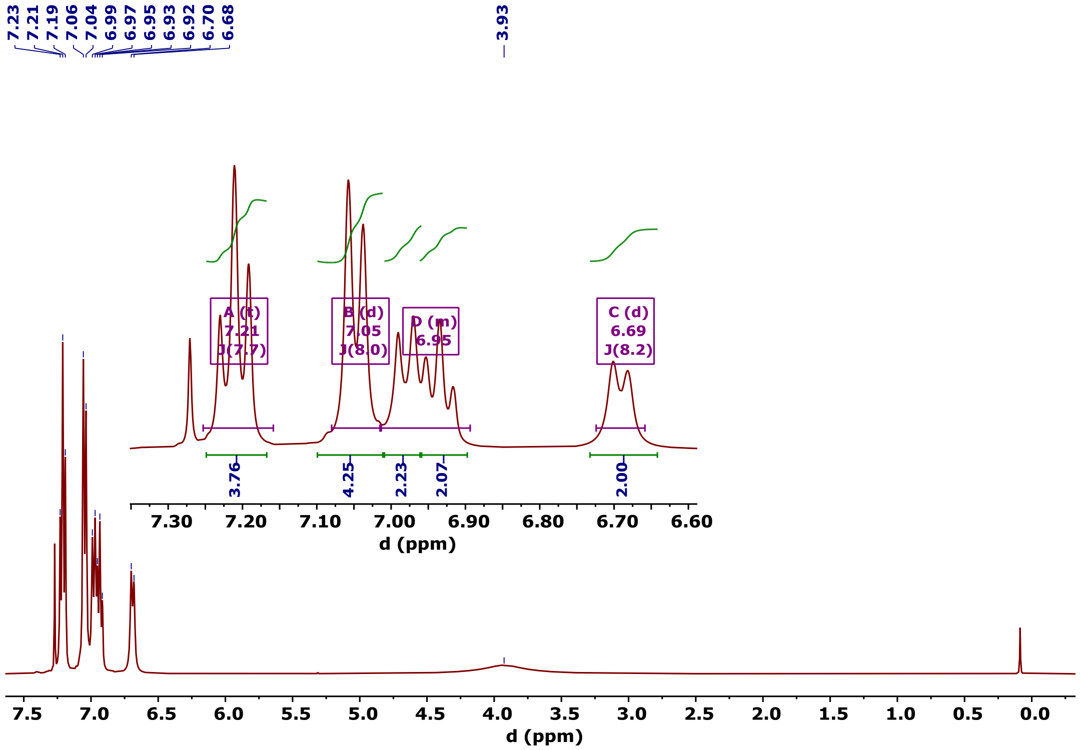

Figure S1. ^{1}H NMR spectrum (400 MHz, $CDCl_3$) of TPA-NH_2.

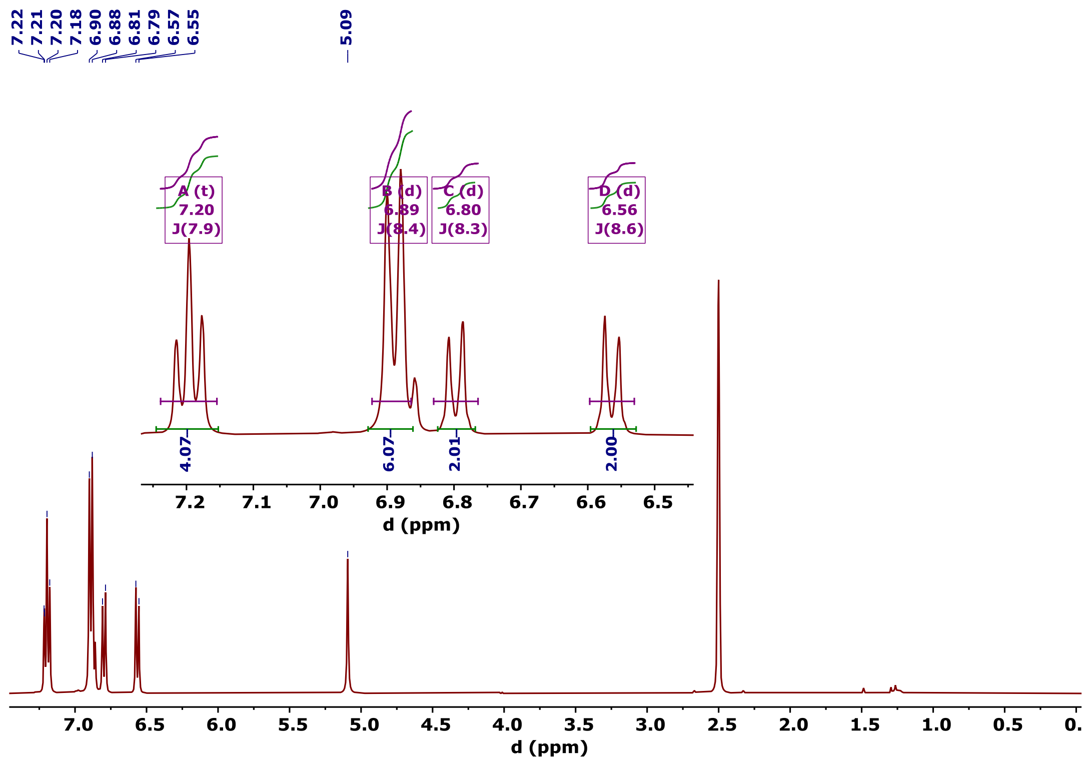

Figure S2. ^{1}H NMR spectrum (400 MHz, DMSO-*d_6*) of TPA-NH_2.

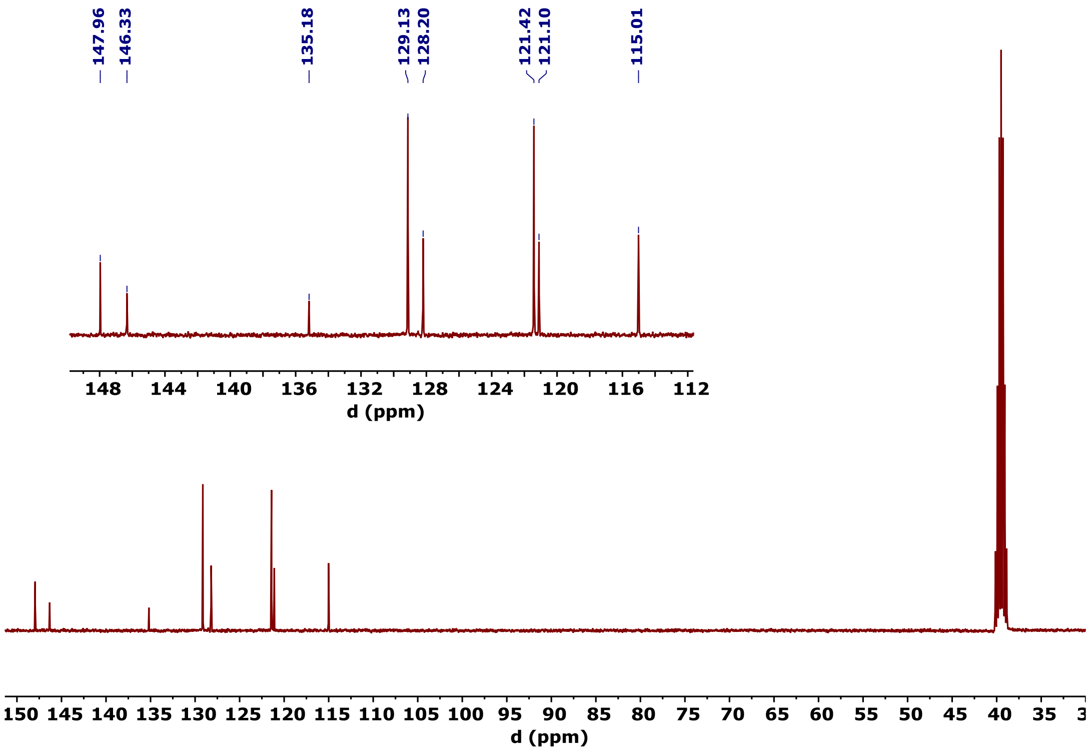

Figure S3. 13C NMR spectrum (100 MHz, DMSO-d_6) of TPA-NH_2.

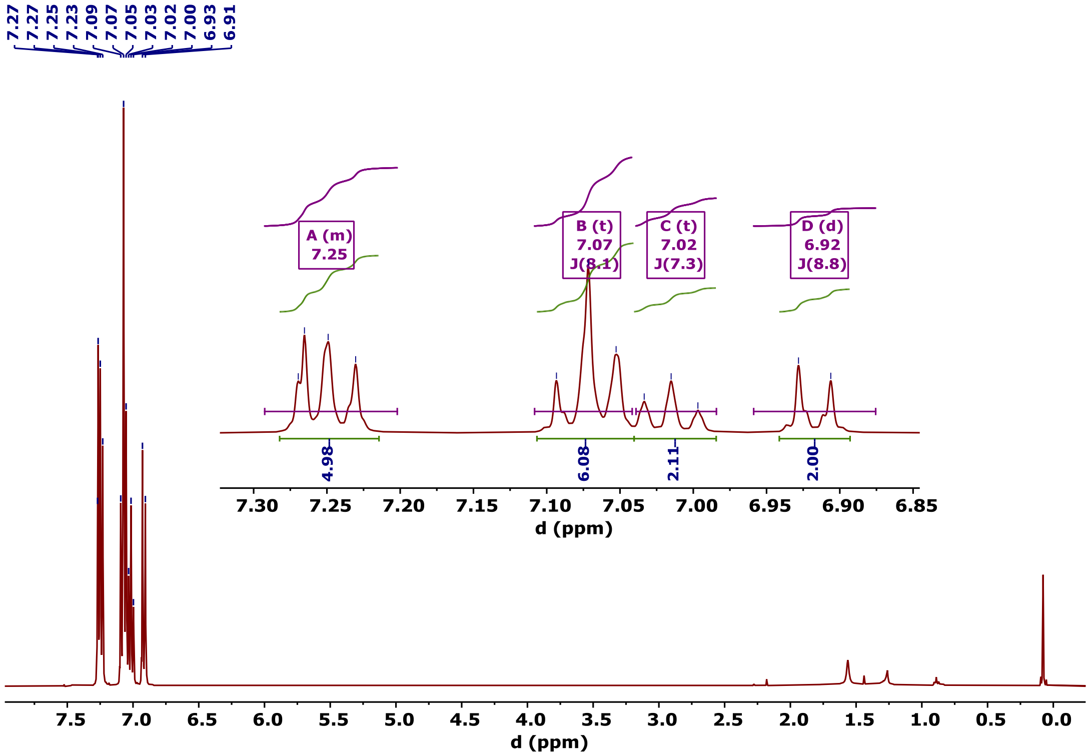

Figure S4. ^1H NMR spectrum (400 MHz, $CDCl_3$) of TPA-N_3.

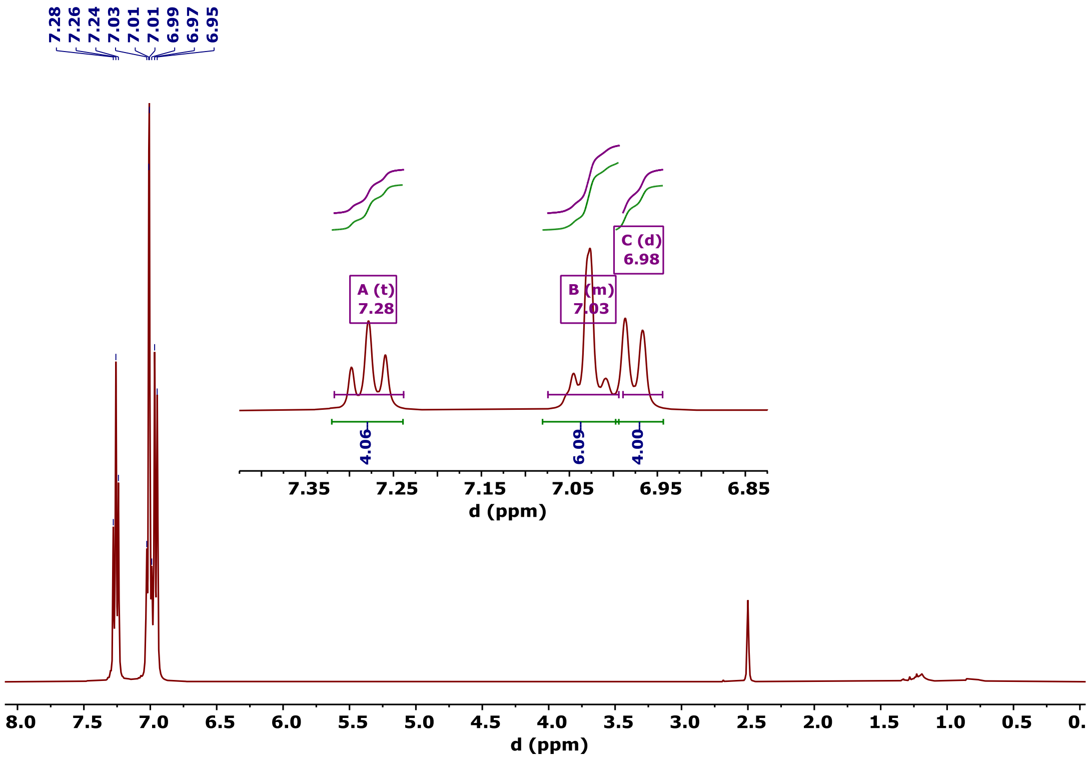

Figure S5. ^1H NMR spectrum (400 MHz, DMSO-d_6) of TPA-N_3

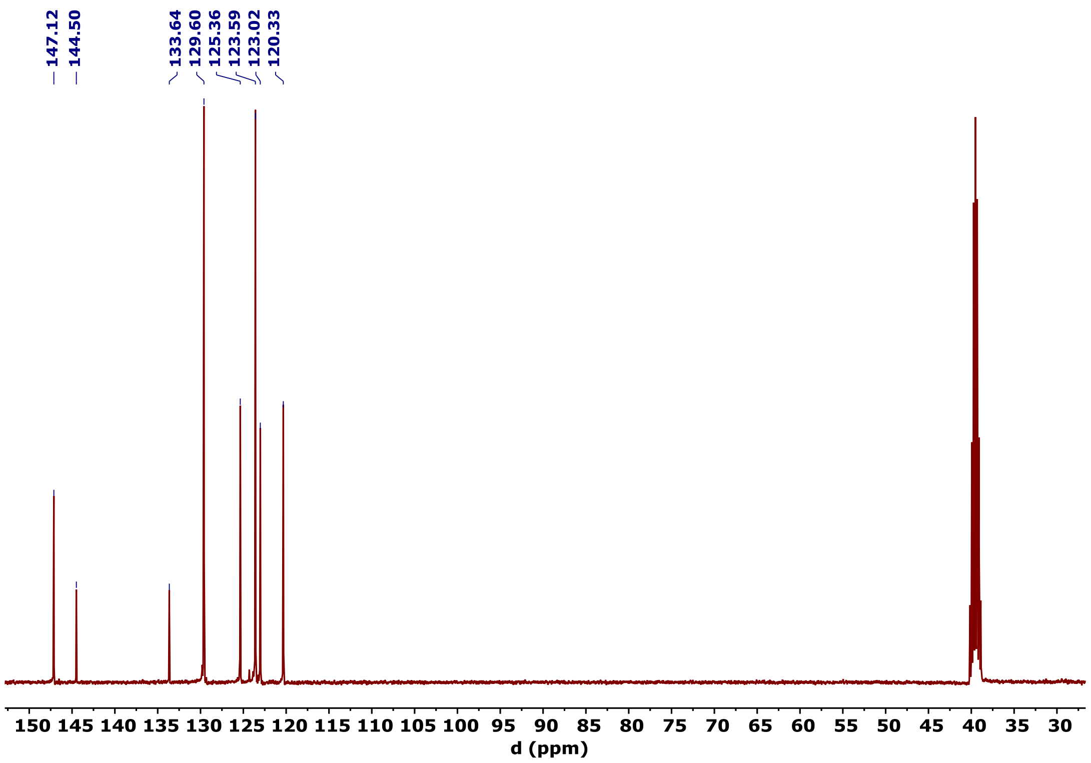

Figure S6. ^{13}C NMR spectrum (100 MHz, DMSO-d_6) of TPA-N_3.

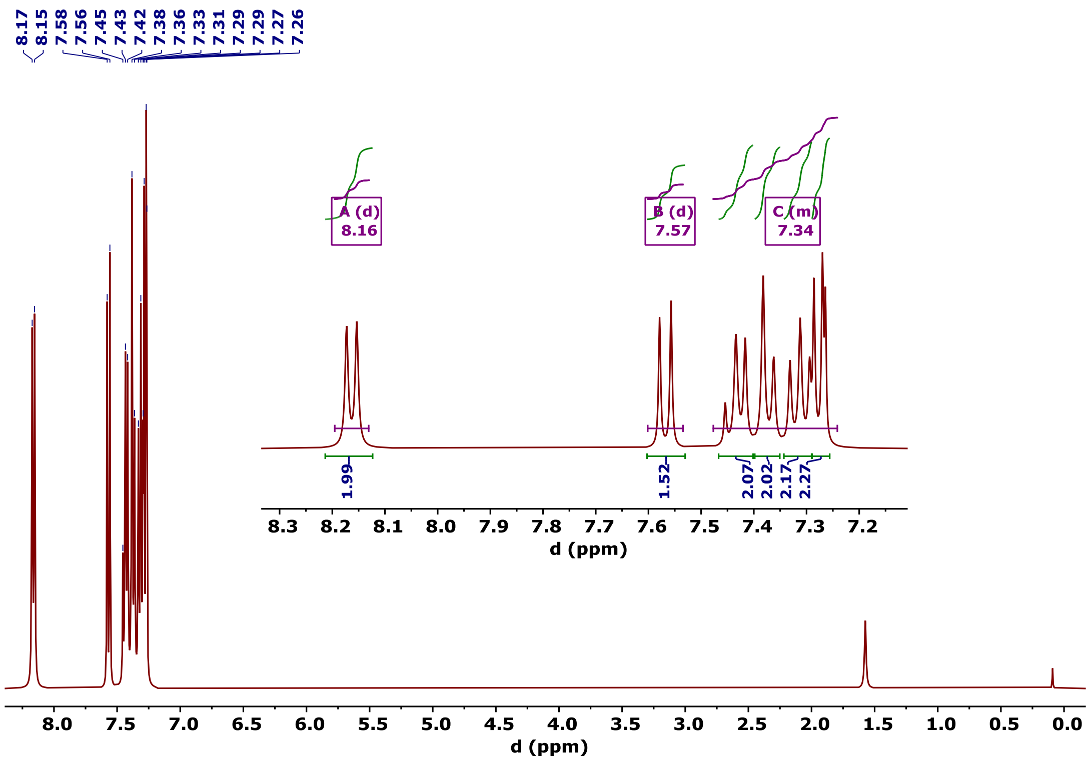

Figure S7. ^{1}H NMR spectrum (400 MHz, $CDCl_3$) of Cz-N_3.

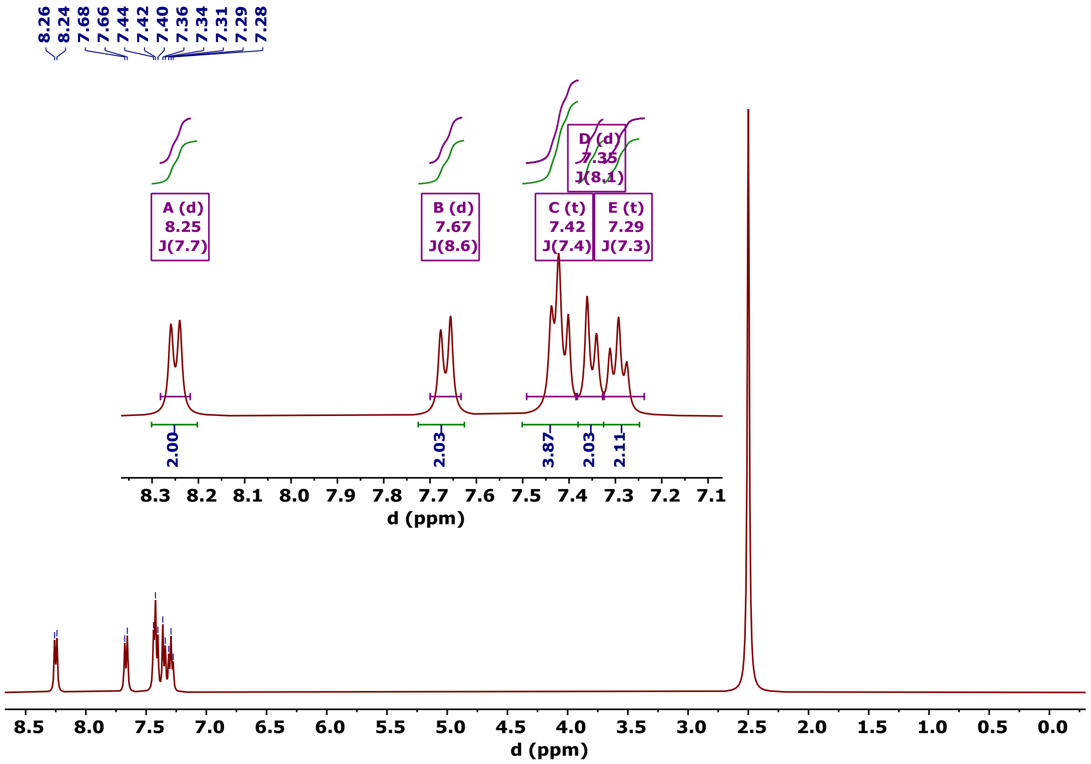

Figure S8. ^{1}H NMR spectrum (400 MHz, DMSO-d_6) of Cz-N_3.

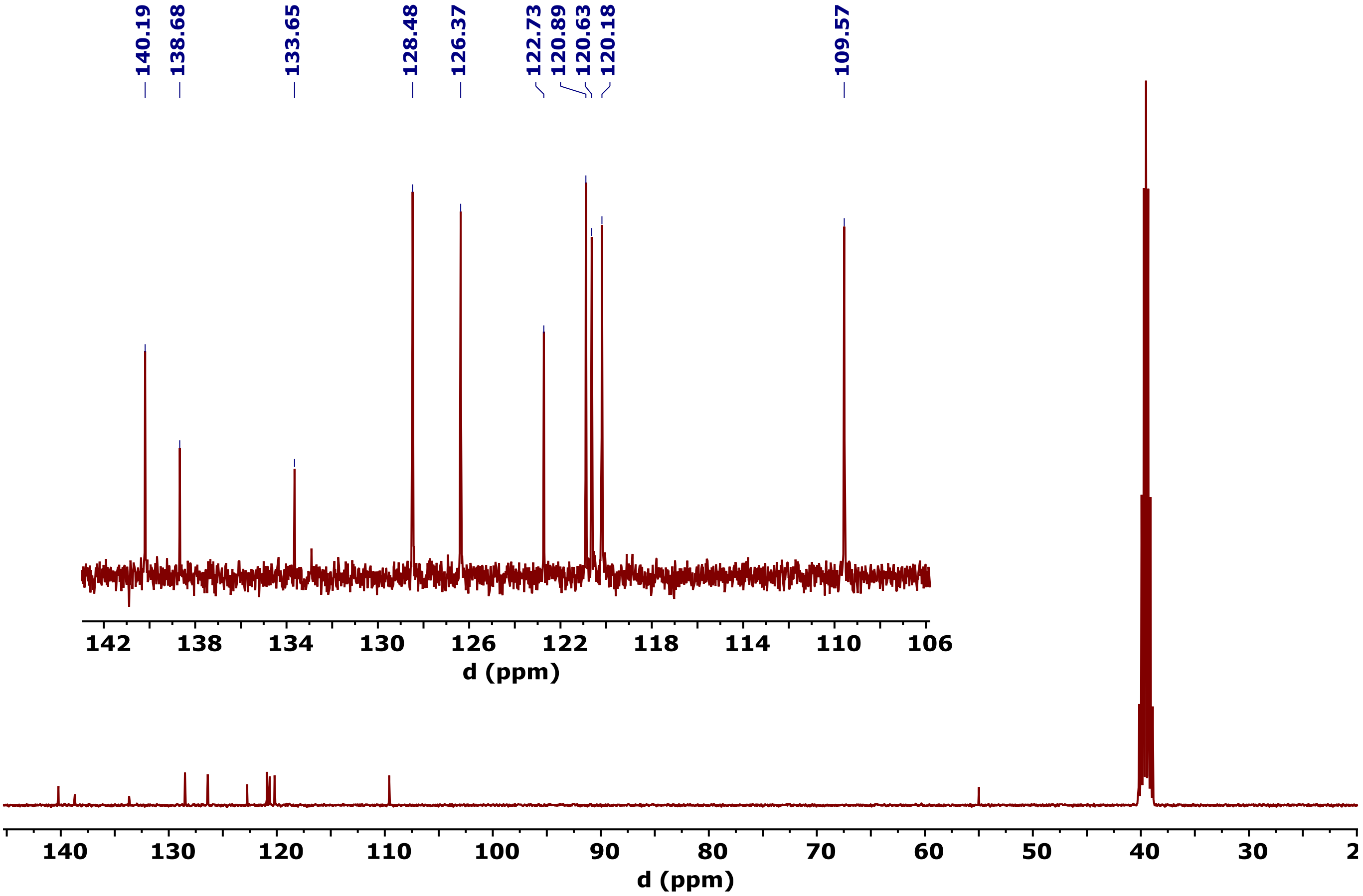

Figure S9. ^{13}C NMR spectrum (100 MHz, DMSO-d_6) of Cz-N_3.

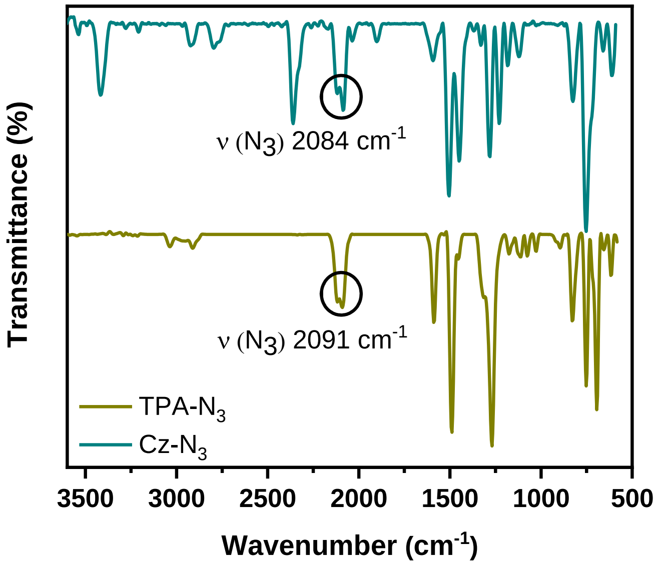

Figure S10. ATR-FTIR spectra of TPA-N_3 and Cz-N_3 recorded at room temperature.

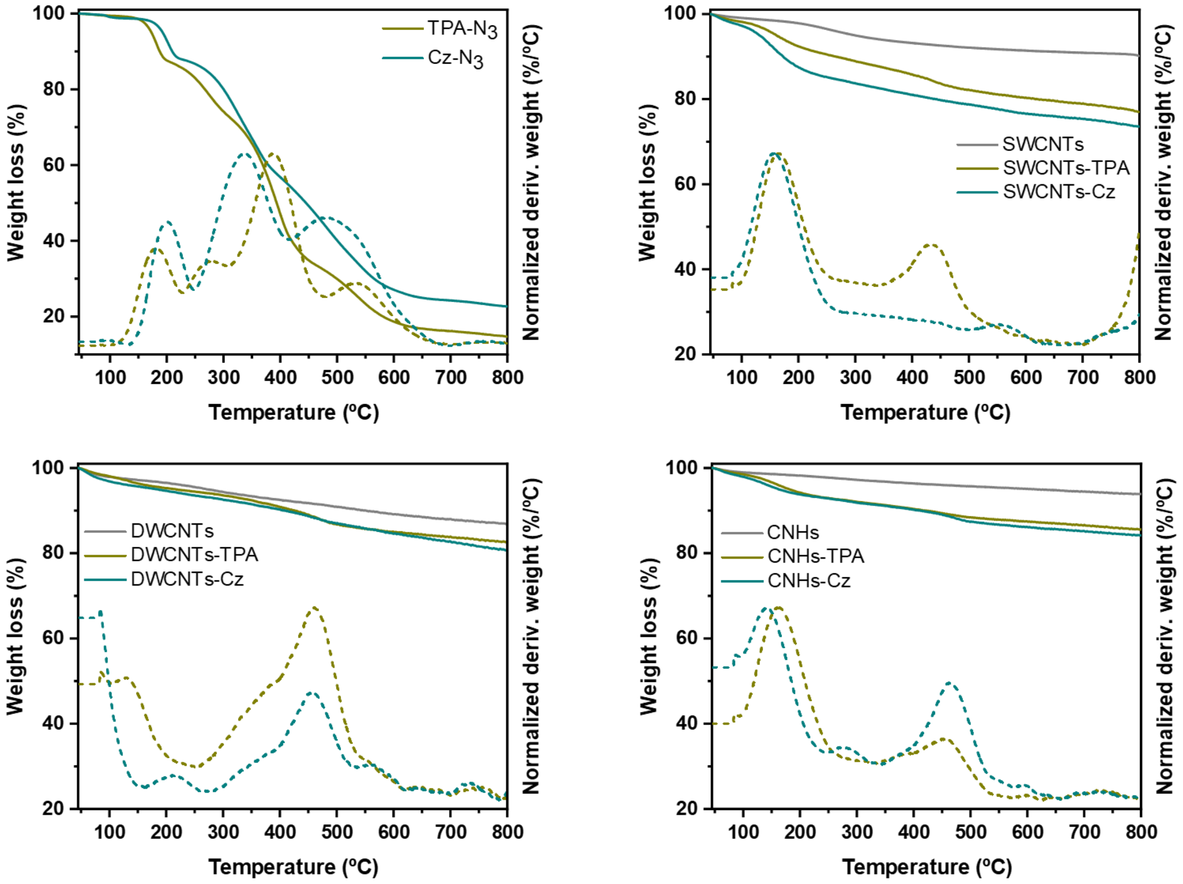

Figure S11. TGA plots and DTG curves of TPA-N_3, CZ-N_3, pristine and functionalized CNMs, recorded at 10 ºC/min under N_2.

Table S1. TGA and Raman data for pristine and functionalized CNMs.

Sample	TGA weight loss (%)[a]	FGC[b]	FD (μmol/g CNM)[c]	$\Delta I_D/I_G$[d]
SWCNTs	8.8			0.10
SWCNTs-TPA	11.6	96	448	0.12
SWNTs-Cz	15.3	81	593	0.11
DWCNTs	11.5			0.02
DWCNTs-TPA	4.1	122	158	0.03
DWCNTs-Cz	4.8	116	187	0.03
CNHs	5.2			1.86
CNHs-TPA	7.8	156	330	1.85
CNHs-Cz	9.2	141	357	1.87

a) TGA weight loss % at 650 ºC, b) estimated functional group coverages, c) functionalization degree (μmol of functional groups per gram of CNM) determined at 650 ºC, d) average I_D/I_G of 400 independent measurements (laser excitation λ= 785 nm).

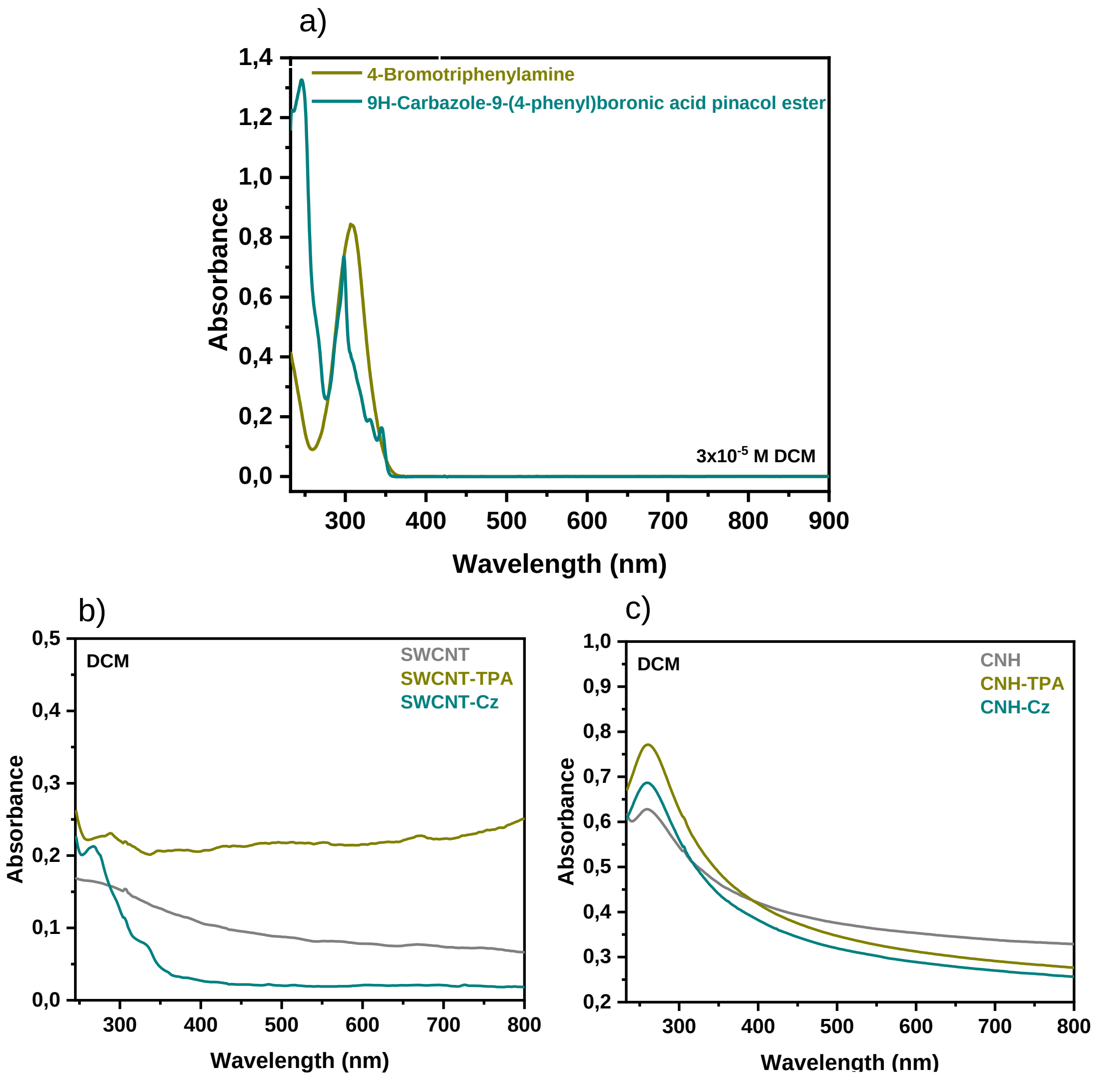

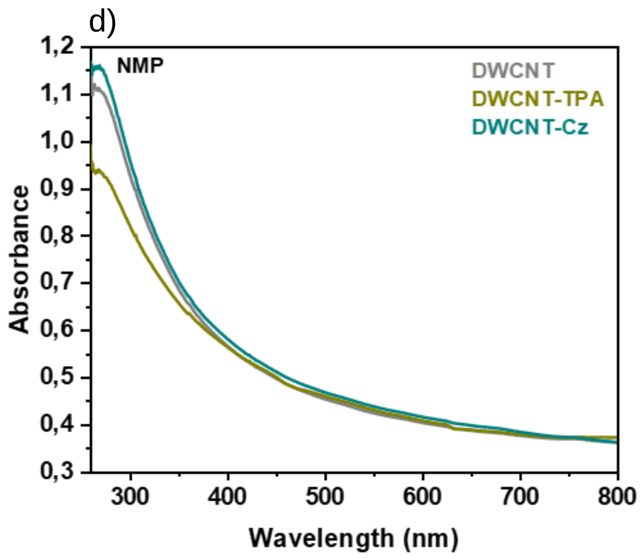

Figure S12.UV-Vis absorption spectra of the functionalized CNMs compared to their corresponding pristine materials in different solvents.

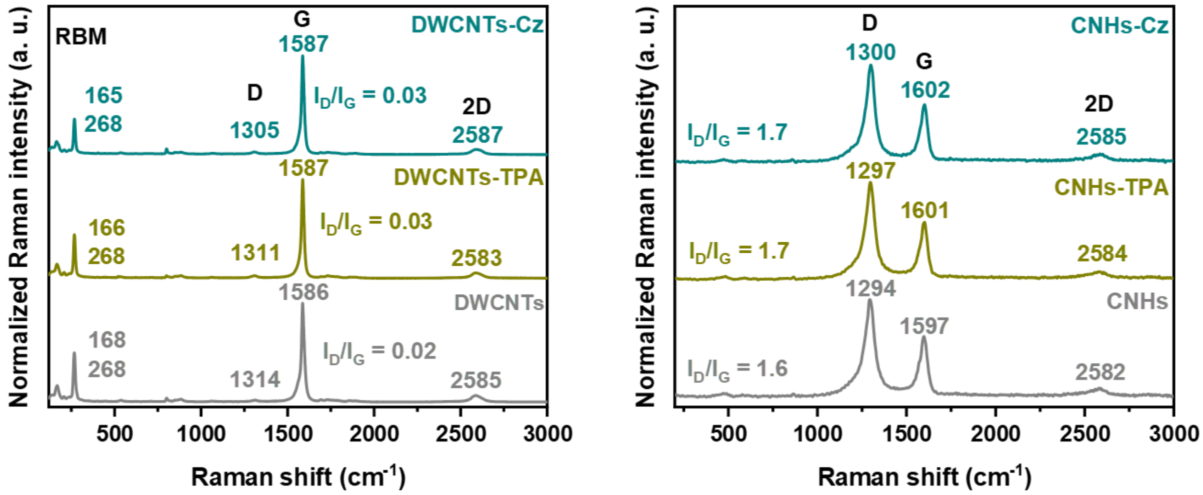

Figure S13. Raman extended spectra of pristine DWCNTs and CNHs compared to the functionalized hybrids materials. Laser excitation λ= 785 nm.

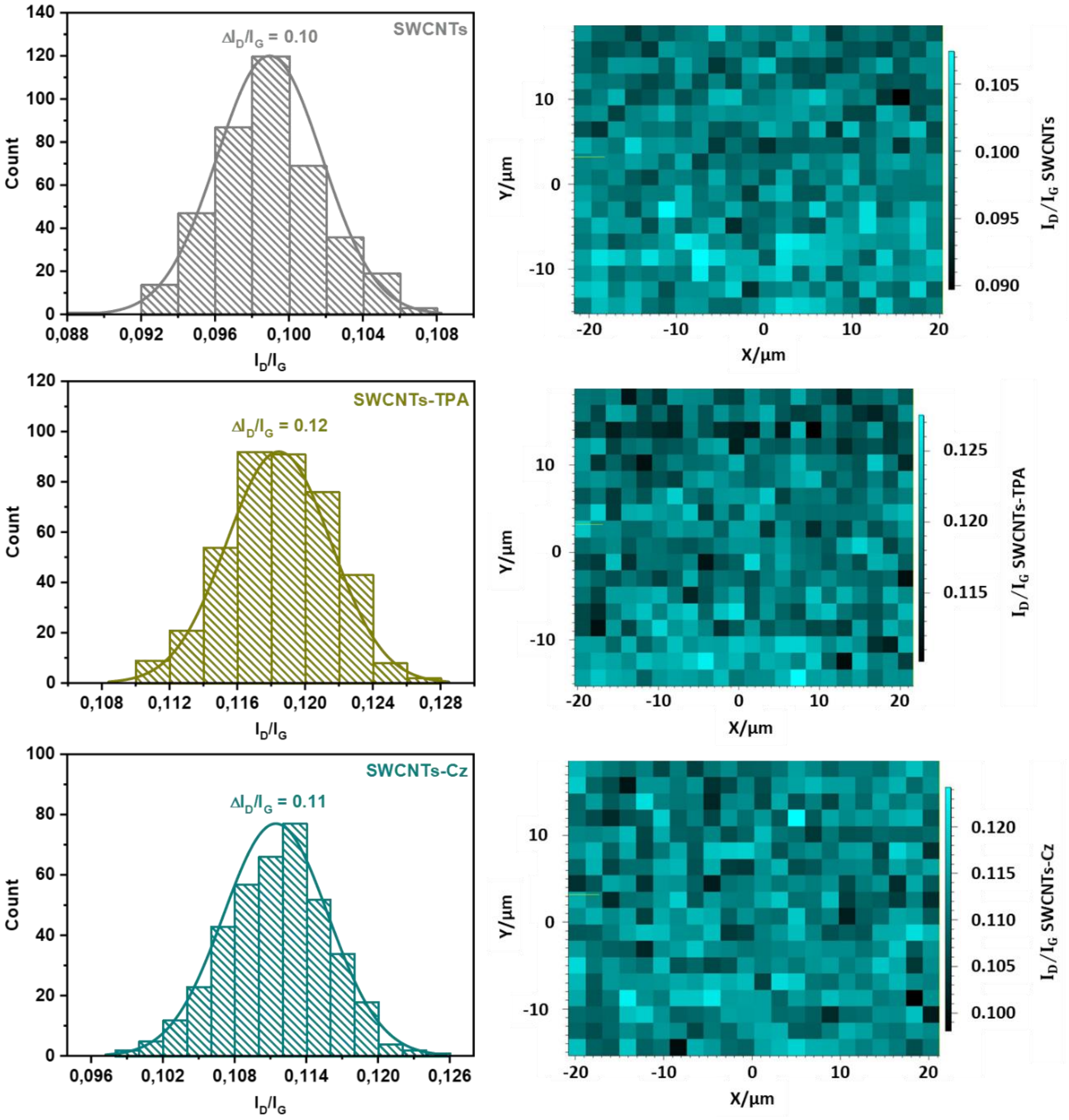

Figure S14. I_D/I_G histograms of pristine SWCNTs and the corresponding functionalized SWCNTs (TPA-SWCNTs and Cz-SWCNTs) (left), and Raman maps on the right side of the figure. Data werecalculated from the average of 400 individual measurements taken over the sample with an excitation wavelength of 785 nm.

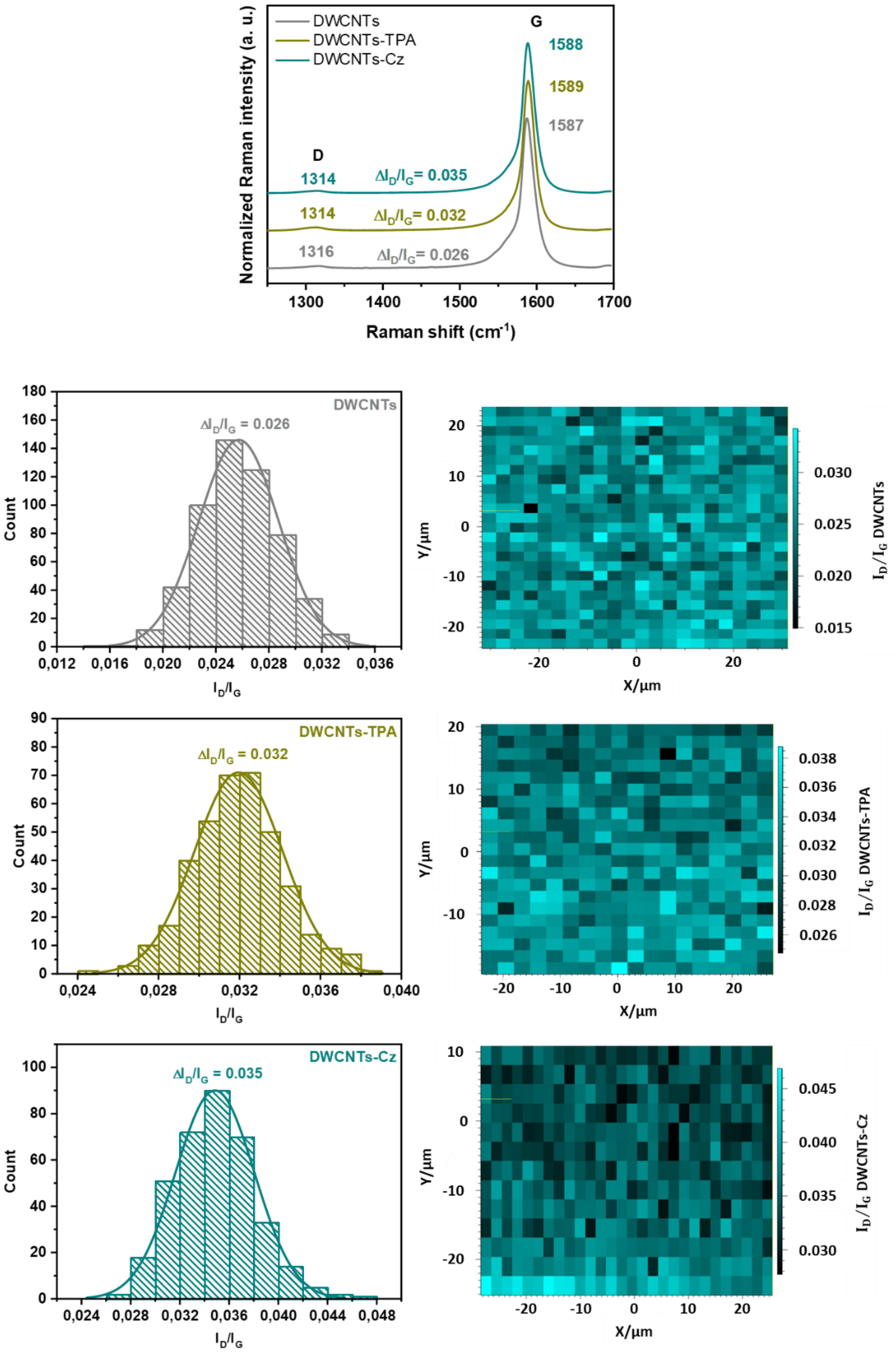

Figure S15. Raman average spectra of DWCNTs and the corresponding functionalized samples (top). I_D/I_G histograms of pristine DWCNTs and the corresponding functionalized DWCNTs (DWCNTs-TPA and DWCNTs-Cz) (left), and Raman maps on the right side of the figure. Data were calculated from the average of 400 individual measurements taken over the sample with an excitation wavelength of 785 nm.

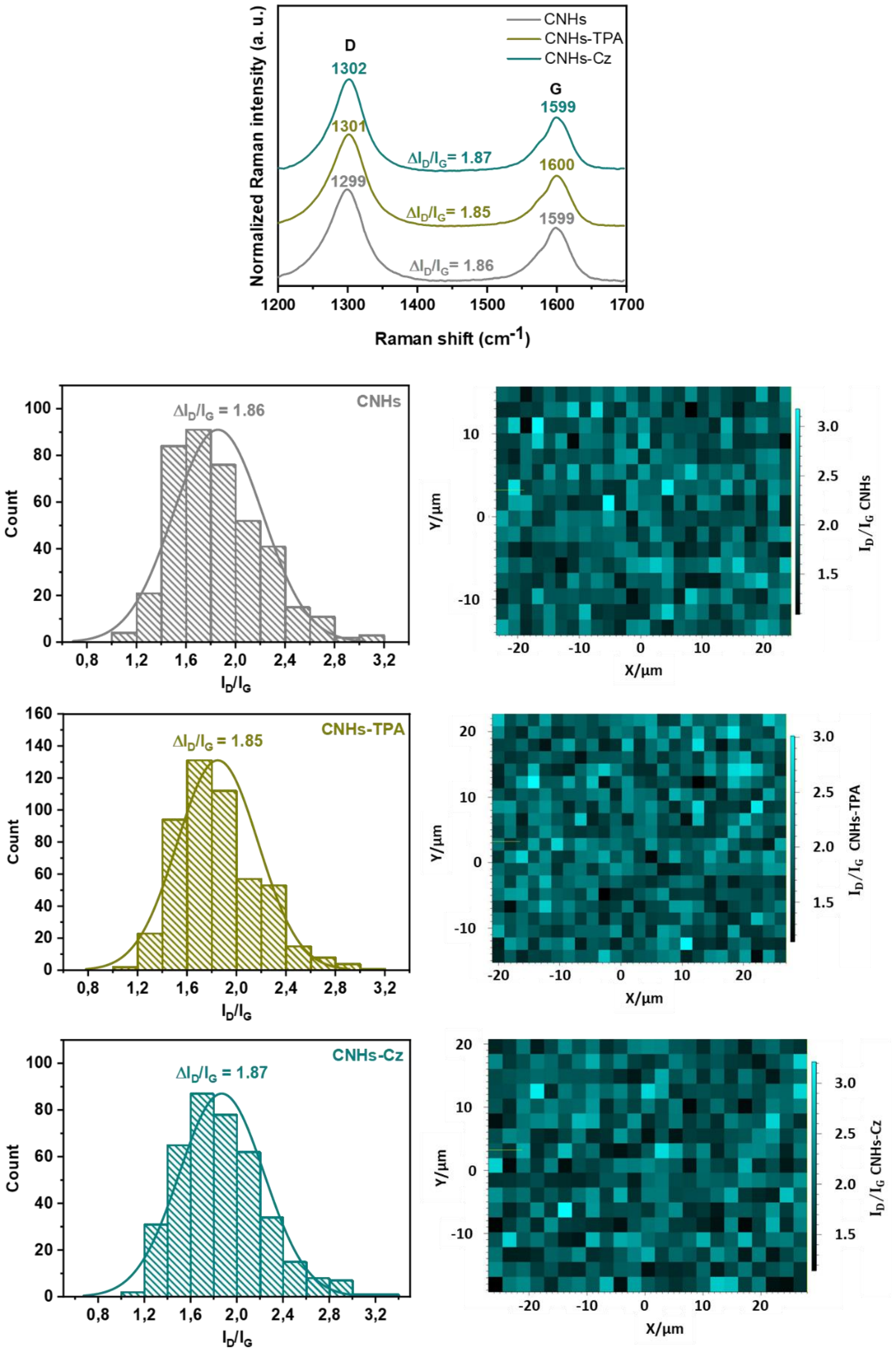

Figure S16. Raman average spectra of CNHs and the corresponding functionalized samples (top). I_D/I_G histograms of pristine CNHs and the corresponding functionalized CNHs (CNHs-TPA and CNHs-Cz) (left), and Raman maps on the right side of the figure. Data were calculated from the average of 400 individual measurements taken over the sample with an excitation wavelength of 785 nm.

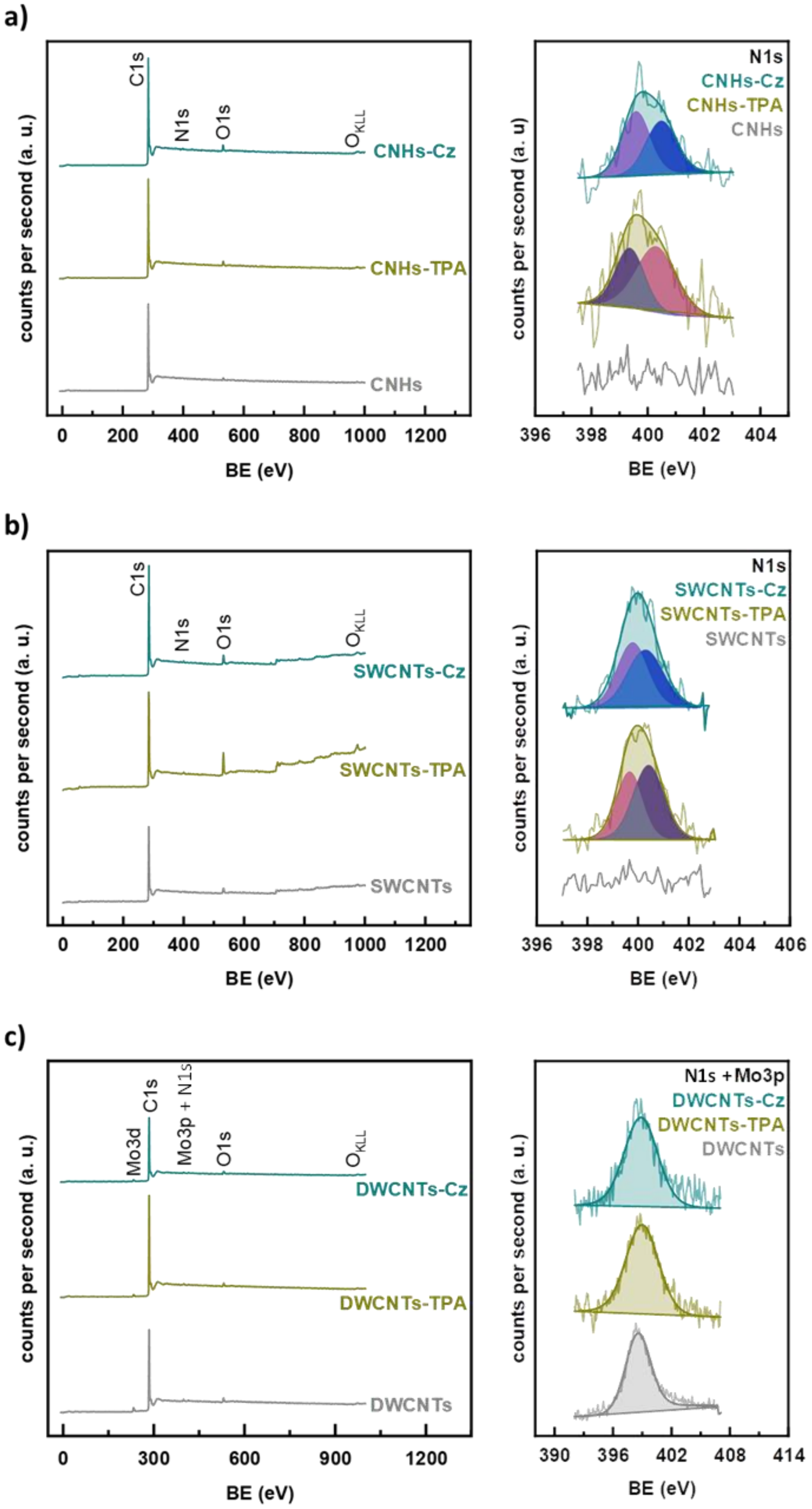

Figure S17. Survey spectra and high-resolution N1s spectra for pristine and functionalized CNMs.

Table S2. Atomic percentages for pristine and functionalized CNMs determined from XPS analysis.

Sample	C1s (at%)	O1s (at%)	N1s (at%)
CNHs	99.14	0.86	-
CNHs-TPA	97.73	1.81	0.45
CNHs-Cz	97.64	1.96	0.39
SWCNTs	96.55	3.45	-
SWCNTs-TPA	90.26	8.83	0.91
SWCNTs-Cz	94.28	4.83	0.89
DWCNTs	94.74	2.53	-
DWCNTs-TPA	97.13	1.55	1.02*
DWCNTs-Cz	96.42	2.09	1.02*

* XPS atomic percentage of N1s + Mo3p

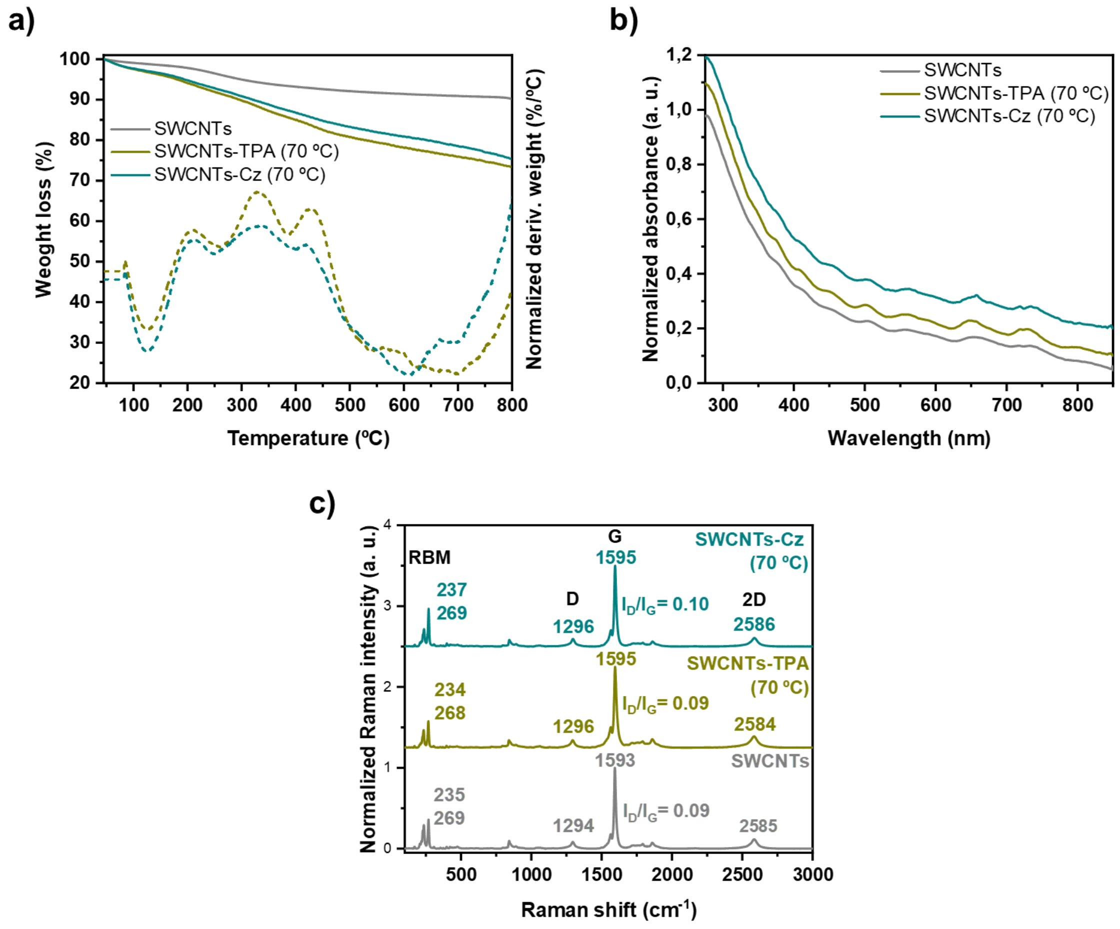

Figure S18. a) TGA plots and DTG curves recorded at 10 ºC/min under N_2, b) UV-Vis absorption spectra and c) Raman extended spectra (laser excitation λ= 785 nm) of pristine SWCNTs compared to functionalized SWCNTs-TPA and SWCNTs-Cz at 70 ºC.

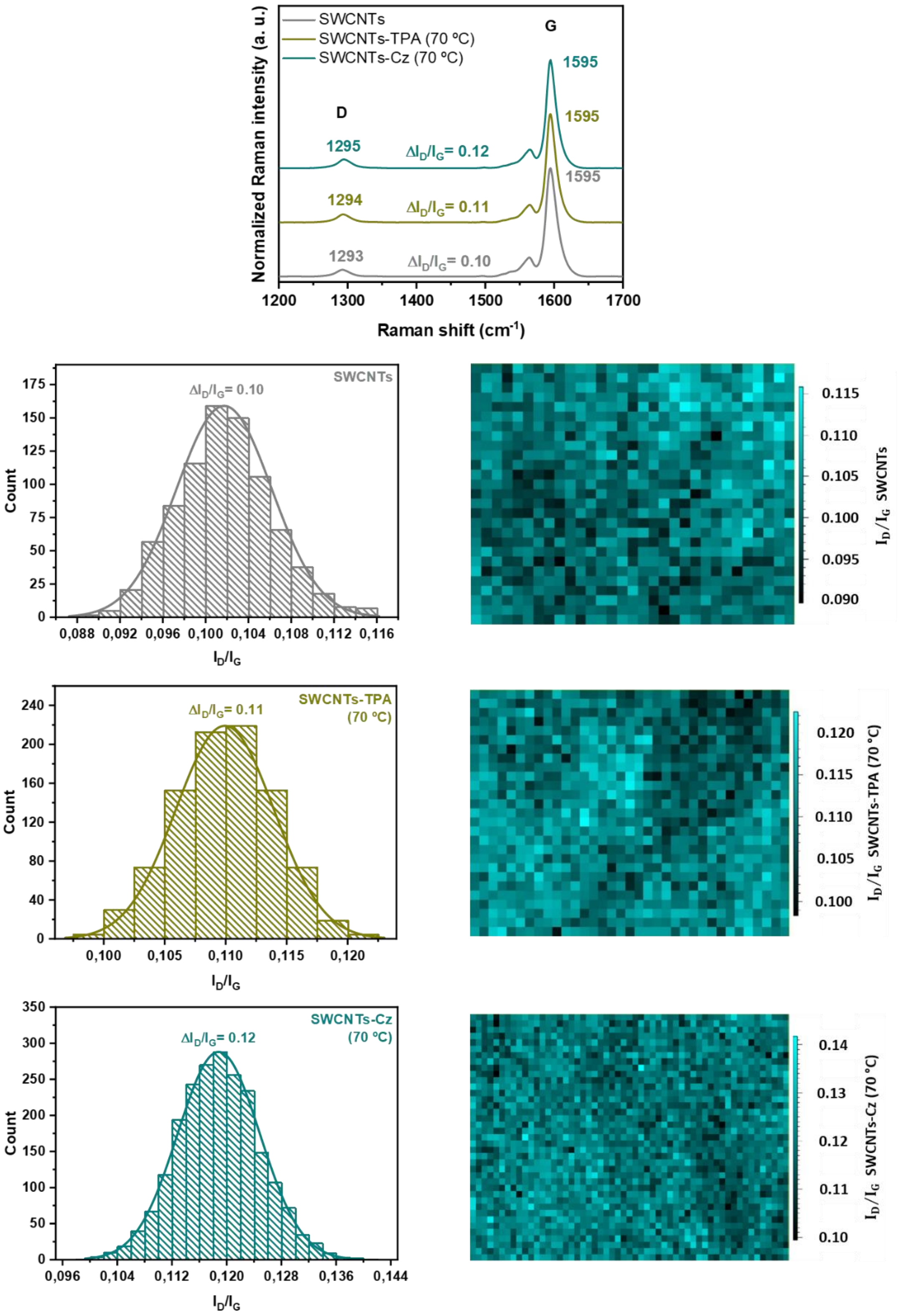

Figure S19. Raman average spectra of SWCNTs and the corresponding functionalized samples at 70 ºC (top). I_D/I_G histograms of pristine SWCNTs and the corresponding functionalized SWCNTs at 70 ºC (SWCNTs-TPA and SWCNTs-Cz) (left) and Raman maps on the right side of the figure. Data calculated from the average of > 700 individual measurements taken over the sample with an excitation wavelength of 785 nm.

2.2. Device characterization

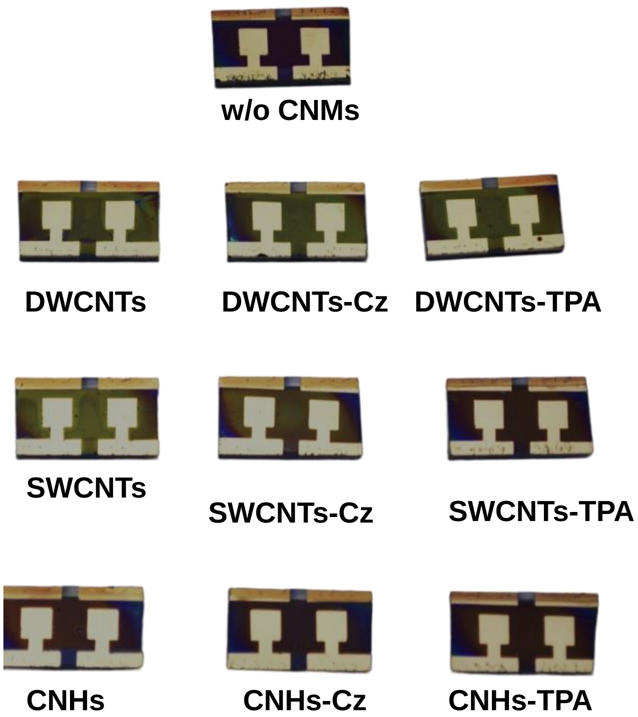

Figure S20. Perovskite solar cells with and without carbon nanomaterials (CNMs)

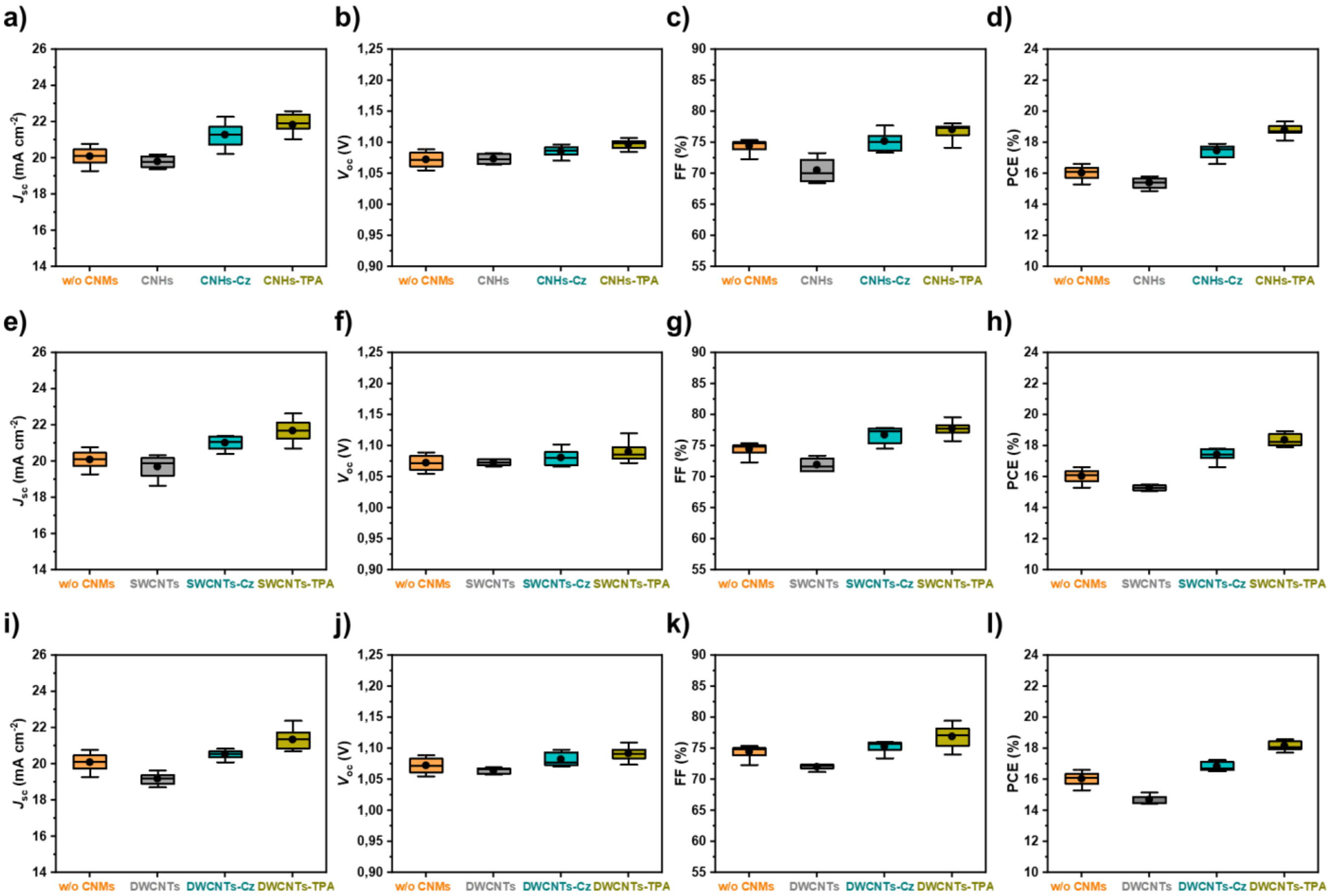

Figure S21. Statistical box charts of photovoltaic parameters (J_{SC}, V_{OC}, FF, and PCE) of perovskite solar cells (PSCs) with and without carbon nanomaterials (CNMs) in the hole transport layer (HTL). a-d represent box charts for cells

with CNHs, e-h for cells with SWCNTs, and i-l for cells with DWCNT derivatives.". Cells without CNMs are shown in orange, pristine carbon nanomaterials cells in grey, cells with carbazole (Cz) derivatives in cyan, and cells with triphenylamine (TPA) in olive color. Data corresponds to approximately 12 devices for each condition.

Table S3. Average values and standard error of photovoltaic parameters of perovskite solar cells (PSCs) with and without carbon nanomaterials (CNMs) in the hole transport layer (HTL).

HTL	Jsc (mA cm^{-2})	*Voc* (V)	FF (%)	PCE (%)
w/o CNMs	20.1 ± 0.5	1.07 ± 0.01	74.4 ± 0.9	16.0 ± 0.4
CNHs	19.8 ± 0.4	1.07 ± 0.01	71 ± 2	15.4 ± 0.4
CNHs-Cz	21.3 ± 0.6	1.09 ± 0.01	75 ± 1	17.5 ± 0.4
CNHs-TPA	21.8 ± 0.7	1.10 ± 0.01	77 ± 1	18.8 ± 0.3
SWCNTs	19.7 ± 0.7	1.07 ± 0.01	72 ± 1	15.3 ± 0.2
SWCNTs-Cz	21.0 ± 0.4	1.08 ± 0.01	77 ± 1	17.4 ± 0.4
SWCNTs-TPA	21.7 ± 0.6	1.09 ± 0.02	78 ± 1	18.4 ± 0.4
DWCNTs	19.2 ± 0.4	1.06 ± 0.01	72.0 ± 0.5	14.7 ± 0.3
DWCNTs-Cz	20.5 ± 0.2	1.08 ± 0.01	75.3 ± 0.9	16.8 ± 0.3
DWCNTs-TPA	21.3 ± 0.5	1.09 ± 0.01	77 ± 2	18.2 ± 0.3

Photovoltaic parameters, including short circuit current density (***J*****sc**), open circuit voltage (***V*****oc**), fill factor (**FF**), power conversion efficiency (**PCE**). Data corresponds to approximately 12 devices for each condition.

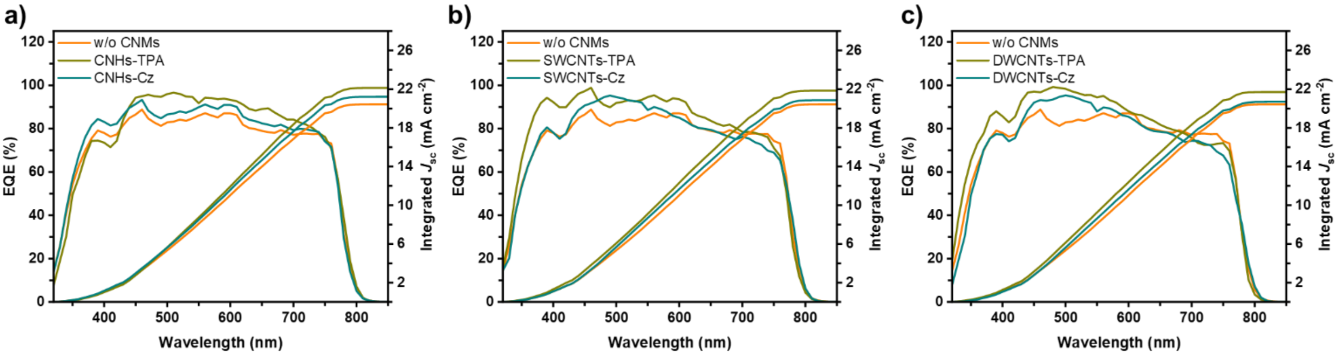

Figure S22. EQE spectra and integrated short-circuit current density (J_{SC}) of the perovskite solar cells (PSCs) with and without functionalized carbon nanomaterials (CNMs) in the hole transport layer (HTL).

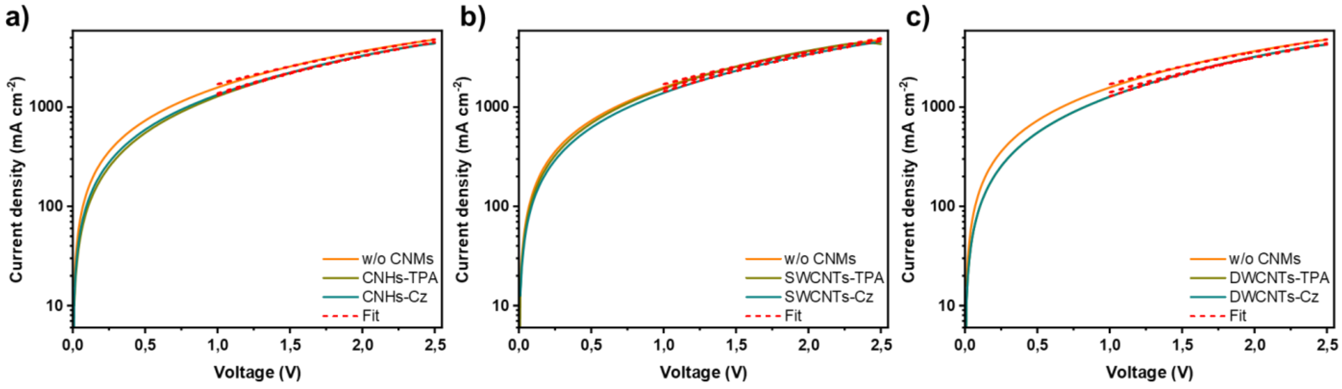

Figure S23. Space charge-limited current (SCLC) fitting of hole transport layer (HTL) with and without functionalized carbon nanomaterials (CNMs).

Table S4. The fitting parameters of PL decay curves of MAPI/HTL films with and without functionalized carbon nanomaterials (CNMs) in the hole transport layer (HTL).

MAPI/HTL	A_1	τ_1 (ns)	A_2	τ_2 (ns)	τ_{avg} (ns)
Bare MAPI	0.09	27.7	0.74	207.0	204.1
w/o CNMs	0.22	16.7	0.74	75.2	71.5
CNHs-TPA	0.39	10.8	0.70	41.1	37.2
CNHs-Cz	0.32	14.8	0.66	56.9	52.2
SWCNTs-TPA	0.36	11.2	0.65	41.4	37.5
SWCNTs-Cz	0.30	15.2	0.69	59.0	54.5
DWCNTs-TPA	0.28	11.0	0.72	40.8	38.0
DWCNTs-Cz	0.30	18.3	0.68	62.5	57.4

Fitting parameters including the amplitudes (**A_1** and **A_2**), lifetime (**τ_1** and **τ_2**) and the average lifetimes (**τ_{avg}**) which were calculated by means of $\tau_{avg} = \sum \alpha_i \tau_i$ where $\alpha_i = A_i \tau_i / \sum A_i \tau_i$

Table S5. Electronic properties of spiro-OMeTAD with and without functionalized carbon nanomaterials.

HTM	λ_{onset}[a] (nm)	ΔE[b] (eV)	E^1_{ox}[c] (V)	E_{HOMO}[d] (eV)	E_{LUMO}[e] (eV)
w/o CNMs	416	2.98	-0.045	-5.22	-2.24
CNHs-TPA	408	3.04	0.024	-5.29	-2.25
CNHs-Cz	412	3.01	-0.010	-5.26	-2.25
SWCNTs-TPA	409	3.03	0.011	-5.28	-2.25
SWCNTs-Cz	413	3.00	-0.012	-5.26	-2.26
DWCNTs-TPA	410	3.02	0.001	-5.27	-2.25
DWCNTs-Cz	413	3.00	-0.019	-5.25	-2.25

a) The wavelengths of absorption onset (**λ_{onset}**) were extracted from the UV-Vis absorption spectra presented in Figure 6. b) The energy of the optical band gaps (**ΔE**) were calculated using the formula $\Delta E = hc/\lambda_{onset}$, where h is Planck's constant and c is the speed of light. c) The first oxidation potentials (**E^1_{ox}**) were determined from the first half-wave in the cyclic voltammograms in Figure S23. d) The HOMO energy levels (**E_{HOMO}**) were estimated using the formula $E_{HOMO} = -e(E^1_{ox} + 5.27)$. e) The LUMO energy levels (**E_{LUMO}**) were estimated by means of the formula $E_{LUMO} = E_{HOMO} + \Delta E$.

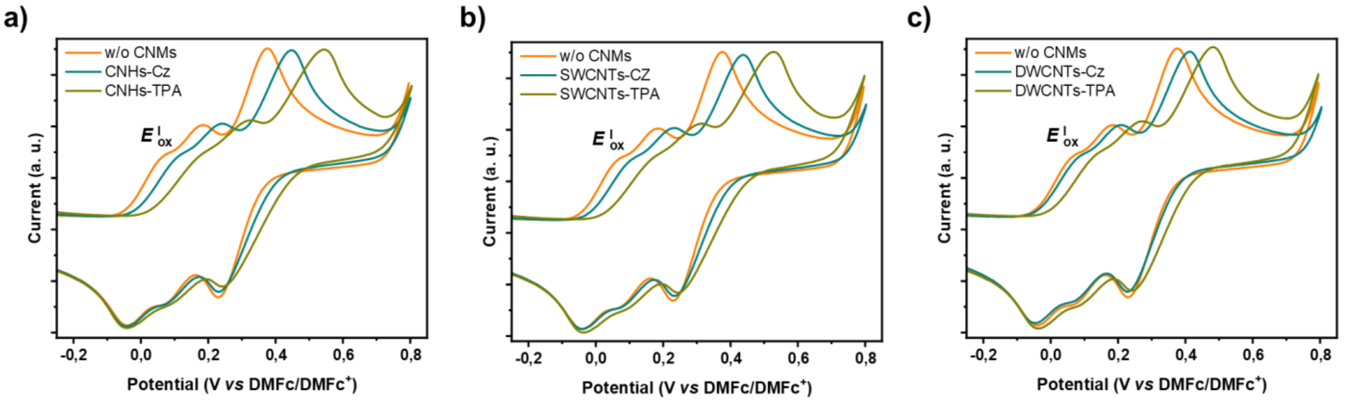

Figure S24. Cyclic voltammograms of spiro-OMeTAD with and without functionalized carbon nanomaterials a) CNHs derivative, b) SWCNTs derivatives, c) DWCNTs derivatives. Spiro-OMeTAD without CNMs is shown in orange, with carbazole (Cz) derivatives in cyan, and with triphenylamine (TPA) in olive color.

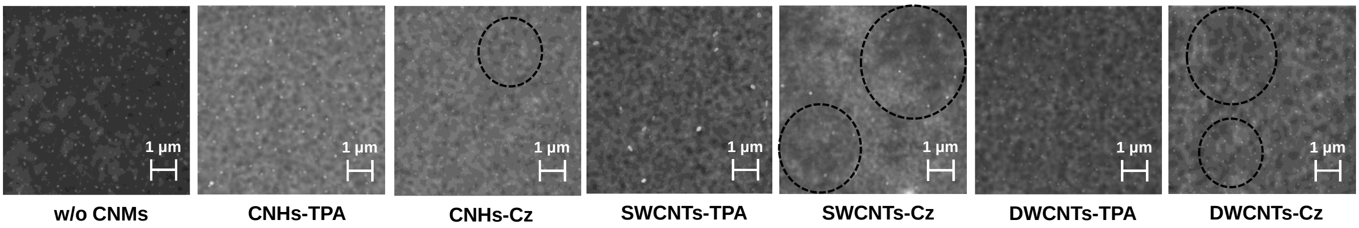

Figure S25. FE-SEM top-view images of spiro-OMeTAD-based HTL with and without functionalized carbon nanomaterials. The circles indicate the areas with darker tones.

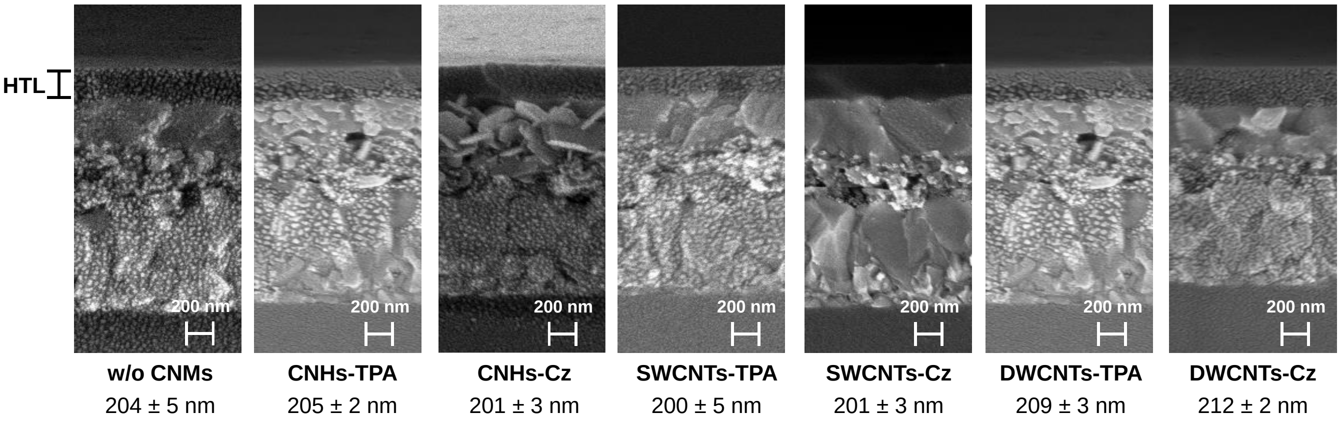

Figure S26. FE-SEM cross-section images of spiro-OMeTAD-based HTL with and without functionalized carbon nanomaterials. The values correspond to the HTL thickness.

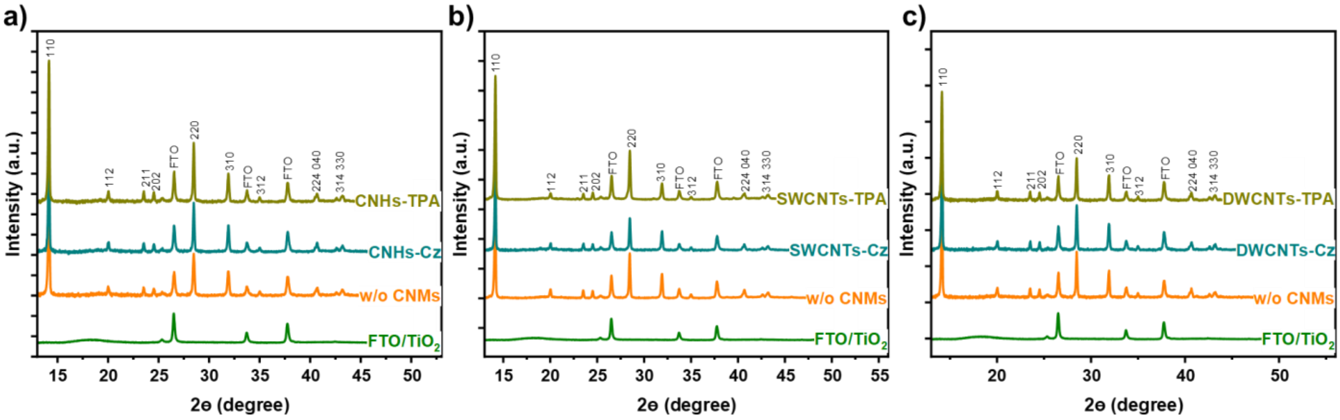

Figure S27. XRD patterns of FTO/c-TiO_2/m-TiO2/MAPI/spiro-OMeTAD-based HTM film with and without CNMs.

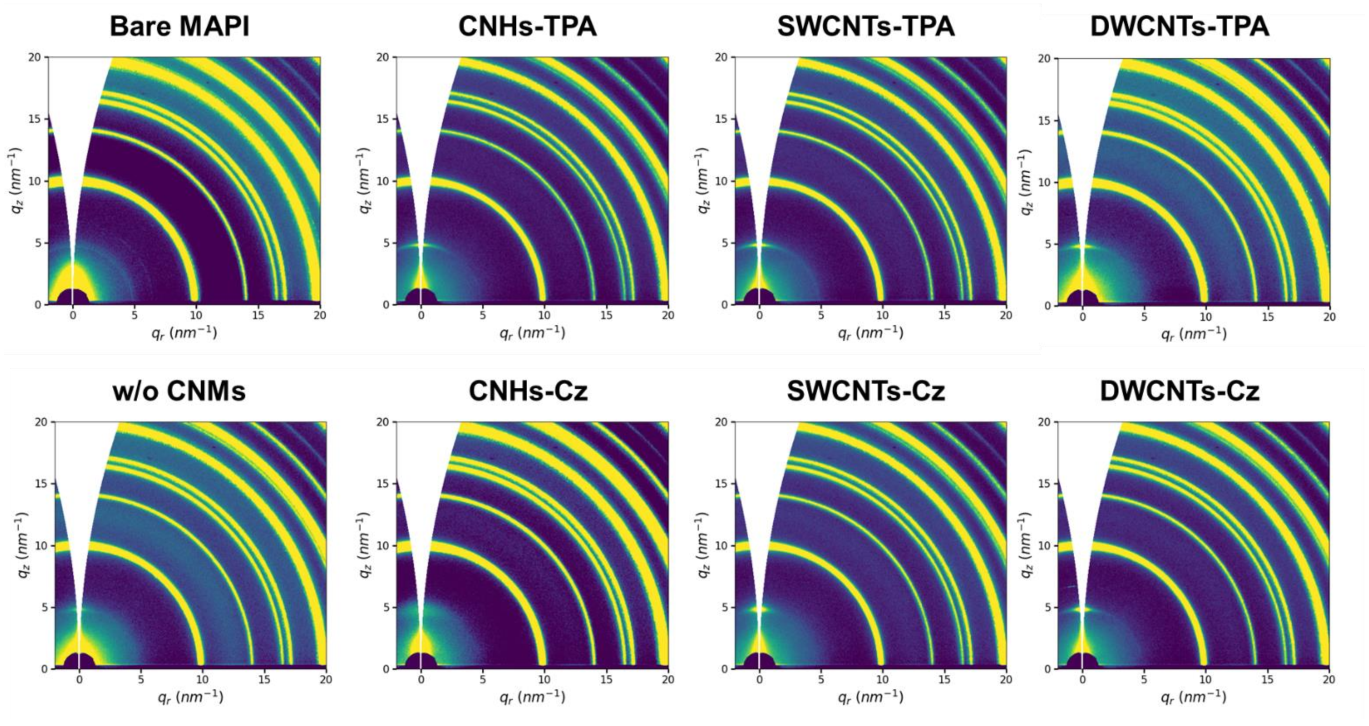

Figure S28. 2D GIWAXS patterns of FTO/c-TiO_2/m-TiO2/MAPI/spiro-OMeTAD-based HTM film with. Incident angle = 0.2°.

References

(1) Nakaie, N.; Nakazawa, T. Aniline Derivative, Charge-Transporting Varnish and Organic Electroluminescent Device. EP3012245B1, 2014.

(2) Zhang, Q.; Ning, Z.; Tian, H. “Click” Synthesis of Starburst Triphenylamine as Potential Emitting Material. *Dyes and Pigments* **2009**, *81* (1), 80–84. https://doi.org/10.1016/j.dyepig.2008.09.005.

(3) Lv, H.; Ma, R.; Zhang, X.; Li, M.; Wang, Y.; Wang, S.; Xing, G. Surfactant-Modulated Discriminative Sensing of HNO and H_2S with a Cu^{2+}-Complex-Based Fluorescent Probe. *Tetrahedron* **2016**, *72* (35), 5495–5501. https://doi.org/10.1016/j.tet.2016.07.039.

(4) Maeda, Y.; Saito, K.; Akamatsu, N.; Chiba, Y.; Ohno, S.; Okui, Y.; Yamada, M.; Hasegawa, T.; Kako, M.; Akasaka, T. Analysis of Functionalization Degree of Single-Walled Carbon Nanotubes Having Various Substituents. *J. Am. Chem. Soc.* **2012**, *134* (43), 18101–18108. https://doi.org/10.1021/ja308969p.

(5) Barrejón, M.; Rauti, R.; Ballerini, L.; Prato, M. Chemically Cross-Linked Carbon Nanotube Films Engineered to Control Neuronal Signaling. *ACS Nano* **2019**, *13* (8), 8879–8889. https://doi.org/10.1021/acsnano.9b02429.

(6) Juzgado, A.; Soldà, A.; Ostric, A.; Criado, A.; Valenti, G.; Rapino, S.; Conti, G.; Fracasso, G.; Paolucci, F.; Prato, M. Highly Sensitive Electrochemiluminescence Detection of a Prostate Cancer Biomarker. *J. Mater. Chem. B* **2017**, *5* (32), 6681–6687. https://doi.org/10.1039/C7TB01557G.
